# In Silico Reconstitution of Actin-Based Symmetry Breaking and Motility

**DOI:** 10.1371/journal.pbio.1000201

**Published:** 2009-09-22

**Authors:** Mark J. Dayel, Orkun Akin, Mark Landeryou, Viviana Risca, Alex Mogilner, R. Dyche Mullins

**Affiliations:** 1 Miller Institute for Basic Research in Science, University of California Berkeley, Berkeley, California, United States of America; 2 Department of Cellular and Molecular Pharmacology, University of California San Francisco, San Francisco, California, United States of America; 3 Department of Mechanical Engineering, University College London, London, United Kingdom; 4 Biophysics Graduate Group, University of California Berkeley, Berkeley, California, United States of America; 5 Department of Mathematics, University of California Davis, Davis, California, United States of America; 6 Department of Neurobiology, Physiology, and Behavior, University of California Davis, Davis, California, United States of America; Yale University, United States of America

## Abstract

Eukaryotic cells assemble viscoelastic networks of crosslinked actin filaments to control their shape, mechanical properties, and motility. One important class of actin network is nucleated by the Arp2/3 complex and drives both membrane protrusion at the leading edge of motile cells and intracellular motility of pathogens such as *Listeria monocytogenes*. These networks can be reconstituted in vitro from purified components to drive the motility of spherical micron-sized beads. An Elastic Gel model has been successful in explaining how these networks break symmetry, but how they produce directed motile force has been less clear. We have combined numerical simulations with in vitro experiments to reconstitute the behavior of these motile actin networks in silico using an Accumulative Particle-Spring (APS) model that builds on the Elastic Gel model, and demonstrates simple intuitive mechanisms for both symmetry breaking and sustained motility. The APS model explains observed transitions between smooth and pulsatile motion as well as subtle variations in network architecture caused by differences in geometry and conditions. Our findings also explain sideways symmetry breaking and motility of elongated beads, and show that elastic recoil, though important for symmetry breaking and pulsatile motion, is not necessary for smooth directional motility. The APS model demonstrates how a small number of viscoelastic network parameters and construction rules suffice to recapture the complex behavior of motile actin networks. The fact that the model not only mirrors our in vitro observations, but also makes novel predictions that we confirm by experiment, suggests that the model captures much of the essence of actin-based motility in this system.

## Abstract 2

Computational modeling and experimentation in a model system for actin-based force generation explain how actin networks initiate and maintain directional movement.

## Author Summary

Networks of actin filaments provide the force that drives eukaryotic cell movement. In a model system for this kind of force generation, a spherical bead coated with an actin nucleating protein builds and rockets around on an actin “comet tail,” much like the tails observed in some cellular systems. How does a spherically symmetric bead break the symmetry of the actin coat and begin to polymerize actin in a directional manner? A previous theoretical model successfully explained how symmetry breaks, but suggested that the subsequent motion was driven by actin squeezing the bead forwards—a prediction refuted by experiment. To understand how motility occurs, we created a parsimonious computer model that predicted novel experimental behaviors, then performed new experiments inspired by the model and confirmed these predictions. Our model demonstrates how the elastic properties of the actin network explain not only symmetry breaking, but also the details of subsequent motion and how the bead maintains direction.

## Introduction

The directed assembly of actin networks drives the motility of most eukaryotic cells [1]. Specialized cellular factors assemble actin into different network types, each with a unique architecture and cellular function [2]. One of the most well-studied actin assembly factors is the Arp2/3 complex, a seven-subunit protein complex that nucleates new filaments from the sides of pre-existing filaments to create entangled, dendritic filament arrays [3],[4]. These arrays behave like viscoelastic gels with an elasticity that depends on the degree of branching, and which break or rip under relatively low stress [5].

In vivo, dendritic networks built by Arp2/3 complex form the lamellipod that drives the movement of eukaryotic cells [3],[6] as well as the “comet tails” whose assembly drives the intracellular movement of endosomes [7],[8] and intracellular pathogens [9] such as Vaccinia virus [10] and *Listeria* [11]. Construction of these motile networks in vivo requires a set of highly conserved accessory proteins, including capping protein, cofilin, and profilin, that function together with the Arp2/3 complex in a simple biochemical cycle converting monomeric actin into crosslinked polymer and back again [6],[12]. Motile, dendritic actin networks can also be constructed in vitro by recombining purified components of the actin assembly cycle [13]–[16]. These reconstituted actin networks have become a powerful tool for studying how individual protein–protein interactions control the large-scale behaviors of cytoskeletal systems.

The simplest way to initiate assembly of such motile, dendritic actin networks in vitro is the “bead motility” system, in which micron-sized beads are uniformly coated with factors that activate the Arp2/3 complex to nucleate actin networks at their surfaces [16],[17]. These networks form spherically symmetric shells that eventually “break symmetry” and produce stable, asymmetric comet tails that propel the bead along, maintaining direction [14],[16],[18], moving smoothly or pulsing depending on conditions [19],[20]. In this work, we concentrate on how a geometrically and biochemically symmetric bead can first break symmetry then maintain asymmetry to produce directed smooth or pulsatile motion.

Spatially localized nucleation of actin filaments combined with global inhibition of filament elongation by capping protein restricts filament growth to a well-defined zone, e.g., the *Listerium* surface [21], lamellipodial plasma membrane [22], etc. On the spatial scale of filaments, a Brownian ratchet mechanism has been proposed [23],[24] to explain how actin polymerization uses the energy of ATP hydrolysis to rectify Brownian fluctuations, exerting force at the surface, as new actin monomers, as new actin monomers add onto existing filaments and extend the network. Although the specific details may vary [25]–[27], spatially localized network extension fueled by ATP hydrolysis is the basis of all polymerization-driven motility models.

Several theoretical frameworks have been proposed to explain actin-based symmetry breaking and bead motility (reviewed in [28]). Some are based on filament-scale descriptions of actin assembly and crosslinking [29],[30], while others take a more coarse-grained approach based on the bulk mechanical properties of crosslinked polymer networks [17],[19],[20],[31]–[34]. One such coarse-grained model is the Elastic Gel model [19],[31], which provides an intuitive explanation for symmetry breaking. In this model, symmetry breaking occurs when new actin network, continuously deposited at the surface of the bead, displaces older portions of the network radially outward. Expansion of the older network stretches it like the surface of an inflating balloon until, at a critical threshold, circumferential stress causes a rupture in the network (either by melting [33] or cracking [35] the shell) and breaks the symmetry of the system. This mechanism fits the experimental observations of symmetry breaking [16],[19] better than mechanisms inferred from filament-based descriptions of the network [30]. Pulsatile motion has been suggested to result from an unstable balance between the pushing forces and the drag from attached filaments [20].

Explaining the smooth directional motility of symmetrically coated beads has proved more challenging. One attempt, the Soap-Squeezing model [31], is an extension of the Elastic Gel model that offers an explanation of propulsive force. In this model, surface-associated polymerization stretches older network outwards orthogonal to the direction of motion, storing energy, which it releases by contracting orthogonally, squeezing the bead forward like a hand squeezing a wet bar of soap. However, photobleaching data showing the movement of the network as it leaves the bead demonstrate that orthogonal squeezing does not occur [17], and whereas treating the network as an incompressible fluid flowing from the bead surface can explain the observed motion [17], this violates the elastic nature of the gel required to explain the initial symmetry breaking. How, then, does sustained motility occur?

In this paper, we examine the essence of actin-based bead motility by reconstituting it in silico from the network's fundamental viscoelastic properties. Just as reconstituting actin-based motility in vitro from a minimal set of purified protein components demonstrates their necessity and can show how they contribute to the large-scale behavior, reconstituting actin-based motility in silico allows us to demonstrate the necessity and specific contributions of a minimal set of higher-level network properties (e.g., elasticity, crosslinking, etc.), and demonstrate the mechanisms of motility on a mesoscopic scale. To do this, we use a framework we call the Accumulative Particle-Spring model (APS model) in which the viscoelastic actin network is represented simply as a set of particles, subject to viscous drag and coupled by springs that break when strained beyond a certain limit. New Particle-Spring network is created at the bead surface, just as the in vitro actin network polymerizes at the bead surface [16], and we find that this simple system is sufficient to reproduce a range of the behaviors of actin networks, including symmetry breaking and motility.

Our simulations enable us to explore the feasibility of hypothesized mechanisms of force and movement generation, using Ockham's razor to determine the essence of the behavior by exploring the minimal requirements to produce the observed results. We validate the model by checking the results and predictions of the simulations with in vitro experiments in which we reconstitute symmetry breaking and motility from purified proteins. To the extent that the model is valid, we are able to make explanatory claims for the mechanisms involved in symmetry breaking and motility, determining 1) the stress and strain distributions in a growing symmetric actin shell and in a comet-like tail, 2) where the symmetry break is initiated (outer or inner surface of the actin shell), 3) the 3-D structure and dynamics of the break, 4) what determines the transition from smooth to pulsatile motility, and 5) how symmetry breaking occurs for nonspherical objects.

## Results

### Viscoelastic Forces Drive Bead Motility

**Figure 1:**
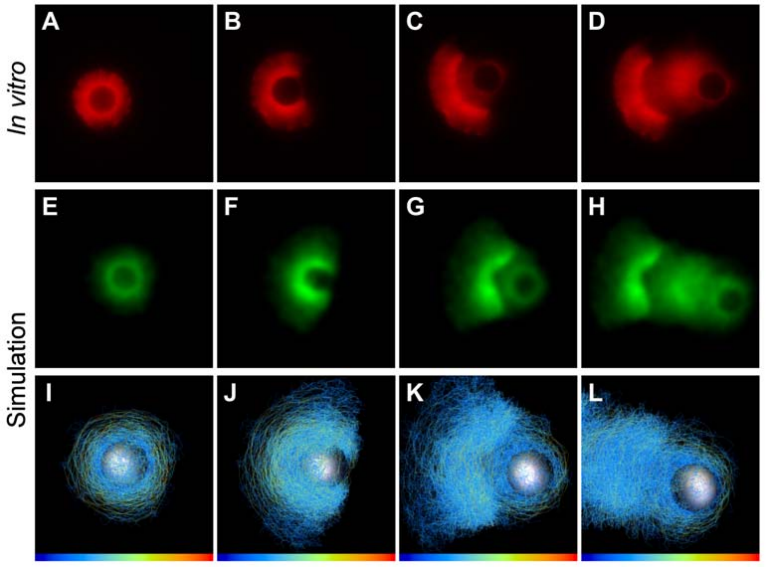
Simulations qualitatively mimic in vitro symmetry breaking and bead motility. (A–D) Time series of in vitro symmetry breaking and motility for beads uniformly coated with ActA (see Video S1). (E–H) Time series of a computer simulation of symmetry breaking and motility (2-D projections convolved with Gaussian, projection plane chosen parallel to shell opening; see Video S2). (I–L) 3-D view of simulation showing links colored by tensile stress (see Video S3; color bar range represents zero [blue] to breakage stress [red]). A–D correspond to 70s, 106s, 175s and 344s. E–H and I–J correspond to frames 70, 134, 185 and 330 of the simulation (see Figure 3 for detailed kinetics).

To perform our in vitro bead motility experiments, we evenly coated 5-µm diameter beads with ActA and added them to motility mix (see Materials and Methods). ActA activates Arp2/3 to nucleate an actin network that grows in a tightly localized zone at the bead surface, breaks symmetry, and propels the bead on an actin comet tail (Figure 1A–1D and Video S1).

To find out how well bead motility can be explained simply by the viscoelastic properties of the network, we created a computational model that simulates the behavior of a generic viscoelastic network deposited stochastically at the surface of a bead. The model starts at *t* = 0 with no network, then nucleates nodes at a constant rate and with an even distribution across the bead surface, crosslinking new nodes to their neighbors with links that behave as simple Hookean springs that break if extended too far (Figures S1 and S2). See Materials and Methods and Section S1 of the supporting text (Protocol S1) for full details of the model, and Tables S1 and S2 for the experimental bases for the model assumptions. We tuned the model parameters (spring constant, crosslinking probability, etc.) to produce qualitatively similar observations to the in vitro system (see Model Robustness, Section S3 of the supporting text (Protocol S1) for the effects of varying each parameter. Table S3 lists the corresponding names in the code for simulation parameters mentioned in the main text). This simple model exhibits both symmetry breaking and motility behavior that reproduces the sequence of events seen in vitro (Figure 1E–1H, Video S2).

Our experimental observations and our simulations share several features. As the shell grows, it becomes denser near the surface of the bead. When the thickness of the shell reaches approximately the radius of the bead, a clear crack develops, and the bead exits the shell, then the shell opens, crescent-like, and motility proceeds, leaving a low-density and somewhat irregular comet-like tail behind the bead. Figure 1I–1L show the underlying 3-D nature of the simulated network, with the network links colored by tensile stress (Videos S3 and S4).

### Geometry of Symmetry Breaking

Although the simulations share many of the features of the experiments, we noticed that the shell shows a close to perfect arc for the experimental conditions in Figure 1, but the simulations robustly show a more V-like shape with a dent in the center of the inner high-density region of the shell (compare Figure 1C and 1D with 1G and 1H). This implies either a failure of the simulation to capture an essential behavior of the network, or a condition of the in vitro system that we did not include in the simulations.

**Figure 2:**
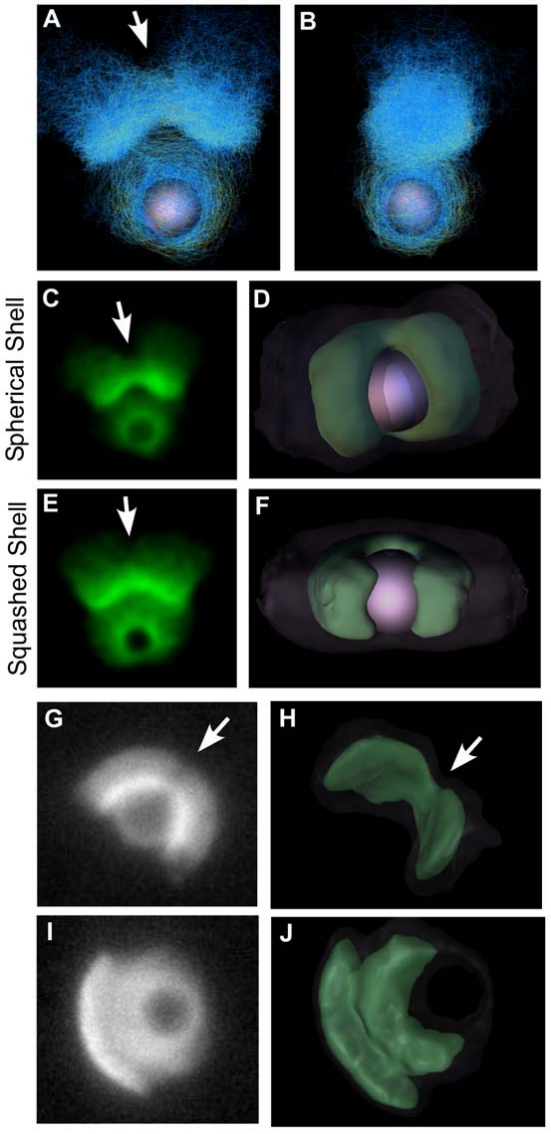
The 3-D geometry of symmetry breaking. (A and B) Top and side views of simulated network shortly after symmetry breaking showing that symmetry breaking is in one axis only. (C) 2-D projection of unconstrained simulation after symmetry breaking shows dent in the center of the shell. (D) 3-D isosurface representation of network and bead during symmetry breaking shows linear crack. (E and F) Same as (C and D), but network is constrained in *z*-direction between two parallel planes to mimic experimental conditions (note lesser dent in shell). (G–J) Projections and 3-D reconstructions of experimental data after symmetry breaking. (G and H) show a 5-µm bead with 15.5-µm spacers, (I and J) a 5-µm bead with 5.1-µm spacers. Arrows in (A–H) indicate rip in outer shell. See Figures S4, S5, and S6 for interactive 3D views.

To determine the cause of the dent, we examined the 3-D mechanics of symmetry breaking in our simulations. Figure 2A and 2B show 3-D top and side views of a representative simulated shell after the bead has moved away from the shell, demonstrating that even though the bead is unconstrained in three dimensions, the symmetry break and shell opening occur along only one axis. A rip in the outer shell often accompanies the dent, as seen in Figure 2A (arrow) and the corresponding 2-D projection view shown in Figure 2C. To understand why symmetry breaking occurs within one plane, we looked at how the shell cracks. Figure 2-D shows an earlier 3-D view of the same simulation, just as the crack completely fractures the shell; isosurfaces show the densest region of the network in green to highlight the shape of the shell, and the extent of the lower-density actin network (semitransparent). The symmetry-breaking crack is a straight line, as opposed to either lightning-like fracture(s) along the weakest regions of the network, or a circular hole opening to allow the bead to escape. The consequence of this straight-line break is that the 3-D stresses in the network are relieved in a 2-D manner—essentially splitting the 3-D spherical shell into two hemispheres that open apart from one another like a clamshell, causing large stresses at the hinge. When this 3-D geometry is viewed from above, the hinge appears as a dent, seen in Figure 2A and 2C. The crack that opens the two hemispheres often continues all the way around the outer network, resulting in the rip in the outer shell that accompanies the dent. For only one rip to occur, as soon as a crack begins, circumferential tension must relax quickly around the bead before a second crack begins. We can reduce this relaxation around the bead by increasing the strength of attachments with the nucleator (Figure S16), which prevents the network moving relative to the bead and makes the second crack progressively more prominent.

For the experimental conditions in Figure 1, we had intentionally confined the bead closely between a slide and coverslip to prevent it moving out of focus while we took data. Having seen how the crack propagates around the bead in the simulations, we hypothesized that the lack of a dent seen in the experiments might be a result of this constraint on the network preventing the crack propagating to the rear of the bead. To test this, we ran the same simulation while constraining the network between two planes (we also excluded nucleation from the very top and bottom 10% of the bead to prevent artifacts caused by this material having nowhere to go). Figure 2E and 2F correspond to 2C and 2D, but for this constrained shell (interactive 3-D representations are included in Figure S4). The constraint creates a toroidal shell that also breaks in a straight-line crack, but unlike the breaking of the spherical shell, the broken toroidal shell relaxes into a much more perfect arc, with the dent much reduced and the shell more closely resembling those seen in the experiments.

If our simulations are a valid model for the behavior of the actin network, they predict that if we were to perform the symmetry-breaking experiment in an unconstrained 3-D volume in vitro, it would produce a clamshell break with a dent in the shell opposite the break site as we see in the simulations. To test this, we performed the in vitro experiment using 5-µm diameter ActA-coated beads while controlling the headspace of the reaction with glass spacer beads of either 5.1-µm diameter for the constrained condition or 15.5 µm for the unconstrained condition. Because the 3-D shell structure is hard to interpret from a single 2-D microscope image, we reconstructed the 3-D shells from confocal *z*-stacks. We fixed the reaction after symmetry breaking (see Materials and Methods) to prevent movement while the *z*-stack was acquired; so for experiments, we are only able to capture the 3-D geometry at one time point after symmetry breaking has occurred, in contrast to having every time point in the simulations. Figure 2G and 2H show an example of a 2-D projection and 3-D reconstruction of a confocal stack of an unconstrained bead, confirming the distinctive bilobed structure, and V-shaped shell with central dent. Figure 2I and 2J similarly show the constrained condition with the near-perfect arc. (Beads tend to settle by gravity so that the tail and wide axis of shell are parallel to the coverslip, with shell cracks in the *z*-direction.) Figures S5 and S6 contain further examples of 2-D projections and 3-D reconstructions of symmetry breaking. Shell geometry for constrained beads was extremely consistent, always showing the near-perfect arc. Unconstrained beads showed less regularity, but always showed shells with shapes consistent with linear cracks; on one occasion, we observed a shell with a three-way opening (Figure S6B).

### Shell Deformations during Symmetry Breaking

**Figure 3:**
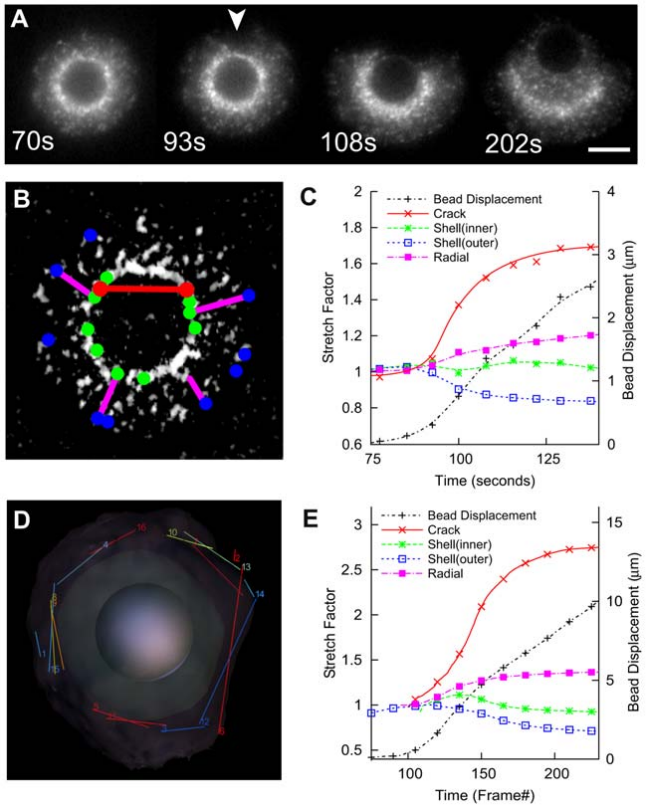
Shell deformations during symmetry breaking in vitro and in silico. (A) Fluorescent speckle microscopy (FSM) of in vitro symmetry breaking, time points as indicated (see Video S5). Arrowhead indicates initial rip in shell. (B) Diagram showing how geometric parameters are extracted from FSM data. Lengths between point pairs are plotted in (C). (C) Geometric parameters of in vitro symmetry breaking show outer circumferential contraction and radial expansion. Colors correspond to (B). Initial lengths prior to symmetry breaking are normalized to one. (D) Diagram showing how measurements are extracted from simulation (see Video S6) (for clarity, only outer circumferential measures shown). Points that span the crack are not included in circumferential measures; other measures are similar and correspond to those in (C). (E) Geometric parameters of in silico symmetry breaking show outer circumferential contraction and radial expansion similar to (C). Initial lengths prior to symmetry breaking are normalized to one.

To confirm that the mechanics of symmetry breaking in our simulations reflect those seen in vitro, we tracked the deformations of the shell during in vitro symmetry breaking using fluorescent speckle microscopy (Figure 3A, Video S5). Low doping of fluorescent actin produces fiduciary marks that allow us to measure the mechanical deformations of the network [36]. We tracked five parameters: bead displacement, expansion of the crack, circumferential stretching of the inner shell, circumferential stretching of the outer shell, and radial stretching of the shell (Figure 3B and 3C). When symmetry breaks, the crack opens rapidly and then slows as the shell approaches its final shape. As the shell opens, the outer circumference contracts with kinetics that mirror the crack opening, but the inner shell remains approximately the same circumference, merely reducing its curvature. As the shell opens, it also becomes thicker, with the kinetics of radial expansion mirroring the circumferential contraction and crack opening (magenta and blue lines in the graphs in Figure 3C).

We plotted similar parameters for a simulation run. We measured the 3-D distance between pairs of points approximately 2 µm apart (e.g., in the circumferential direction; Figure 3-D and Videos S6 and S7). The mechanics of the simulations behave like the in vitro experiments, with the crack opening rapidly, the outer circumference of the shell contracting and the shell becoming radially thicker, all with similar kinetics. The values of the Poisson's ratios differ a little, approximately 0.2 for the in vitro shell and approximately 0.3 for the simulation, likely resulting from simplifications in the functional forms for the link and repulsive forces (previous theoretical models have assumed a wide range of Poisson ratios, from 0 to 0.5 [31],[33],[37]). Also, the behavior of the inner shell differs slightly between experiment and simulation, with the circumference transiently expanding slightly (frame 140) before returning to its original length, whereas in vitro, the length remains constant. This most likely reflects transient disequilibrium during the most rapid part of the symmetry breaking, which is equilibrated more quickly in vitro than in the simulations. The current model therefore reproduces the qualitative behavior of the experiments but requires calibration in future work before it would be able to match quantitative measures. (N.B. For convenience, we note that 1 s corresponds to approximately 1.4 frames, but stress that this is not extensively kinetically calibrated.)

### Mechanics of Symmetry Breaking

**Figure 4:**
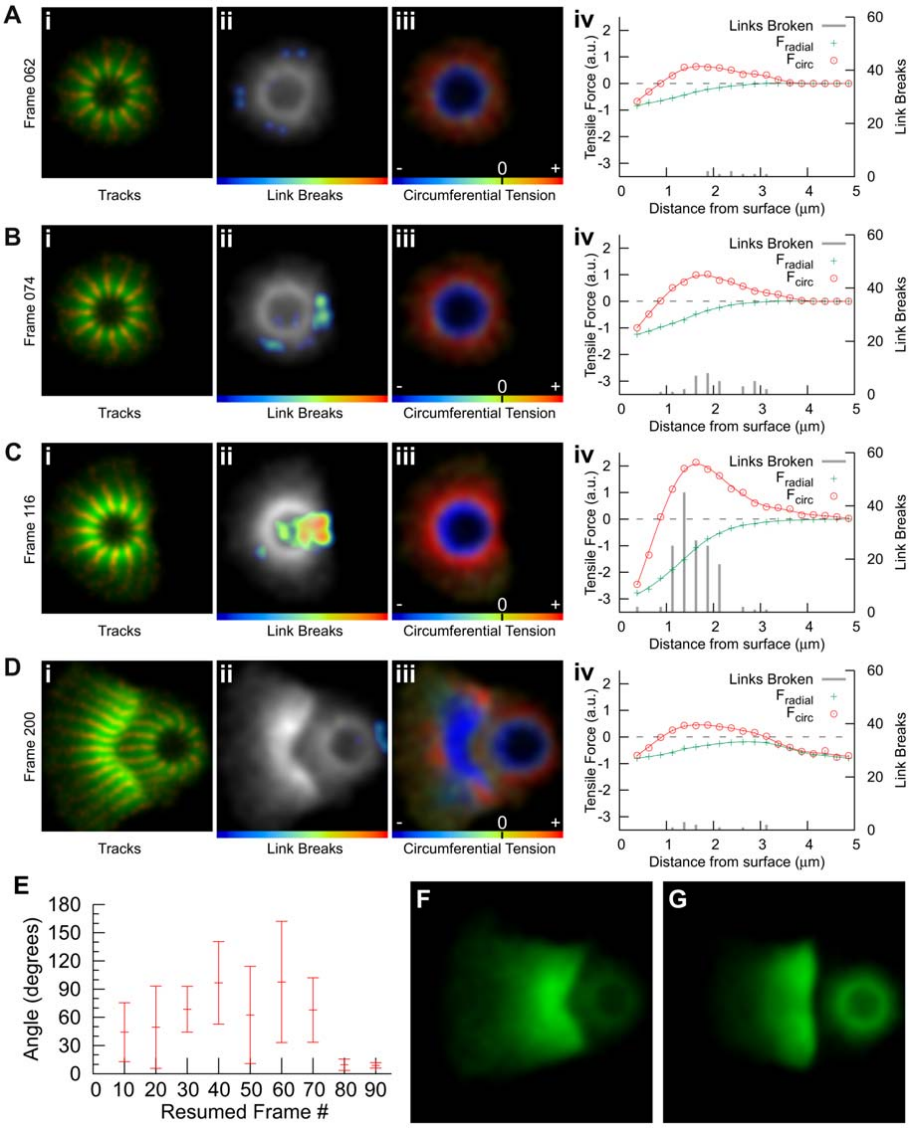
The mechanism of symmetry breaking. (A–D) Strain buildup and release by link breakage (see Video S8). Four time points showing (i) node tracks, (ii) link breaks, (iii) circumferential tension, and (iv) graphs showing how circumferential tension, radial tension, and link breaks vary with distance from the surface of the bead. For link breaks in (ii), color scale bar represents increasing density to the right (red). For circumferential tension in (iii), scale bar represents increasing tension to the right (red) with the black notch representing zero, and the left representing negative tension (i.e., compression) in blue. In (iv) forces are summed and split into radial and circumferential components. (E) Symmetry-breaking direction is determined late. One simulation was repeated, restarting at frames shown, and the angle of the new symmetry-breaking direction calculated relative to the original direction (mean±standard deviation, *n* = 5). The directions are essentially random until frame 80, after which they become the same as the original run, showing that the direction is determined between frames 70 and 80. This corresponds to a shell similar to the time point shown in (B). (F) Decreasing the network spring constant increases the thickness of the shell (*F*_L_ = 1.5 pN). (G) Increasing the threshold for link breakage produces a flat shell (*F*_BL_ = 5.5 pN). (Units are nominal—see text.)

Our simulations provide detailed information about the mechanism of symmetry breaking, e.g., the network motion, distribution of forces and ripping of the network (Figure 4A–4D, Video S8). In the left panels (Figure 4A(i)–4D(i)), we colored the regions of the network with red stripes to show the trajectory of the network as it moves away from the bead surface. Initially (frames 1–60), this pattern is radially symmetric—broken links occur randomly around the surface, giving no indication of the future site of symmetry breaking (link breaks are stochastic, see Video S8(ii) and Video S11). By Frame 62 (Figure 4A), the nodes around the future crack site have begun to diverge (Figure 4A(i)), followed by a burst of localized link breaks at the site (Figure 4B(ii)). This weakens the network, causing stress in that region to be distributed over fewer remaining links, leading to more breaks by positive feedback (Figure 4C(ii)), followed by the bead moving off with links breaking primarily at the front (Figure 4D(ii), Video S12).

To determine the force balance that contributes to shell formation and symmetry breaking, we examined the spatial distribution of stresses within the network. The right-hand graphs (Figure 4A(iv)–4D(iv), Video S8(iv)) show how the radial and circumferential tensions vary with distance from the surface of the bead (negative tension corresponds to compression), and the center panels (Figure 4A(iii)–4D(iii)) show the spatial distribution of circumferential tension. These are calculated as sums of the link tension forces (positive) and the node–node repulsion forces (negative), split into radial and circumferential components (individual components are graphed in Video S9; we exclude the data point nearest the bead because of surface artifacts caused by the way we deal with nodes that enter the nucleator, see Video S10 for full data). Both radial and circumferential tensions are negative at the bead surface, i.e., the center of the shell is under compression, the inner compressive forces balancing the outer circumferential tension. For small network distortions (close to the surface), the network equilibrates this compressive force primarily through the isotropic node–node repulsions, so the compression is not restricted to the radial component. Close to the bead surface, circumferential tension is lower (as predicted by the Elastic Gel model), so the compressive force is greater than the tension force (and the overall tensile force is negative).

Circumferential tension increases rapidly with distance from the bead (Figure 4C(iv)), becoming positive at approximately 1.0 µm, with the maximum tension approximately 1.5 µm from the surface, and tailing off at higher distances as the network becomes sparse. This distribution of forces can be clearly seen when the symmetry break begins (Figure 4C(iii)) as a red band of maximal circumferential network tension at approximately 1.5 µm encloses a blue band of maximal network compression at the bead surface. The distribution remains relatively static over time as forces build up (Figure 4A(iv)–4C(iv)), although the magnitudes of the forces change, with the maxima occurring when symmetry breaking begins (Figure 4C(iv)). These data support the Elastic Gel model for symmetry breaking: as the network is pushed out by nucleation at the center, it expands in the circumferential direction like a balloon, creating circumferential tension. Network compression close to the surface provides the balancing force for this circumferential tension—and because the expanding layers of network pull the network apart circumferentially, but not radially, the resulting radial forces are always compressive (negative tension in the graphs in Figure 4A(iv)–4D(iv)). The release of tensile energy upon symmetry breaking can be vividly seen between Figure 4C(iii) and 4D(iii)—the shell opens and pulls back away from the bead, contracting circumferentially and releasing the energy stored in circumferential tension—much of the red region of maximum circumferential tension in Figure 4C(iii) turns blue (compression) in Figure 4D(iii), Video S8.

Small defects in the outer shell have been proposed to establish the site of symmetry breaking [32],[33]. We can determine when the symmetry breaking site is established in our simulations relatively easily. In our simulations, we add new network stochastically at the bead surface—this randomness results in a unique network and symmetry-breaking direction for each run. For each run, we save a complete description of the system at each time point, and can resume the run at any point with a different random seed. To discover the time at which the symmetry-breaking direction is determined, we ran a simulation through to symmetry breaking, then rewound and restarted the same simulation from nine different time points, but with a different random seed. We repeated this set of nine runs five times to calculate the mean and standard deviation of the angle between the new symmetry-breaking direction and the original direction (Figure 4E). This produces a high variance in symmetry-breaking direction before the direction is determined, and both very low variance and a close to zero deviance angle afterwards. We find the symmetry-breaking direction is essentially random until frame 80, at which point the direction becomes the same as the original run. Symmetry-breaking direction is therefore determined between frames 70 and 80, i.e., very late—just before symmetry breaks—rather than being determined early by defects in the initial outer network.

Our simulations also show that the force balance and pattern of link breaks in the outer network before symmetry breaking define the final curvature of the shell after symmetry has broken. Figure 4F shows that halving the spring constant (the *F*_L_ parameter) causes the shell to double in thickness, and Figure 4G shows that increasing the threshold force for link breakage (the *F*_BL_ parameter in the simulation) causes the shell to become flat (see also Figures S13 and S12). These results follow from the Elastic Gel model: decreasing the spring constant between links of the network will require that more material be deposited to build up enough circumferential tension for symmetry to break, so the shell is thicker. Also, the final curvature of the shell after recoil is dependent on the number of links that have broken in the outer shell during the earlier stages of shell buildup. Without breaks in the outer shell, the final equilibrium area of the outer shell is still the same as the inner, so the resulting shell is flat. The more links that break in the outer network, the larger its equilibrium area, and the higher the resulting curvature. These parameters and others are more thoroughly explored in Model Robustness, Section S3 of the supporting text (Protocol S1).

### Symmetry Breaking and Network Plasticity

Symmetry breaking is a particularly robust behavior of our model. Of the parameters tested, those that do not break symmetry are those that set network link density to extremes (Figures S10, S11, S12, S13, S14, S15, S16, S17, S18, S19, and S20). One extreme creates a very strong network that builds a dense shell that never breaks symmetry, by creating conditions in which the network strength increases faster than the network strain, e.g., when we increase the threshold for link breakage (Figure S12). The other extreme creates a very weak network in which symmetry does not break because chains of links are too short to communicate tension around the bead, so the network remains unpolarized, seen by decreasing the crosslinking probability, or decreasing the link-breaking threshold (Figures S11 and S13).

Our model network is constructed from nodes and links that are short compared to the size of the bead—to transmit force around the bead, there must be enough links to form chains spanning around the bead. The “mesh size” characterizes the length scale of the network formed from these chains of links, referring to the minimum size of a particle that would be trapped by a network made of these chains. In our case, if the mesh size is greater than the size of the bead, the bead would be able to move *through* the network, so it would not be possible to build up tension in the shell, and there would not be a clean symmetry break. For our purposes, we define network coherency as the bead size divided by the mesh size, i.e., high network coherency means that the bead will see the network as an elastic solid, whereas low coherency means the bead would be able to squeeze through the network.

We find that even a low level of network coherency is sufficient to support symmetry breaking, the key is that tension is transmitted around the bead. This kind of symmetry breaking does not involve a distinct shell that cracks, but rather a gradual oozing of the bead from a network cloud (Figures S11 and S13). This oozing demonstrates a qualitative change in behavior that results from the quantitative change in degree of crosslinking. When a sparsely linked network deforms, it undergoes plastic flow as energy is lost by links breaking independently, whereas when a dense network deforms, it builds up elastic energy, as each link stretches slightly while remaining below its breaking strain. Eventually, this dense network undergoes brittle fracture when many links break at once.

The initial shell shows a gradient of network density increasing from the outer to the inner surface of the shell both in vitro and in silico. This density gradient emerges spontaneously from the APS model as a result of the increasing circumferential tension in the outer shell compressing the inner shell. The initial outer network is sparse because it is not under compression, so the network has a low density of links (since links are formed to nearby nodes, and a sparse network means fewer nodes nearby). This sparse initial outer network is weak and plastic but does provide enough compression on the inner network to cause an increase in density, hence a greater number of links, and a stronger network, which builds by positive feedback. As demonstrated in Figure 4A–4D, which shows a peak in circumferential tension towards the center at around 1.5 µm from the surface, it is this inner brittle network that stores the bulk of the elastic energy, and undergoes brittle fracture during symmetry breaking.

### Network Deformations during Smooth Motility

**Figure 5:**
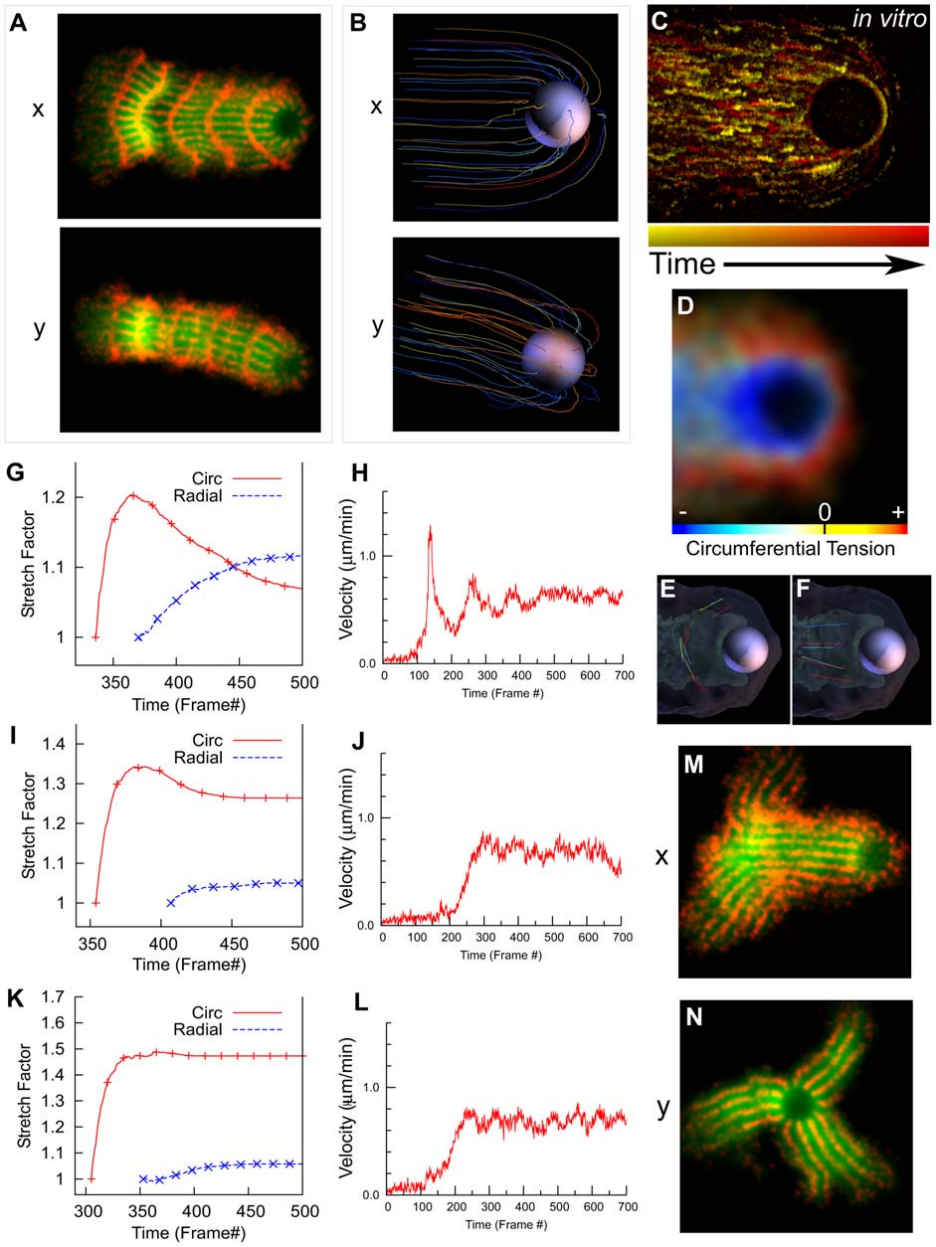
The mechanism of smooth motility. (A) Orthogonal 2-D views of in silico network trajectory, with network marked red at even intervals of time and position around the bead. Nodes in the 3-D network are convolved with a Gaussian and projected in *x* or *y* directions as shown. (B) Orthogonal 3-D views of the network trajectory show linear ripping at front and no orthogonal squeezing. Lines represent trajectories relative to the bead for an evenly distributed subset of nodes. (C) In vitro network tracks during smooth motility showing no orthogonal squeezing. Image is a composite of sequential fluorescent speckle microscopy images (see Video S13) colored by time and registered to the bead. (D) Distribution of circumferential tension (red) and compression (blue) around in silico bead during smooth motility. Circumferential tension is localized to the outer network and front of bead. (E and F) Diagram of how circumferential and radial measurements for smooth motion were taken. Measurements exclude points in front of the bead where the rip occurs (see Videos S14 and S15). (G–L) Network stretching and bead velocity for 3 regimes of smooth motility: (G and H) elastic network (default parameters), (I and J) less elastic network (*R*_M_ = 5.0 pN, *F*_BL_ = 2.0 pN, and *F*_L_ = 4.0 pN), and (K and L) less elastic network with network locked in place before circumferential contraction occurs. (M and N) Orthogonal views and of symmetry breaking and motility from (I and J; see Videos S16 and S17). (Units are nominal—see text.)

In both our experiments and simulations, the bead continues to move after breaking symmetry. To investigate the motility mechanism, we examined network movement by plotting orthogonal views of the network trajectory for a simulation of smooth motion (Figure 5A). To show the network trajectory, we marked the network with a spatiotemporal grid, coloring it red when it originated at evenly spaced locations around the bead (the parallel lines in the tail), and at even time intervals during the run (the orthogonal shell-like curves). During the smooth motion phase, we see a pattern of parallel lines behind the bead, demonstrating that the network does not contract orthogonally as it moves away from the bead surface, which agrees with previous experimental work showing no orthogonal network contraction for motile beads [17],[38]. So in our simulations, orthogonal contraction of the network does not provide the driving force for motility by squeezing the bead forwards. In Figure 5A, the time-pulse markings highlight regions of network that come from the bead surface within short time windows—in effect demonstrating what happens to the equivalent of “shells” for smooth motion. In the tail, they appear as red lines with curvature much lower than the bead curvature, i.e., even during smooth motion, the high-curvature network produced at the bead is opening up just like the shell during symmetry breaking. The shape of these smooth-motion shells also match well those produced by physically switching the color of the actin during in vitro experiments [17],[38].

Even though the bead in this simulations is not constrained, during smooth motion, the network sweeps around the bead primarily in one plane—Figure 5A shows that the tail is much wider in one axis than the other, similar to the shell during symmetry breaking in Figure 2A and 2B. In three dimensions (Figure 5B and Figure S7), tracking the network trajectory shows ripping in one axis along a sustained straight-line crack at the front of the bead. We confirmed that the trajectories of the network in our simulations match those seen in vitro using fluorescent speckle microscopy (Figure 5C, Video S13). The composite image is produced by coloring and overlaying successive frames from a video of a motile bead in vitro, registered to the motile bead (i.e., lines represent movement relative to the bead). The trajectories in vitro mirror those seen in silico, with network expanding away from the bead as it is swept around and incorporated into the tail, and no convergence of trajectories behind the bead. The effect of this sweeping motion on the circumferential tension in the simulated network can be seen in Figure 5D. The network shows a peripheral zone of circumferential tension (red) at the outer network surface, and a region of network compression (blue) just behind the bead. This tension zone is far from the bead surface except at the thinnest part of the network at the front of the bead.

The opening of the “smooth-motion shells” in Figure 5A is reminiscent of how the shell opens during symmetry breaking, and suggests that the network might contract circumferentially and expand radially, as we saw during symmetry breaking in Figure 3. To test this, we made similar measurements of the network stretching during smooth motion, and because the network is asymmetric during smooth motion, we restricted measurements to the rear of the bead; Figure 5E and 5F show lines used to take circumferential and radial length measurements during the smooth motility phase (shown in Videos S14 and S15). Figure 5G shows how the network behind the bead stretches as the bead moves, confirming that it stretches circumferentially to approximately 120% before relaxing back to approximately 107% of its original length. As it does so, it expands radially to approximately 112%—similar to the radial expansion of the outer shell during symmetry breaking. This relaxation is complete after approximately 150 frames (∼18 µm), consistent with previous in vitro photobleaching data showing the network is still undergoing relaxation at approximately one bead diameter and is complete by approximately four bead diameters [17].

Why do the trajectory lines of the network look parallel (and even diverge slightly) as they move away from the bead? Although the network contracts circumferentially, it also rotates around the bead, i.e., the network on the outer edges of the tail sweeps backwards relative to the inner tail. This rotation allows the points in this smooth-motion equivalent of a shell to contract relative to one another while following the parallel trajectories shown in Figure 5B; i.e., there is circumferential, but not orthogonal, network contraction. The Soap-Squeezing model proposes that orthogonal elastic contraction of the network drives motility. The lack of orthogonal network contraction rules this out, but could circumferential elastic network contraction play a similar role?

To determine whether circumferential elastic contraction is required for motility, we performed in silico experiments to find out what happens when elastic contraction is reduced or eliminated. Changes in these parameters affect both the bead velocity profile and the stretching of the shell. Figure 5H shows the velocity profile of the bead described above, before reducing elastic contraction. The bead is initially at rest, with a distinct spike in velocity upon the original symmetry-breaking event. (Note: the smooth motility regime still has small velocity fluctuations, especially just after symmetry breaking. To clearly distinguish between the two regimes, we define smooth motion as having velocity that varies <25% of the mean velocity, and pulsatile motion as having velocity that varies >100% of the mean velocity.)

We first reduced the elastic contraction by tuning network parameters to produce a less elastic network. We based these parameters (*R*_M_ = 5.0, *F*_BL_ = 2.0, *F*_L_ = 4.0) on the Model Robustness results, Section S3 in the supporting text (Protocol S1). Figure 5I shows this less elastic network expands more and contracts less: the network stretches circumferentially to 133% of its original length before relaxing back to only 128%, with a slight radial expansion, to 105%. The velocity profile under these conditions (Figure 5J) shows smooth motility, but strikingly lacks the initial spike in velocity compared to Figure 5H, and the onset of motility is delayed. For the elastic network, the initial velocity spike corresponds to the symmetry-breaking event, and Figure 5M shows that for the less elastic network, rather than producing a single shell with its buildup of elastic energy and sudden release and contraction that ejects the bead, the network fractures in multiple places, producing three separate tails. Eventually, the bead squeezes out orthogonal to these tails (Figure 5N, Videos S16 and S17), with smooth motion and network trajectories that resemble the bead in Figure 5A. In spite of being less elastic, this network still contracts circumferentially, and observation of network motion suggests this contraction is likely driven by network fractures that opened during expansion being closed by the compression forces of material swept around the bead. To abrogate this contraction, we performed the same experiment but allowed network movement only for nodes within a limited range of the bead, permitting the network to expand, but locking it in place before it could contract. This results in similar smooth motility (and a similar pattern of network tracks) under these conditions, showing that network recoil is not required for smooth motion (Figure 5L and 5K, and Video S18).

### Sustained Rip Model for Motility

What explains smooth directional motility? We propose a “Sustained Rip” model: an extension of the symmetry-breaking mechanism combined with a pressure-induced transition from brittle to plastic network behavior. For smooth motility, as during symmetry breaking, network produced at the bead surface tends to be pushed outward, creating circumferential tension (Figure 5D). During motility, however, the existing shell (or tail) reinforces the network at the rear, forcing circumferential tension to be relieved by stretching and ripping at the front (Figure 5B). The radial compression that balances the circumferential tension presses on the bead from all sides except where there is little network—at the front (Figure 5D). The imbalance of these compressive forces causes the bead to move forwards, driving it through the rip site. Ripping also means that radial compression does not build up enough to compress the network and cause it to become dense and brittle—it remains sparse and plastic. Direction is maintained because contact with the tail (or the original shell) always reinforces the network at the back, leaving tension from the expanding network to be relieved by ripping in the unreinforced zone at the front. The network trajectories in Figure 4D and circumferential tension plot in Figure 5D support this, showing that contact with the original shell restricts the new network from free expansion at the rear—the new network does not expand symmetrically as the original shell did in Figure 4A, but diverges less in the rear region in contact with the shell, and more at the front.

This Sustained Rip model predicts that specific changes in network properties will affect the continuity of motion. For example, after symmetry breaking, motility should be smooth only if the newly forming network is sparse and plastic when uncompressed. If the newly forming network has a high enough link density that it behaves like the brittle inner network of the original shell, we should see pulsatile motion—essentially repeated symmetry breaking as new brittle shells form one after another. Changing the probability of forming network links (*P*_XL_) is a simple way to test this prediction by altering the network link density. (Note that this is an alternative to, but does not exclude, friction as a contributor to pulsatile motion [20].)

**Figure 6:**
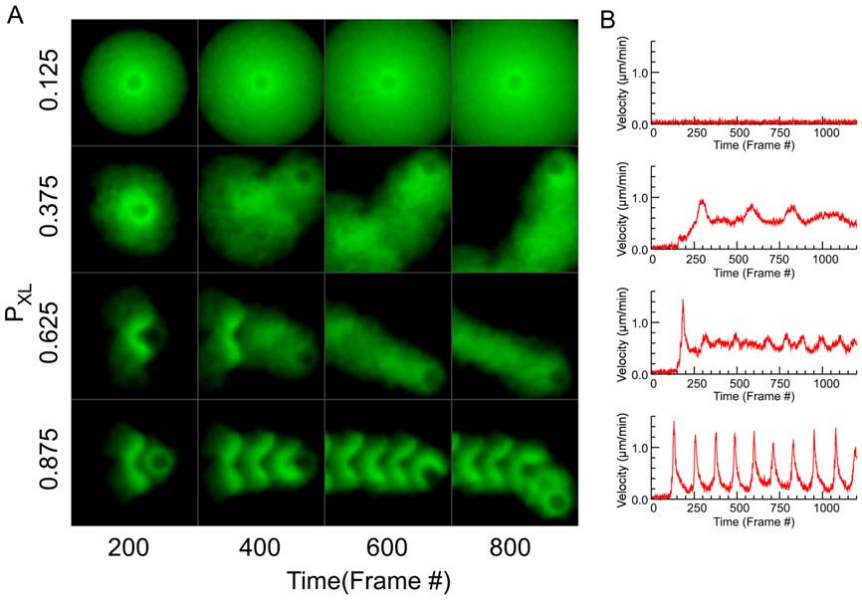
Increasing the degree of network crosslinking (*P*_XL_) causes a transition from smooth to pulsatile motion. (A) Network morphologies and (B) bead velocities over time for values of *P*_XL_ indicated. Very low crosslinking (*P*_XL_ = 0.125) leads to no symmetry breaking. Low crosslinking (*P*_XL_ = 0.375) bead oozes from network cloud. Higher crosslinking (*P*_XL_ = 0.625) gives normal shell symmetry break and smooth motion, and very high crosslinking(*P*_XL_ = 0.875) leads to repeated shell formation and pulsatile motion as the shells break.

We ran simulations to see how varying the probability of forming links affects the smoothness of motility. Figure 6A shows the network architecture at regular time intervals, and Figure 6B shows the corresponding bead velocity profiles, for a range of link probability (*P*_XL_) values. At very low link probabilities (*P*_XL_ = 0.125), there are so few links that each part of the network behaves independently rather than forming a single coherent network—and a symmetric cloud of material surrounds a stationary bead. Increasing *P*_XL_ to 0.375, symmetry breaks and the bead moves off. Under these conditions, the shell is barely coherent—it remains together but does not recoil when symmetry breaks; instead a diffuse cloud of material forms, and the bead gradually oozes from it. There are fluctuations in the velocity, but they remain small (<25% deviation from the mean velocity). As we increase *P*_XL_ to 0.625, a distinct shell forms, the bead undergoes one pulse after the initial symmetry break, and then the motion becomes smooth (<25% deviation from average velocity). As *P*_XL_ increases further to 0.875, the shell becomes denser, and the motion becomes very strongly pulsatile (>250% deviation from the mean velocity) and periodic, as strong shells repeatedly undergo largely independent symmetry-breaking events. Bead velocity rises abruptly when the shell breaks, and tails off slowly as the shell relaxes, leading to an asymmetric velocity profile that closely matches experimental measurements of bead velocity during pulsatile motion [20]. This transition from smooth to pulsatile motion supports the Sustained Rip model for motility: as network coherency increases, the stronger shells formed are more immune to the influence of the previous shell, causing them to undergo essentially independent symmetry breaking. The small influence of the previous tail explains the relatively constant direction of motion.

Further supporting the Sustained Rip model, two other parameters of the APS model also control smoothness of motility by affecting the ability of the old network to alter the brittleness of the newly forming network: 1) Increasing the node repulsive force makes the network less compressible, reducing the pressure-dependent density increase, and leading to smooth motion (Figure S15); and 2) lowering the link spring constant *F*_L_ results in circumferential tension (and radial compression) building up more slowly (i.e., the network has to get bigger before the dense, brittle shell forms) causing a much thicker shell when symmetry breaks, thick enough to be beyond the effect of the initial tail, and immune from the sustained rip effect's ability to induce smooth motion (Figure S14).

Friction may also contribute to pulsatile motion: in vitro, increasing surface ActA concentration (intended to increase the ActA-filament attachment component of friction) causes a transition from smooth to pulsatile motion [20]. We see a similar effect in our simulations: when we increase friction by increasing the strain limit before node–bead links break, we also see a transition from smooth to pulsatile motion (Figure S17; note the transition is less clear-cut than those described above). However, in the APS model, we can show that friction is unnecessary for pulsatile motion. We can set friction to zero by eliminating node-bead links, but still induce the transition from smooth to pulsatile motion by increasing network coherency, e.g., by increasing *P*_XL_ (Figure S20). We interpret this to mean that the change from smooth to pulsatile motion is directly caused by a change from a plastic to brittle network, and that a dense, brittle network can be caused by increasing its density in two ways, either 1) by increasing the coherency of the outer shell, which puts pressure on the inner shell, or 2) by increasing the network–bead attachment, which increases the density of the inner shell by holding it close to the bead surface.

### Capsule (*Listeria*-Like) and Ellipsoidal Geometry

**Figure 7:**
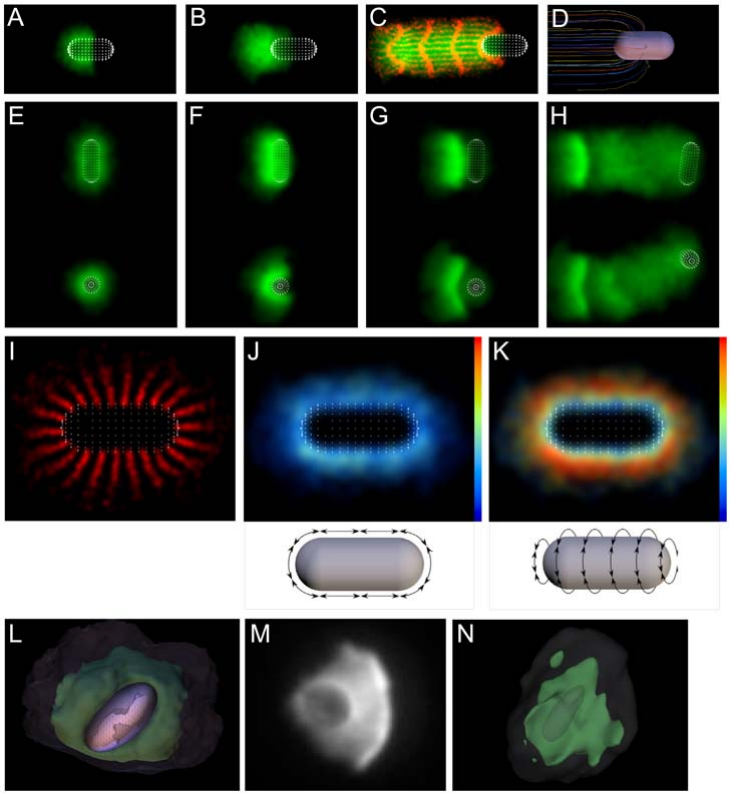
Simulation predicts sideways symmetry breaking and motility for symmetrically coated *Listeria* and ellipsoids. (A–D) Simulation with nucleation localized to only one half shows motion in the direction of the long axis of the *Listeria*. (A–C) Time series during motion. (C) also shows regularly spaced and timed speckle tracks that show trajectory and deformations of the network (see Video S19). (D) 3-D network trajectory showing no orthogonal squeezing (see Figure S8). (E–H) Time series of simulation for uniformly nucleating *Listeria* shows sideways symmetry breaking and motility (see Video S20) (side and top view of same run shown). (I) Network trajectory prior to symmetry breaking shows network being drawn towards poles of the capsule. (J and K) Circumferential link forces around the capsule split into components as shown (plotted to the same scale). Circumferential tension builds up preferentially around the long axis. (L) 3-D view of ellipsoid simulation after symmetry breaking showing sideways motion (see Video S21). Network density shown by isosurfaces: high density (green) and low density (semitransparent). (M) 2-D projection and (N) 3-D reconstruction of an in vitro ellipsoid experiment after symmetry breaking showing sideways symmetry break.

Our data show how an evenly coated spherical bead can be driven on an actin comet tail, but the original observations of this form of motility were on the intracellular motility of the bacterium, *Listeria monocytogenes*, which is a different shape (capsule-shaped rather than spherical) and has an asymmetric distribution of the actin nucleation factor, rather than symmetric. How important is this asymmetric distribution to the lengthwise motility of *Listeria*? To determine the importance of shape and of nucleator distribution on motility, we tested the effect of varying them in silico. When we simulate a capsule-shaped nucleator with nucleation restricted to one half of the capsule, motility is lengthwise and symmetry breaking is unnecessary (Figure 7A–7D). Network tracks with regular spacing and frequency (Figure 7C) and 3-D tracks (Figure 7D, Figure S8, and Video S19) show that the network expands outward from the nucleator, opening up as it moves away from the surface. Similar to the motility of spherical beads, there is no evidence for orthogonal contraction of the network.

When we distribute nucleation uniformly over the capsule surface, however, the direction of motion changes: for both symmetry breaking and motility, the capsule moves sideway, as shown in top and side views in Figure 7E–7H and Video S20. The Elastic Gel model predicts that the higher the surface curvature, the faster the buildup of strain within the network [19]. We therefore anticipated the higher curvature regions at the ends would build up strain faster and that symmetry breaking would occur there (the ends are higher curvature because although the radii are equal, the curvature is 2-D at the ends but only 1D on the linear section). To understand why symmetry breaks sideways, we examined the network motion by plotting network tracks just prior to symmetry breaking (Figure 7I). This shows that as tension builds up, the network on the linear section is drawn towards the ends of the capsule, so relieving the strain and the network tension in this direction remains low (Figure 7J). Around the capsule's cylindrical axis, however, there is no linear section to expand and relieve the strain buildup, so the tension in this direction builds up rapidly (Figure 7K). Symmetry breaking therefore occurs in this direction (causing sideways motion) by a similar mechanism to the spherical beads, and the sideways symmetry breaking and motion of this geometry can be explained by the sustained rip mechanism described above, in which the axis of the rip is defined by the long axis of the capsule.

We checked our prediction of sideways symmetry breaking and motility by stretching spherical beads to make ellipsoids and comparing their in vitro motion with simulations. Figure 7L (Video S21) shows that simulations of ellipsoids produce the same sideways symmetry breaking seen for the capsules (subsequent motion is also sideways like the capsules, Video S22). We performed bead motility experiments as above with a 15.5-µm headspace (i.e., unconstrained), and captured 3-D *z*-stacks of the beads soon after symmetry breaking. Figure 7M and 7N show a 2-D projection and 3-D reconstruction of such an ellipsoidal bead experiment after sideways symmetry breaking, with two density isosurfaces: the green chosen to show the shell, and the semitransparent grey chosen to outline the void space of the ellipsoidal bead to confirm the bead position and orientation. (Note that it is not possible to determine the direction of motion relative to the bead axis from the 2-D projection in Figure 7M alone.) More examples of sideways symmetry breaking of ellipsoidal beads are shown in Figure S9. For ellipsoid aspect ratios >1.75∶1, we almost always see sideways symmetry breaking (98%, *n* = 58) and sideways motion (95%, *n* = 55), though we occasionally see beads changing direction or curved bead paths during the subsequent motion.

## Discussion

In this study, we show that a minimal set of viscoelastic network properties are sufficient to reconstitute actin-based motility in silico. Having gathered data on the behavior of the actin network during in vitro motility experiments and reconstituted this behavior in silico, we explored this in silico system to show how the network properties give rise to the behavior. We also found some novel behaviors, e.g., sideways motion of ellipsoids and shell dents for 3-D symmetry breaking, which we tested by performing more experiments with the in vitro system. Experimentally confirming these novel predictions without having to re-tweak the model suggests that the model is not simply replicating the experimental data fed to it, but has captured the essence of a significant underlying mechanism of actin-based motility.

### The Actin Network as an Elastic Gel

Our simulations build on the Elastic Gel model of symmetry breaking [19],[31], using an Accumulative Particle-Spring (APS) model to capture the mesoscopic viscoelastic properties of actin networks. The APS model represents these properties using a series of nodes and springs that allow us adjust a simple set of viscoelastic network parameters that correspond to mechanical properties of the in vitro network. For example, the repulsive force between nodes (*F*_R_) roughly corresponds to the resistance of the network to compression, and the spring constant (*F*_L_) roughly corresponds to the resistance to tension. The APS model also captures some network behavior as emergent properties. For example, as the network stretches circumferentially, links reorient circumferentially to result in strain hardening, and compression of the inner network by the outer network increases the node and spring density, resulting in the more brittle behavior necessary to produce the symmetry breaking and transition from smooth to pulsatile motion seen in silico and in vitro.

The APS model builds the network from spring-node units that correspond to a particular mesoscopic mechanical behavior of crosslinked actin networks. We know a good deal about the viscoelastic behavior of in vitro actin networks from studies that examine the randomly crosslinked networks produced by mixing crosslinking proteins with stabilized actin filaments. For these networks, crosslinking proteins connect adjacent filaments with one another to form chains with a characteristic mesh size that can resist tension across the sample. The chains of nodes and springs in silico approximate the behavior of these chains of filaments, crosslinks, and friction, to transmit tension around the in silico bead. For Arp2/3-built networks to transmit tension around the bead implies significant friction and entanglement. Activated at the bead surface by ActA, Arp2/3 binds to existing filaments and nucleates new filaments from their sides to form a dendritic branched structure [3],[5]. Because only new filaments are crosslinked, each dendritic tree cannot crosslink to any other, so there can be no encircling chains of filaments and crosslinks around the bead that could carry tension. Circumferential tension would simply be dispersed by separation of these independent dendritic networks were it not for friction and entanglement. The node-spring links in our APS model, therefore, also implicitly represent these friction and entanglement links between dendritic trees, and just as friction and entanglement would be expected to increase with network density and pressure, so the density of node-spring links in the APS model increase with density and pressure. We create links only at the surface when nodes form, to mimic in vitro filament entanglement, which can only occur when filaments polymerize and insert through gaps in the existing network, and this occurs only at the bead surface. We keep the polymerization rate constant in our simulations in spite of changes in protein concentrations and pressures at the bead surface during shell growth, because previous data show the in vitro rate of deposition of actin to remain essentially constant over this period of the reaction (Figure S6 from [16]).

### Symmetry-Breaking 3-D Geometry

In an expanding shell, the actin network continuously stretches as it is displaced outward by assembly of new actin at the surface. The opening of the shell during symmetry breaking is well explained by the basic assumption of the Elastic Gel model that all network layers tend to relax to their equilibrium area, the area of the surface of the bead where they were created. Since this area is the same for all layers, and since connected layers with equal areas and a non-zero thickness would tend to flatten to a plane, the shell tends to flatten towards a plane once symmetry breaks. For most conditions, we do not see a perfectly flat plane, but we do see the shell relax to a flat plane when we increase the link strength. This is because high link strength reduces the number of links that break in the initial outer shell as it is stretched—high link strength means that links only break during the actual symmetry-breaking event. This explains the curvature of the arc of the symmetry-breaking shell: Before symmetry breaking, as the outer shell is stretched, links break irreversibly, expanding the equilibrium area of the outer shell, so the final shell shape is no longer the relaxation of planes of equal equilibrium areas. The larger equilibrium area of the outer network results in a convex shell.

**Figure 8:**
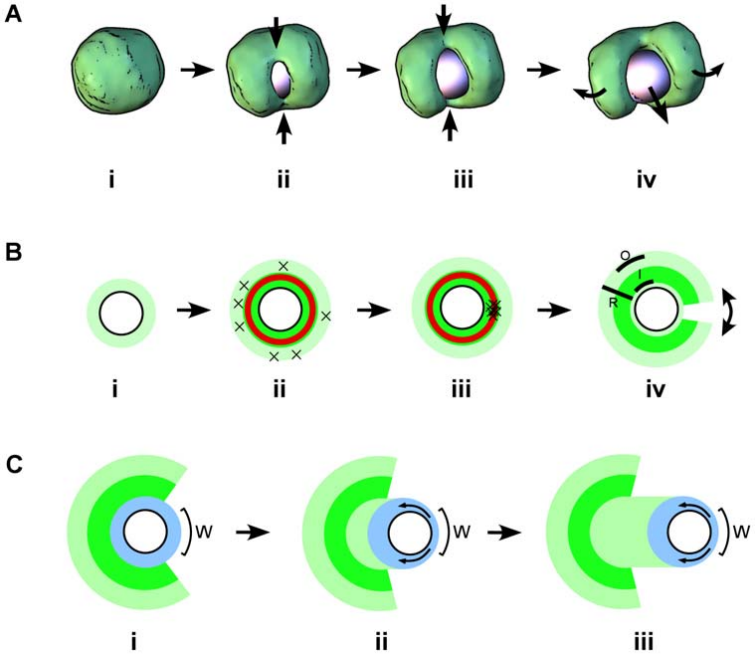
Model for symmetry breaking and motility. (A) 3-D Mechanics of symmetry breaking. (i) The network grows symmetrically until (ii) circumferential tension tears the load-bearing inner network, and a linear crack forms in the shell. The crack propagates through the shell in a straight line at the points of high curvature (arrows). (iii) The crack propagates towards the rear of the shell (arrows), creating a weak point opposite the direction of motion, which acts as a hinge. (iv) The two lobes of the shell open in a plane (curved arrows) about this hinge, allowing the bead to escape. (B) Forces and site selection during symmetry breaking. (i) A loose network polymerizes at the surface of the bead and is pushed radially outward. (ii) Radial expansion causes the outer network to expand and creates circumferential tension, causing random small rips around the outer shell (marked by ×'s). This circumferential tension also compresses the inner network, increasing its density and creating a more rigid, brittle inner shell. Within this inner shell, a spherical shell (slightly away from the bead surface, shown in red) carries most of the circumferential tension. (iii) Circumferential tension is well balanced around this inner shell and continues to build until a small stochastic break occurs, whereupon positive feedback causes catastrophic failure (concentration of rips marked by ×'s) and the linear crack described in (A). (iv) The shell opens, with the outer network (“O”) contracting, the dense inner network (“I”) changing curvature but neither expanding or contracting, and the shell expanding in the radial direction (“R”). (C) Sustained rip model for smooth motility. (i) After symmetry breaking, new network (shown in blue) polymerizes at the surface of the bead. Contact with the original shell reinforces the network at the back, leaving a thinner weaker area of network at the front (“W”). As the new network expands radially, it creates circumferential tension, which rips through the weaker area at the front, and the bead moves forwards. (ii) The existing network at the back continues to reinforce new network (blue), maintaining the weak area (“W”) at the front of the bead. This weak area is sufficiently weak that ripping occurs before enough circumferential tension builds up to reinforce the shell and create a rigid inner region (compare with [B](ii) above) so the network deforms with plastic flow (arrows). (iii) This continues, with the tail rather than the original shell maintaining the rear reinforcement, and the bead moving at steady state (constant velocity) through a sustained rip at the front of the bead.

The APS model also shows how the rip that occurs during symmetry breaking brings about the clam-like 3-D geometry of the shell. Since the starting geometry is a sphere, as the shell opens and flattens, large tensile strains occur around the circumference (Figure 8A). Rips relieve these circumferential strains; a single rip will produce a bilobed structure, but multiple cracks are possible (and observed in silico and in vitro) as the network strength is increased. We also often see a crack in the outer network opposite the main symmetry-breaking crack. When the bead is unconstrained, this tends to line up with the dent in both the simulation (Figure 2A and 2C) and experiment (Figure 2G), but can also be present in constrained beads without the dent (Figure 2E), showing that the dent is not the cause of the rip. In line with a previous experimental observation [35], our simulations also show linear cracks (instead of a round-hole opening to release the bead). These are linear rather than circular because positive feedback concentrates the strain to regions of high curvature [39]. The resulting cracked-shell geometry is reminiscent of the Mollweide projection of the globe, in which linear cuts in the map allow a 3-D sphere to be flattened to a plane and reduce stretching distortions at the poles.

### Compression, Network Coherency, and Mechanism of Motility

Paradoxically, pulsatile motion is relatively simple—it is essentially repeated symmetry breaking—whereas smooth motion is more complex, involving a transition to a different regime. The very same conditions build an initial rigid brittle shell that cleanly and distinctly breaks symmetry and then builds a more plastic tail on which the bead moves smoothly. How does the presence of the old shell cause adjacent new network to behave in the plastic manner that produces smooth motion? Our simulations suggest that this switch to plastic behavior rests on the pressure dependence of network plasticity. By reinforcing one side of the newly forming network, the old shell focuses the circumferential tensile strain on a small region of newly forming, uncompressed, and therefore, plastic network on the other side, which rips. Just like inflating a balloon with duct tape on one side—the duct tape not only prevents that side expanding, but it means the other side is stretched twice as much to accommodate and ruptures sooner. In the bead case, this leads to a rip before pressure has built up—so the network remains sparse and plastic, which in turn leads to continued ripping and steady-state smooth motion. If this pressure dependence is disrupted or reduced, the transition to smooth motion is delayed or abolished. In our simulations, increasing *P*_XL_ increases the number of links and the coherency of the shell, leading to essentially independent shells and pulsatile motion.

We expect this mechanism to correspond to the physical mechanisms that produce the switch to smooth motion seen in real actin networks, in this case through pressure-dependent increases in entanglement, friction, and filament orientation effects (likely to be significantly affected by pressure, as load-directed filaments stall). Oblique filaments would tend to entangle and reinforce the network while contributing little to the movement of the bead away from the network, and so this may tip the system into a positive feedback of network stiffening that is relieved by symmetry breaking. We predict a significant alignment of filaments orthogonal to the direction of motion for a pulsatile bead, but less orthogonal alignment for a smoothly motile bead.

We can also consider these network behaviors in terms of changes in network mesh size. This refers to the distance between the chains of links that transmit tension through the network, i.e., the mesh size decreases as crosslink density increases but is always greater than the individual link lengths. When the symmetry-breaking shell forms, the pressure produces a tightly crosslinked network with a small mesh size (on the order of the link length). Because the mesh size is very much smaller than the bead size and the shell, the network behaves as an elastic solid. Decreasing crosslink density increases the mesh size and results in a mesh size that is larger than the bead, but smaller than the shell. This means that the shell can still resist tension, but beads can essentially move *through* the network, resulting in the oozing symmetry breaking seen in Figures 6 and S11. Decreasing crosslink density still further produces a mesh size greater than the bead and the shell, so tension is not communicated around the bead, and symmetry does not break. The switch from brittle to plastic behavior can also be seen in terms of mesh size. Although the pressure buildup in the initial shell produces a dense network with small mesh size and elastic-solid behavior, once symmetry breaks and the rip at the front prevents pressure buildup, the sparse network at the front of the bead essentially has a large mesh size that allows the bead to move through unhindered.

The repeated shell-breaking mechanism we propose for pulsatile motion does not exclude other proposed models; e.g., *Listeria* and motile vesicles have asymmetric nucleator localization during motility [16],[20],[38],[40]–[42], so are unlikely to build up symmetric shells. This suggests a friction mechanism for pulsatile motion, though pressure buildup still may contribute to periodic variations in friction. In our simulations, we show that a frictionless bead still produces pulsatile motion, suggesting that although friction may contribute to pulsatile motion, it may not be required. In addition to the pulsatile motion whose steps are of the order of the bead size, *Listeria* can also make steps of approximately 5.4 nm [29],[43]. These “nano-saltations” are very likely to be directly caused by friction because their scale is of the order of actin monomers, much smaller than the characteristic scale of the elastic gel properties of the network.

### Site Selection during Symmetry Breaking

Our prediction that the shell outer network is more flexible and plastic and the inner network more rigid and brittle has implications for the mechanism of symmetry breaking. The driving force behind symmetry breaking is the circumferential stretching of the network as it moves outward, and we initially expected to see a brittle crack in one region of the outer network that would seed the symmetry break as has been previously proposed [32],[33]. Instead, we find that the symmetry-breaking direction is determined late because the tensile stress is primarily carried, not by the very outer network, but by a dense rigid network relatively close to the bead surface. We stress that this does not mean that the network does not rip at the outside first—it does because this is the most stretched region—but the outer network rips in many places without triggering symmetry breaking; it is the rip of the inner network that determines the symmetry breaking site, and this is not determined by the outer network.

If stochastic variations in the density of the initial (outer) layers of the network were to determine the symmetry-breaking direction, we would expect the direction to be determined early, when this initial network forms. We show that symmetry-breaking direction is determined late in the simulations, just before the rip occurs, implying that there is no existing vulnerability in the outer network that later seeds the crack, but rather that network density and linking are finely balanced up to the critical point when load becomes too great, and failure occurs stochastically. This fits well with the mechanism proposed above for curved versus flat shells: the balanced stochastic breaking of links in the outer network, not only equilibrates the strain, but results in the even-expansion equilibrium area of the outer shell. When symmetry breaks, shell curvature is determined by the balance of the equilibrium areas of the inner and outer shells—when the outer layer equilibrium area expands, we see curved shells, and when the link strength is increased, the even breaking is eliminated, the outer layer equilibrium area does not expand, and we see flat shells.

Our conclusions about site selection are based on our simulations—so do they also hold for the in vitro system? This depends on where tension is carried, which depends on the network rigidity—if the inner network is more rigid than the outer network in vitro, then our conclusions should hold; if the outer network is more rigid than the inner, then they will not. There are several reasons to think the inner network will be more rigid in vitro: First, the inner network is denser in vitro, as shown in Figures 1 and 3. Second, we often observe numerous small cracks in the outer network (Figure 1A and 1B) prior to symmetry breaking that do not predict symmetry-breaking direction, but rather suggest a general stochastic fracture of the outer network similar to the general breakage of links we observe in the simulations. A third reason follows if the Sustained Rip model is valid, since it predicts that under no compression the network will be plastic, not rigid. Since the initial outer shell is formed under no compression, it should be plastic and therefore not carry significant tension.

### Elastic Recoil and Soap Squeezing

We show that elastic recoil is not required for smooth motility, but is necessary for the classic “shell-retraction” type of symmetry breaking. At first sight, the lack of orthogonal network contraction during bead motility seems to suggest a lack of elastic recoil during smooth motion, but detailed data from our simulations show elastic retraction circumferentially around the bead and, because of its positive Poisson's ratio, radial expansion, an elastic recoil very similar to symmetry breaking. Although elastic recoil is not required for smooth motility, it is necessary for the shell retraction during symmetry breaking. Without it, the network is unable to expand circumferentially and absorb the energy with elastic stretching, but instead quickly rips, resulting in several tails from which the bead eventually emerges.

During smooth motility, the network motion appears dominated by plastic flow around the bead. In previous work, Paluch et al. [17] describe a model for smooth motility that explains the network motion by treating the actin network as an incompressible gel that flows around the bead. Although this model relies on force generation by soap squeezing, which is contradicted by their photobleaching data, the general model of network motion by flow of an incompressible gel is consistent with our findings that network compressibility and retraction are not required for smooth motility. Lacking experimental data, previous models have varied widely in their assumptions about network compressibility [31],[33],[37], though recent work suggests it is a particularly important determinant of stress buildup [44]. In our simulations, the plastic flow we see during smooth motility approximates an incompressible gel regime, not because the gel itself is less compressible, but because the compressive forces are lower—the front rip prevents pressure building up enough to significantly compress the gel. The results of our simulations show how the two processes can be reconciled in one system: Symmetry-breaking behavior is dominated by network compression and elastic recoil because the shell is elastic and brittle because it is built under high pressure, whereas smooth motility is dominated by plastic flow because the tail is built under lower pressure because of tension release at the rip.

Our conclusions also agree with previous results showing that actin shells from which a solid bead escapes open wide, straighten, and then go on expanding after the bead has moved out [38]. In that paper, Delatour et al. [38] also suggest that evacuation of the gel by elastic recoil is required for movement by evacuating the actin filaments grown in front of the bead to maintain anisotropy in the system. This is based on the observation that during pulsatile motion, the bead periodically slows down and reinitiates the formation of a quasisymmetric actin shell and repeats the initial symmetry-breaking step over and over. The actin shells in this regime are never perfectly symmetrical, but weaker at the front, so the initial direction of the movement (defined by the gap in the first shell) is partially conserved. Our results support Delatour et al.'s interpretation that direction is maintained mechanically by reinforcement by the existing tail, but we differ in our interpretation of the role of elastic recoil. We find that elastic recoil is not necessary for movement (though its absence prevents pulsatile motion); rather, plastic flow evacuates material from the front of the bead. In our model, direction is also maintained by the tail, which reinforces the network at the rear of the bead, but this works by concentrating circumferential tension at the unreinforced zone at the front, leading to a sustained rip.

### Capsule (and Ellipsoid) Symmetry Breaking

We find that the Elastic Gel model helps explain the sideways symmetry breaking and motility of capsule-shaped and ellipsoidal nucleators. The network stretches around the long axis to relieve the circumferential tension, so only around the short axis does tension buildup cause symmetry breaking (and motility) in the sideways direction. Our experiments using ellipsoidal beads confirm this behavior in vitro, and support the elastic gel mechanism as the determinant of symmetry breaking and motility behavior.

We show that for lengthwise symmetry breaking and motility, a capsule geometry requires asymmetric nucleation. Wild-type *Listeria* is capsule-shaped, moves lengthwise, and has such an asymmetric distribution of its ActA nucleation factor [45],[46], but a deletion mutation of ActA has been identified that results in a “skidding” sideways motion of *Listeria* in vivo [47]. Our data raise the possibility that the effect of this mutation could be to alter the asymmetric distribution of ActA activity.

### Limitations and Strengths of Modeling

Simple models such as ours have limited scope—e.g., we do not include filament-specific effects such as filament orientations and elongation by monomer addition—so we cannot evaluate the Brownian ratchet mechanism, nor can we investigate the hollow tails seen for beads coated with VASP [27], or recreate the nano-saltations observed in vitro [43]. The first 3-D computer simulation of actin-based *Listeria* motility took a detailed approach, simulating the behavior of large numbers of individual actin filaments and branches [29]. The Alberts-Odell model provided an important insight into the connection between the microscale behavior of individual filaments and larger-scale behavior of motile networks, namely how the buildup and breakage of filament-load attachments can produce nano-saltations in motility similar to those observed experimentally [43]. As with our model, the Alberts-Odell model has limited scope. To make their model computationally tractable, Alberts and Odell modeled actin filaments as inflexible rods, fixed rigidly in space soon after nucleation. Thus, the actin network in their model is an inelastic solid and could not be used to study processes involving elastic energy storage, plastic deformation, or mechanical failure: e.g., the Alberts-Odell model could not be used to study mechanical symmetry breaking or the role of elastic recoil in sustained motility. Concentrating on different aspects of the system, the two models complement one another and explain a wider range of behaviors.

Our approach has been to use a simple model with few parameters that confers strong explanatory power at the risk of oversimplifying the physical mechanisms. One potential oversimplification in our model is the constancy of conditions: e.g., we assume no changes in polymerization rate over time or spatially over the bead surface. The concentrations of components change during the reaction, and although this does not affect the rate of actin polymerization in the shell in vitro [16], this does not mean it does not affect more subtle physical characteristics of the network architecture. We also know that Arp2/3-based actin nucleation is autocatalytic [48], which might bias polymerization to the rear of the bead where there is a higher density of existing actin and help maintain directional motion. Our simulations include the code to implement such processes, but we have deliberately not used them in the current study (Ockham's razor). This allows us to show that we can explain the behavior of the system using viscoelastic mechanical effects alone.

The goal of this simulation has been to demonstrate the qualitative mechanisms of symmetry breaking and motility, and we have stressed that our simulations do not produce calibrated physical quantities for force, speed, etc. To do so would require both kinetically tuning the model to a more extensive experimental dataset, and also to include a more sophisticated treatment of internal network friction. The current model treats drag very simply: the system is over-damped, with drag proportional to velocity relative to the reference frame, consistent with a low Reynolds number regime. This explains a significant deviation between our model and our experimental data: that the rapid recoil of the shell in symmetry breaking is slower in our simulations. Would kinetic tuning significantly alter the qualitative behavior of the model? There are two reasons to think not. First, most of the kinetics are close to observed (e.g., the ratio of polymerization rates to rates of shell buildup, relaxation, bead movement, etc. are similar), so adjustments should not be major, and therefore, would be unlikely to affect the qualitative behavior. Second, even during the rapid recoil of the shell when the kinetics are dissimilar, the equilibrium states match well—i.e., the close match in the shapes of the curves shown in Figure 3C and 3E suggest that both the in vitro and in silico systems are relaxing from the same initial to same final states, and therefore, are driven by the same processes.

We have aimed to include as few parameters as possible, and although we make no claims that these parameters correspond to calibrated physical units of the in vitro network, an important question is how their values are chosen and how critical these choices are to the behavior. Essentially, we arrived at values that qualitatively reproduce the behaviors of the in vitro system by systematically exploring the effects of varying the model parameters, e.g., in Model Robustness, Section S3 of the supporting text (Protocol S1). Some behaviors (e.g., symmetry breaking, directional motion) are extremely robust, whereas others, such as smooth motion, are fragile and are disrupted by varying many different parameters.

Working with simulations allows us to refine the hypotheses. Full access to the behavior of the in silico system allows us separate out the gross morphological changes measured in vitro, e.g., the 2-D shape of the final shell, from the underlying components of the motion, e.g., circumferential squeezing, but no orthogonal squeezing, to refine our ideas about the underlying mechanisms. Furthermore, simulations allow us to directly test whether the proposed mechanisms are required for the motion or are epiphenomena, for example, by producing networks in silico that do not have elastic recoil effects and seeing that motion is essentially unchanged.

## Conclusions

The APS model demonstrates how the simple viscoelastic properties of the in silico reconstituted actin gel can give rise to the observed dynamics of symmetry breaking and steady and pulsatile motility of spherical, capsule-shaped, and ellipsoidal objects coated with actin-nucleation factors. The model demonstrates both explanatory and predictive power in these areas, e.g., explaining how a pressure-dependent change in gel properties allows for a transition between motility regimes and predicting the 3-D geometry of in vitro shells.

In the future, we plan to refine the model, calibrating it with time, length, and force data to allow quantitative estimates of internal actin network parameters that are not directly measureable. For example, excising a cubic “slab” of a calibrated nodes-and-link network, then performing “computer experiments” by compressing, stretching, and shearing this slab in silico and recording the resulting stresses will allow us to compute the effective macroscopic elastic moduli of the in silico network, including Young modulus and Poisson ratio. More experimental data will also allow refinement of the functional forms of the repulsive and link forces, and to determine the extent that polymerization is regulated by force.

The APS model also offers a general framework to help investigate other physical cell phenomena that may be dominated by similar, relatively simple viscoelastic behaviors, e.g., lamellipodia and pseudopodia extension and cell septation, by including the effects of interactions with cell membranes, and simulating the anisotropic networks and contractile proteins found in vivo.

## Materials and Methods

### Computational Model

A brief overview of the model is given here (more details are available in the supporting text (Protocol S1) Sections S1 and S4, S5, S6, S7, S8). We simulate the network using a discrete-element approach, i.e., the actin network is represented as network of nodes in 3-D space held together by links (Figures S1 and S2). This is unlike a finite element approach in which the mesh is a way to reduce the dimensionality of a continuum problem into finite number of equations (elements). Rather, network links and the effective mesh size that results are important properties of the network. Network links also have no direct correspondence to actin filaments, but rather the bulk viscoelastic properties of the network of links and nodes are intended to capture the bulk viscoelastic network properties of the actin network. Under the polymerization conditions used (i.e., in the absence of crosslinking proteins) nodes more properly correspond to entanglement of filaments, and links correspond to the elastic properties of the network. We model these links as simple linear springs with a defined breaking strain and an inverse square repulsive force between nodes that models the compression resistance of the material. We explicitly avoid the unresolved question of how polymerizing filaments behave on a molecular level at the nucleator surface (according to Brownian ratchet or other models [24],[49]), and model polymerization as the stochastic introduction of material (nodes) at constant rate at the nucleator surface. Simulations begin at *t* = 0 with zero nodes (and links). Once introduced, new nodes form links with their neighbors, with a higher probability of forming links with nearby nodes (linear tail-off with distance, max probability *P*_XL_ at zero distance), and a limit on the maximum number of links. Nodes at the surface of the bead are also linked to the bead at their last contact point by a link with force proportional to its length. Forces are calculated iteratively (Figures S3 and S21), and since this is a low Reynolds number regime, there is no inertia (i.e., velocity is proportional to force.)

### Computational Details

The computational model is implemented in C++, and run times to symmetry breaking are approximately 1–2 h on a typical desktop computer. The code is designed to use multiple threads to enable large-scale problems to be explored across a number of parameter regimes (runs typically involve 10^5^ nodes, 10^6^ links, and 10^6^ iterations per simulation). The code is open source and made freely available under the GNU General Public License to allow the results to be reproduced, to convey the full details of the model, and to encourage further use of the code by other researchers. A snapshot of the source code together with the parameter control file (Protocol S2) and a compiled executable for Mac OS X (Protocol S3) are provided. A detailed explanation of the code and the parameter control file are included in the supporting text (Protocol S1) and in an online wiki at http://www.dayel.com/comet, where the latest version of the code can also be downloaded.

### In Silico Visualization and Measurements

To visualize the results of the simulations in a way comparable to in vitro microscopy images, we calculate the symmetry breaking plane, and create a 2-D projection of the nodes of the network convolved with a Gaussian to represent the point spread function of the microscope. To make visual comparison easier, we rotate the reference frame afterwards so that the bead always appears to move to the right. Measurements of forces in the radial and circumferential directions in Figure 4 are calculated as components in the direction of, or perpendicular to, a vector from the bead center, the magnitudes of which are summed over spherical shells of different radii. “Stretch factor” measures in Figures 4 and 5 are calculated by measuring the distance between particular pairs of nodes over time, normalized to the initial distance then averaged.

### Bead Motility Experiments

Bead motility experiments were carried out as previously described [16], with modifications. Briefly, 5-µm diameter carboxylated polystyrene beads (Bangs Laboratories) were covalently coated with ActA. The motility mix contained 0.5 mM ATP, 1 mM MgCl_2_, 1 mM EGTA, 15 mM TCEP-HCl, 50 mM KOH (to neutralize TCEP-HCl), 20 mM HEPES (pH 7.0), 125 nM Arp2/3 complex, 100 or 120 nM capping protein, and 3 µM actin. To aid microscopic observation, we included 3 mg/ml BSA (A0281; Sigma-Aldrich) and 0.2% methylcellulose (M0262; Sigma-Aldrich). Initial attempts to define headspace by controlling reaction volume were unsuccessful—the coverslip was not perfectly parallel to the slide, causing the headspace to vary across the sample—so we controlled the headspace by adding 0.1% v/v 5.1-µm or 15.5-µm diameter glass spacer beads (Duke Scientific) prior to starting the reaction. For 3-D reconstructions, reactions were stopped before imaging by adding 50% volume of 15 µM phalloidin and 15 µM Latrunculin B (Sigma-Aldrich). Fluorescent speckle microscopy (Figure 3A) conditions: 7.5 µM actin (1/3,000 TMR-labeled), 3 µM profilin, 40 nM Arp2/3, and 56 nM capping protein.

For the ellipsoidal bead experiments, spherical beads were stretched as previously described [50] with the following modifications: 140 µl of polystyrene bead stock was suspended in 6 ml of 3.8% w/v suspension of polyvinyl alcohol (PVA). The PVA/bead suspension was degassed before casting films in a 4.5×7.0 cm leveled tray. After stretching, the PVA was dissolved by incubating at 90°C for 2 h in distilled water containing 0.1% NP-40. The beads were washed three times in isopropanol and dried in a rotary evaporator. The bead surface was refunctionalized by incubation in 50% (w/v) NaOH for 1 h at 90°C and overnight at 42°C, washed once with 20 mM Tris HCl (pH 8.0) and 0.1% NP-40, and three times with 0.1% NP-40 before coating with ActA.

## Supporting Information

Figure S1 **Diagram of network and forces acting on nodes.**
(0.04 MB PDF)

Figure S2 **Cross-section of network showing links around bead.** The bead would be in the lower left, not plotted so as not to obscure the links.
(0.32 MB JPG)

Figure S3 **Basic form of the main program loop.**
(0.03 MB PDF)

Figure S4 **Interactive 3-D reconstructions of in silico shells from unconstrained (top) and constrained (bottom) beads showing linear crack or bilobed structure.** Beads are 5-µm diameter. For the constrained condition, head space between slide and coverslip is controlled with 5.1-µm diameter glass spacer beads mixed into the reaction. For the unconstrained, 15.5-µm diameter glass spacer beads were used.
(0.23 MB PDF)

Figure S5 **2-D projections (left) and corresponding interactive 3-D reconstructions (right) of constrained beads (5 µm spacers) showing smooth opening of shell without bilobed structure.** Beads are 5-µm diameter. Head space between slide and coverslip is controlled with 5.1-µm diameter glass spacer beads mixed into the reaction. The 2-D projections are the confocal *z*-stacks summed in the *z*-direction. The 3-D reconstructions are isosurfaces at low density (transparent) and high density (green), thresholds chosen to best convey the shell morphology.
(0.80 MB PDF)

Figure S6 **2-D projections (left) and corresponding interactive 3-D reconstructions (right) of unconstrained beads (15-µm spacers) showing bilobed and trilobed structure.** Beads are 5-µm diameter. Head space between slide and coverslip is controlled with 15.5-µm diameter glass spacer beads mixed into the reaction. The 2-D projections are the confocal *z*-stacks summed in the *z*-direction. The 3-D reconstructions are isosurfaces at low density (transparent) and high density (green), thresholds chosen to best convey the shell morphology.
(0.49 MB PDF)

Figure S7 **Interactive 3-D view of in silico network trajectory relative to bead during smooth motion.** Network trajectory lines represent motion of an evenly distributed subset of nodes relative to the bead.
(0.20 MB PDF)

Figure S8 **Interactive 3-D view of in silico network trajectory relative to half-coated capsule during smooth motion.** Network trajectory lines represent motion of an evenly distributed subset of nodes relative to the capsule.
(0.15 MB PDF)

Figure S9 **2-D projections (left) and corresponding interactive 3-D reconstructions (right) of shells and tails from unconstrained ellipsoidal beads showing sideways symmetry breaking and motility.** The 2-D projections are the confocal z-stacks summed in the *z*-direction. The 3-D reconstructions are isosurfaces at low density (transparent) and high density (green), thresholds chosen to best convey the shell morphology and void space to show bead orientation.
(0.33 MB PDF)

Figure S10 **Effect of varying RADIUS.** Matrix plot showing 2-D projection of simulation at time points indicated for a range of RADIUS parameter values. Corresponding bead velocity profiles are plotted on the right. The basis parameters are shown in the top left (zoom to view).
(0.46 MB JPG)

Figure S11 **Effect of varying P_XLINK.** Matrix plot showing 2-D projection of simulation at time points indicated for a range of P_XLINK parameter values. Corresponding bead velocity profiles are plotted on the right. The basis parameters are shown in the top left (zoom to view).
(0.47 MB JPG)

Figure S12 **Effect of varying P_NUC.** Matrix plot showing 2-D projection of simulation at time points indicated for a range of P_NUC parameter values. Corresponding bead velocity profiles are plotted on the right. The basis parameters are shown in the top left (zoom to view).
(0.44 MB JPG)

Figure S13 **Effect of varying LINK_BREAKAGE_FORCE.** Matrix plot showing 2-D projection of simulation at time points indicated for a range of LINK_BREAKAGE_FORCE parameter values. Corresponding bead velocity profiles are plotted on the right. The basis parameters are shown in the top left (zoom to view).
(0.44 MB JPG)

Figure S14 **Effect of varying LINK_FORCE.** Matrix plot showing 2-D projection of simulation at time points indicated for a range of LINK_FORCE parameter values. Corresponding bead velocity profiles are plotted on the right. The basis parameters are shown in the top left (zoom to view).
(0.44 MB JPG)

Figure S15 **Effect of varying NODE_REPULSIVE_MAG.** Matrix plot showing 2-D projection of simulation at time points indicated for a range of NODE_REPULSIVE_MAG parameter values. Corresponding bead velocity profiles are plotted on the right. The basis parameters are shown in the top left (zoom to view).
(0.45 MB JPG)

Figure S16 **Effect of varying NUC_LINK_FORCE.** Matrix plot showing 2-D projection of simulation at time points indicated for a range of NUC_LINK_FORCE parameter values. Corresponding bead velocity profiles are plotted on the right. The basis parameters are shown in the top left (zoom to view).
(0.44 MB JPG)

Figure S17 **Effect of varying NUC_LINK_BREAKAGE_DIST.** Matrix plot showing 2-D projection of simulation at time points indicated for a range of NUC_LINK_BREAKAGE_DIST parameter values. Corresponding bead velocity profiles are plotted on the right. The basis parameters are shown in the top left (zoom to view).
(0.46 MB JPG)

Figure S18 **Effect of varying NUCLEATOR_INERTIA.** Matrix plot showing 2-D projection of simulation at time points indicated for a range of NUCLEATOR_INERTIA parameter values. Corresponding bead velocity profiles are plotted on the right. The basis parameters are shown in the top left (zoom to view).
(0.45 MB JPG)

Figure S19 **Effect of varying FORCE_SCALE_FACT.** Matrix plot showing 2-D projection of simulation at time points indicated for a range of FORCE_SCALE_FACT parameter values. Corresponding bead velocity profiles are plotted on the right. The basis parameters are shown in the top left (zoom to view).
(0.46 MB JPG)

Figure S20 **Effect of varying P_XLINK with no bead-network friction.** Matrix plot showing 2-D projection of simulation at time points indicated for a range of P_XLINK parameter values. Corresponding bead velocity profiles are plotted on the right. The basis parameters are shown in the top left (zoom to view).
(0.47 MB JPG)

Figure S21 **Detailed program flow.**
(0.07 MB PDF)

Protocol S1 **Supporting text.** (SupportingText.pdf)
(7.62 MB PDF)

Protocol S2 **Source code and parameter control file (under GPL open source license). **(comet_src_v0.2.zip)
(0.34 MB ZIP)

Protocol S3 **Mac OS X executable (under GPL/BSD open source license). **(comet_osx_binary_v0.2.dmg)
(6.23 MB ZIP)

Table S1 **Model assumptions based directly on experimental data.**
(0.02 MB XLS)

Table S2 **Model assumptions inferred from experimental data or physical assumptions.**
(0.02 MB XLS)

Table S3 **Corresponding simulation parameter names in the main text and in the code.**
(0.04 MB DOC)

Video S1 **In vitro symmetry breaking and motility for bead uniformly coated with ActA.** See Materials and Methods for conditions.
(1.45 MB MOV)

Video S2 **Computer simulation of symmetry breaking and motility.** 2-D projections of nodes are convolved with Gaussian. Projection plane is chosen parallel to the plane of shell opening.
(1.29 MB MOV)

Video S3 **3-D view of simulation showing links colored by tensile stress.** Color bar range represents zero to breakage stress.
(10.29 MB MOV)

Video S4 **3-D view of simulation in Video S2, viewed from front of bead.**
(10.32 MB MOV)

Video S5 **Fluorescence speckle microscopy video of in vitro symmetry breaking.**
(0.23 MB MOV)

Video S6 **Shell deformations during symmetry breaking (circumferential).** Video showing example point pairs used to measure circumferential shell deformation during symmetry breaking (c.f. Video S7 for radial direction).
(1.66 MB MOV)

Video S7 **Shell deformations during symmetry breaking (radial).** Video showing example point pairs used to measure radial shell deformation during symmetry breaking (c.f. Video S6 for circumferential direction). Point pairs spanning the crack were excluded.
(2.48 MB MOV)

Video S8 **Strain buildup and release by link breakage.** (i) node tracks, (ii) link breaks, (iii) circumferential tension, and (iv) graphs showing how circumferential tension, radial tension, and link breaks vary with distance from the surface of the bead. For link breaks in (ii), color scale bar represents increasing density to the right (red). For circumferential tension in (iii), scale bar represents increasing tension to the right (red) with the black notch representing zero, and the left representing negative tension (i.e., compression) in blue (see Figure 4, and c.f. Video S9).
(3.35 MB MOV)

Video S9 **Strain buildup and release by link breakage, showing compressive and tensile components.** Same as Video S8, but graph shows forces split into compressive and tensile components.
(3.33 MB MOV)

Video S10 **Strain buildup and release by link breakage, showing the circumferential repulsion at the bead surface is distorted due to surface artifact.** Same as Video S9, but including the point closest to bead. The circumferential tension at the bead surface is distorted, a result of the way the code deals with the bead surface - ejecting nodes that enter the bead back to the surface after each iteration. The ability of the network to equilibrate forces requires the nodes to move freely over one another, but this ejection forces the ejected nodes to align at one radius, increasing the number within one shell, and increasing compression in the circumferential direction at the surface. The artifact clearly occurs in only the circumferential repulsion force, and at only one point, closest to the surface.
(4.09 MB MOV)

Video S11 **Link breaks during symmetry breaking shown in 3 orthogonal views.** *x*, *y*, and *z* views of symmetry breaking showing network (grey) and link break density (color scale bar as for Figure 4(ii)). Link breaks are initially stochastic and evenly distributed in the outer shell. Symmetry-breaking rupture of the inner shell is primarily a straight-line crack.
(0.78 MB MOV)

Video S12 **Link breaks during smooth motility localize to outer network towards front of bead.** Symmetry breaking and motility showing network (grey) and link break density (color scale bar as for Figure 4(ii)). Link breaks localize to the front of the bead and outer regions of the network (c.f. Figure 5D).
(5.75 MB MOV)

Video S13 **Fluorescent speckle microscopy video of in vitro smooth motility.** Fluorescence speckles show trajectory of network. (These data were used to generate Figure 5C). See Materials and Methods for more details.
(6.54 MB MOV)

Video S14 **Network deformations during smooth motion (circumferential).** Video showing example point pairs used to measure circumferential tail deformation during smooth motility (c.f. Video S15 for radial direction). Note, for the statistics, only lines that are within the tail were included (circumferential lines spanning the rip were excluded).
(1.42 MB MOV)

Video S15 **Network deformations during smooth motion (radial).** Video showing example point pairs used to measure circumferential tail deformation during smooth motility (c.f. Video S14 for circumferential direction). Note, for the statistics, only lines that are within the tail were included (radial lines with one point at the front of the bead were excluded).
(1.69 MB MOV)

Video S16 **Symmetry breaking and motility for less elastic network.** 2-D projection (*x*-view) showing network breaks into three tails that are pushed outward. Eventually, the bead moves off in the direction of the camera. (Videos S16 and S17 show orthogonal views of same run.) Parameters are defaults except: *R*_M_ = 5.0 pN, *F*_BL_ = 2.0 pN, and *F*_L_ = 4.0 pN (units are nominal).
(0.89 MB MOV)

Video S17 **Symmetry breaking and motility for less elastic network.** 2-D projection (y-view) showing network breaks into three tails that are pushed outward. Eventually, the bead moves off to the right. (Videos S16 and S17 show orthogonal views of the same run). Parameters are defaults except: *R*_M_ = 5.0 pN, *F*_BL_ = 2.0 pN, and *F*_L_ = 4.0 pN (units are nominal)
(2.12 MB MOV)

Video S18 **Symmetry breaking and motility for less elastic network with network contraction prevented.** 2-D projection showing network breaks into two tails that are pushed outward. Eventually, the bead moves off to the right. Parameters are defaults except: *R*_M_ = 5.0 pN, *F*_BL_ = 2.0 pN, and *F*_L_ = 4.0 pN (units are nominal). Nodes are fixed in place when they reach 2× radius from bead surface.
(3.48 MB MOV)

Video S19 **Simulation of half-coated capsule-shaped bead moves lengthwise.** 2-D projection showing network trajectory (marked red at even intervals of time and position around the capsule). There is no orthogonal contraction of network behind the capsule.
(0.87 MB MOV)

Video S20 **Simulation of evenly coated capsule-shaped bead breaking symmetry sideways.** Video shows simultaneous *x* and *y* 2-D projections of network.
(1.29 MB MOV)

Video S21 **Simulation of evenly coated ellipsoidal bead breaking symmetry sideways (3-D view).** Network density shown by isosufraces: high density (green) and low density (semitransparent).
(3.30 MB MOV)

Video S22 **Simulation of evenly coated ellipsoidal bead breaking symmetry and moves sideways (2-D view).** 2-D projection of network. Position of ellipsoid is shown by cage of dots.
(3.25 MB MOV)

## Abbreviations

APS: Accumulative Particle-Spring

## References

[1] Pollard T. D, Earnshaw W. C, Lippincott-Schwartz J (2008). Cell biology.

[2] Chhabra E. S, Higgs H. N (2007). The many faces of actin: matching assembly factors with cellular structures.. Nat Cell Biol.

[3] Mullins R. D, Heuser J. A, Pollard T. D (1998). The interaction of Arp2/3 complex with actin: nucleation, high affinity pointed end capping, and formation of branching networks of filaments.. Proc Natl Acad Sci U S A.

[4] Svitkina T. M, Borisy G. G (1999). Arp2/3 complex and actin depolymerizing factor/cofilin in dendritic organization and treadmilling of actin filament array in lamellipodia.. J Cell Biol.

[5] Nakamura F, Osborn E, Janmey P. A, Stossel T. P (2002). Comparison of filamin A-induced cross-linking and Arp2/3 complex-mediated branching on the mechanics of actin filaments.. J Biol Chem.

[6] Iwasa J. H, Mullins R. D (2007). Spatial and temporal relationships between actin-filament nucleation, capping, and disassembly.. Curr Biol.

[7] Merrifield C. J, Moss S. E, Ballestrem C, Imhof B. A, Giese G (1999). Endocytic vesicles move at the tips of actin tails in cultured mast cells.. Nat Cell Biol.

[8] Taunton J, Rowning B. A, Coughlin M. L, Wu M, Moon R. T (2000). Actin-dependent propulsion of endosomes and lysosomes by recruitment of N-WASP.. J Cell Biol.

[9] Gouin E, Welch M. D, Cossart P (2005). Actin-based motility of intracellular pathogens.. Curr Opin Microbiol.

[10] Frischknecht F, Moreau V, Rottger S, Gonfloni S, Reckmann I (1999). Actin-based motility of vaccinia virus mimics receptor tyrosine kinase signalling.. Nature.

[11] Tilney L. G, Portnoy D. A (1989). Actin filaments and the growth, movement, and spread of the intracellular bacterial parasite, Listeria monocytogenes.. J Cell Biol.

[12] Bear J. E, Svitkina T. M, Krause M, Schafer D. A, Loureiro J. J (2002). Antagonism between Ena/VASP proteins and actin filament capping regulates fibroblast motility.. Cell.

[13] Loisel T. P, Boujemaa R, Pantaloni D, Carlier M. F (1999). Reconstitution of actin-based motility of Listeria and Shigella using pure proteins.. Nature.

[14] Wiesner S, Helfer E, Didry D, Ducouret G, Lafuma F (2003). A biomimetic motility assay provides insight into the mechanism of actin-based motility.. J Cell Biol.

[15] Upadhyaya A, van Oudenaarden A (2003). Biomimetic systems for studying actin-based motility.. Curr Biol.

[16] Akin O, Mullins R. D (2008). Capping protein increases the rate of actin-based motility by promoting filament nucleation by the Arp2/3 complex.. Cell.

[17] Paluch E, van der Gucht J, Joanny J. F, Sykes C (2006). Deformations in actin comets from rocketing beads.. Biophys J.

[18] Cameron L. A, Footer M. J, van Oudenaarden A, Theriot J. A (1999). Motility of ActA protein-coated microspheres driven by actin polymerization.. Proc Natl Acad Sci U S A.

[19] Bernheim-Groswasser A, Wiesner S, Golsteyn R. M, Carlier M. F, Sykes C (2002). The dynamics of actin-based motility depend on surface parameters.. Nature.

[20] Bernheim-Groswasser A, Prost J, Sykes C (2005). Mechanism of actin-based motility: a dynamic state diagram.. Biophys J.

[21] Tilney L. G, Connelly P. S, Portnoy D. A (1990). Actin filament nucleation by the bacterial pathogen, Listeria monocytogenes.. J Cell Biol.

[22] Nakagawa H, Miki H, Ito M, Ohashi K, Takenawa T (2001). N-WASP, WAVE and Mena play different roles in the organization of actin cytoskeleton in lamellipodia.. J Cell Sci.

[23] Peskin C. S, Odell G. M, Oster G. F (1993). Cellular motions and thermal fluctuations: the Brownian ratchet.. Biophys J.

[24] Mogilner A, Oster G (1996). Cell motility driven by actin polymerization.. Biophys J.

[25] Dickinson R. B, Purich D. L (2002). Clamped-filament elongation model for actin-based motors.. Biophys J.

[26] Italiano J. E, Roberts T. M, Stewart M, Fontana C. A (1996). Reconstitution in vitro of the motile apparatus from the amoeboid sperm of Ascaris shows that filament assembly and bundling move membranes.. Cell.

[27] Plastino J, Olivier S, Sykes C (2004). Actin filaments align into hollow comets for rapid VASP-mediated propulsion.. Curr Biol.

[28] Mogilner A (2006). On the edge: modeling protrusion.. Curr Opin Cell Biol.

[29] Alberts J. B, Odell G. M (2004). In silico reconstitution of Listeria propulsion exhibits nano-saltation.. PLoS Biol.

[30] van Oudenaarden A, Theriot J. A (1999). Cooperative symmetry-breaking by actin polymerization in a model for cell motility.. Nat Cell Biol.

[31] Gerbal F, Chaikin P, Rabin Y, Prost J (2000). An elastic analysis of Listeria monocytogenes propulsion.. Biophys J.

[32] John K, Peyla P, Kassner K, Prost J, Misbah C (2008). Nonlinear study of symmetry breaking in actin gels: implications for cellular motility.. Phys Rev Lett.

[33] Sekimoto K, Prost J, Julicher F, Boukellal H, Bernheim-Grosswasser A (2004). Role of tensile stress in actin gels and a symmetry-breaking instability.. Eur Phys J E Soft Matter.

[34] Cameron L. A, Robbins J. R, Footer M. J, Theriot J. A (2004). Biophysical parameters influence actin-based movement, trajectory, and initiation in a cell-free system.. Mol Biol Cell.

[35] van der Gucht J, Paluch E, Plastino J, Sykes C (2005). Stress release drives symmetry breaking for actin-based movement.. Proc Natl Acad Sci U S A.

[36] Waterman-Storer C. M, Desai A, Bulinski J. C, Salmon E. D (1998). Fluorescent speckle microscopy, a method to visualize the dynamics of protein assemblies in living cells.. Curr Biol.

[37] Noireaux V, Golsteyn R. M, Friederich E, Prost J, Antony C (2000). Growing an actin gel on spherical surfaces.. Biophys J.

[38] Delatour V, Shekhar S, Reymann A-C, Didry D, Le K. H. D (2008). Actin-based propulsion of functionalized hard versus fluid spherical objects.. New J Phys.

[39] Anderson T. L (2005). Fracture mechanics: fundamentals and applications.

[40] Smith G. A, Portnoy D. A, Theriot J. A (1995). Asymmetric distribution of the Listeria monocytogenes ActA protein is required and sufficient to direct actin-based motility.. Mol Microbiol.

[41] Lasa I, Gouin E, Goethals M, Vancompernolle K, David V (1997). Identification of two regions in the N-terminal domain of ActA involved in the actin comet tail formation by Listeria monocytogenes.. EMBO J.

[42] Boukellal H, Campas O, Joanny J. F, Prost J, Sykes C (2004). Soft Listeria: actin-based propulsion of liquid drops.. Phys Rev E Stat Nonlin Soft Matter Phys.

[43] Kuo S. C, McGrath J. L (2000). Steps and fluctuations of Listeria monocytogenes during actin-based motility.. Nature.

[44] Dafalias Y. F, Pitouras Z (2009). Stress field in actin gel growing on spherical substrate.. Biomech Model Mechanobiol.

[45] Rafelski S. M, Theriot J. A (2006). Mechanism of polarization of Listeria monocytogenes surface protein ActA.. Mol Microbiol.

[46] Niebuhr K, Chakraborty T, Rohde M, Gazlig T, Jansen B (1993). Localization of the ActA polypeptide of Listeria monocytogenes in infected tissue culture cell lines: ActA is not associated with actin “comets”.. Infect Immun.

[47] Lauer P, Chow M. Y, Loessner M. J, Portnoy D. A, Calendar R (2002). Construction, characterization, and use of two Listeria monocytogenes site-specific phage integration vectors.. J Bacteriol.

[48] Machesky L. M, Mullins R. D, Higgs H. N, Kaiser D. A, Blanchoin L (1999). Scar, a WASp-related protein, activates nucleation of actin filaments by the Arp2/3 complex.. Proc Natl Acad Sci U S A.

[49] Mogilner A, Edelstein-Keshet L (2002). Regulation of actin dynamics in rapidly moving cells: a quantitative analysis.. Biophys J.

[50] Ho C. C, Keller A, Odell J. A, Ottewill R. H (1993). Preparation of monodisperse ellipsoidal polystyrene particles.. Colloid Polym Sci.

