# Supplementary material for: In Silico Reconstitution of Actin-Based Symmetry Breaking and Motility
Source: PLoS Biol. 2009 Sep 22;7(9):e1000201. doi: 10.1371/journal.pbio.1000201 (PMC2738636; doi:10.1371/journal.pbio.1000201)
Supplement: Figure S1 — Diagram of network and forces acting on nodes. (0.04 MB PDF) [file pbio.1000201.s001.pdf]

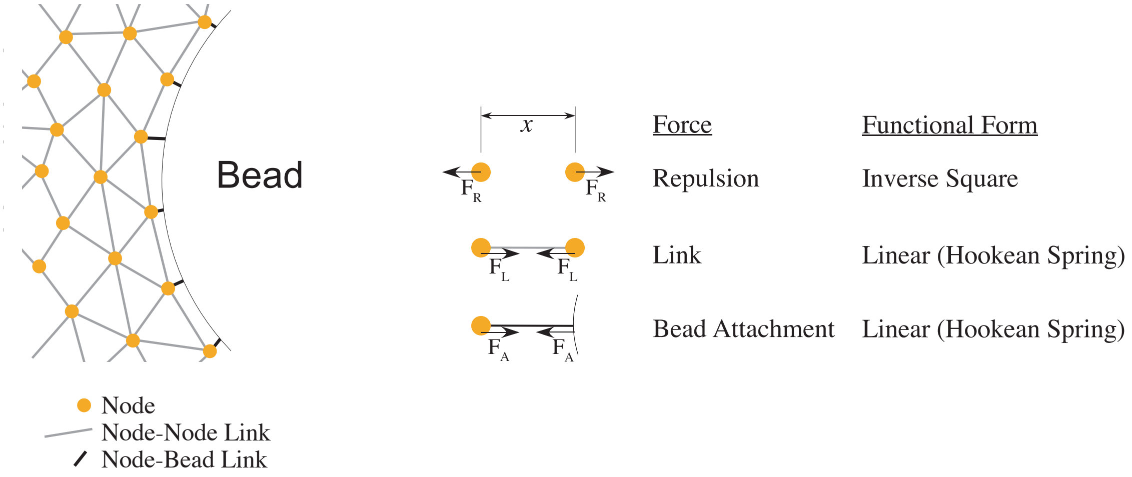

Bead
Node
Node-Node Link
Node-Bead Link
x
Force
Functional Form
F_R
F_R
Repulsion
Inverse Square
F_L
F_L
Link
Linear (Hookean Spring)
F_A
F_A
Bead Attachment
Linear (Hookean Spring)
