# Supplementary figures and images for: In Silico Reconstitution of Actin-Based Symmetry Breaking and Motility
Source: PLoS Biol. 2009 Sep 22;7(9):e1000201. doi: 10.1371/journal.pbio.1000201 (PMC2738636; doi:10.1371/journal.pbio.1000201)

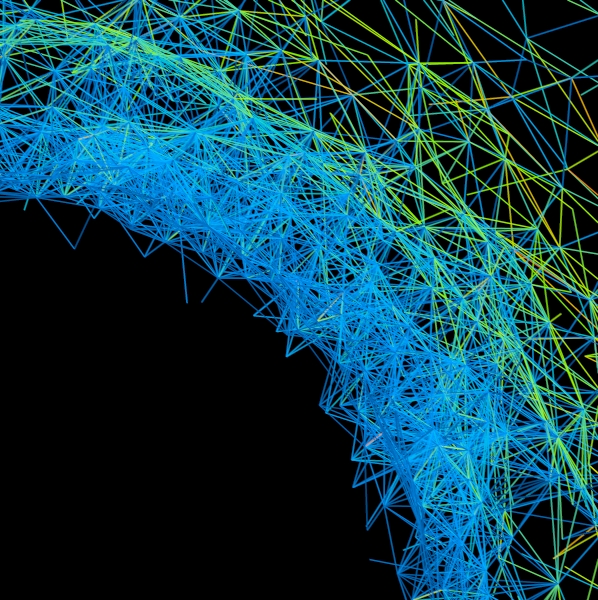

Supplement: Figure S2 — Cross-section of network showing links around bead. The bead would be in the lower left, not plotted so as not to obscure the links. (0.32 MB JPG) [file pbio.1000201.s002.jpg]

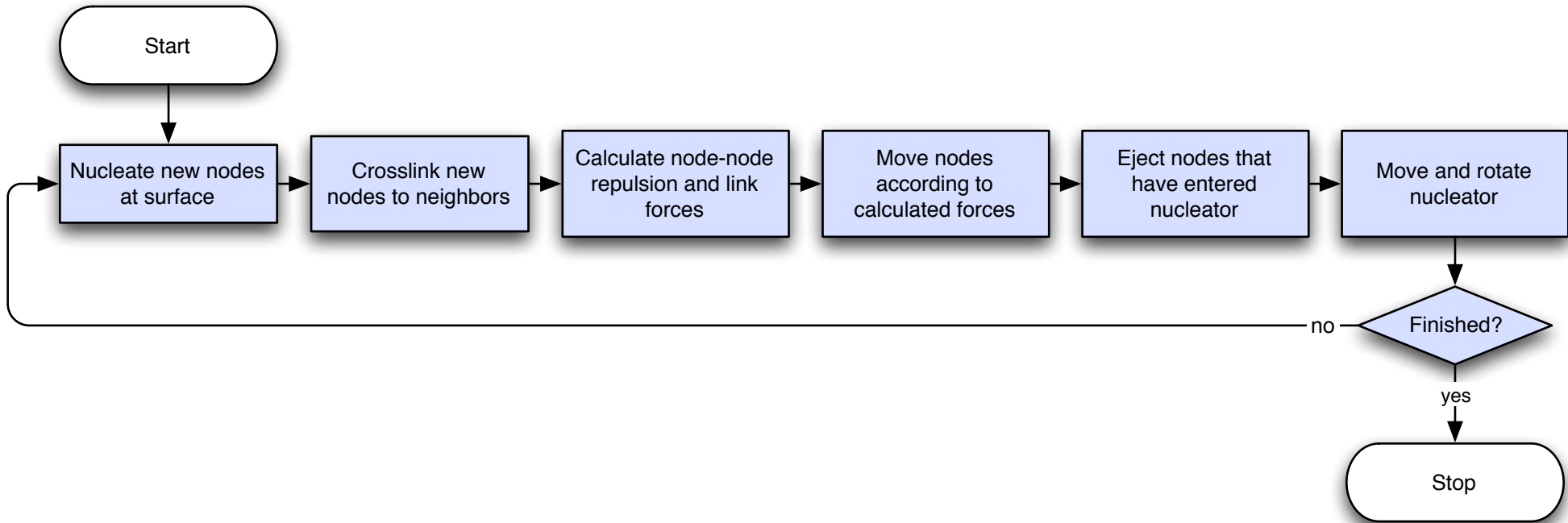

Supplement: Figure S3 — Basic form of the main program loop. (0.03 MB PDF) [file pbio.1000201.s003.pdf]

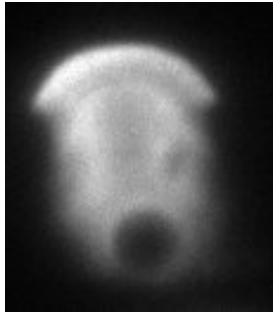

(3D interactive model)

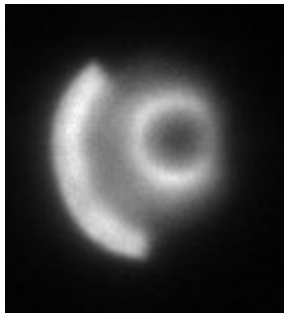

(3D interactive model)

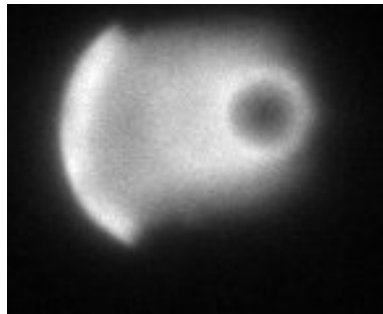

(3D interactive model)

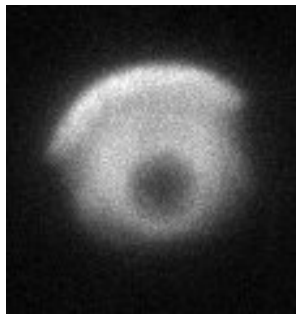

Supplement: Figure S5 — 2-D projections (left) and corresponding interactive 3-D reconstructions (right) of constrained beads (5 µm spacers) showing smooth opening of shell without bilobed structure. Beads are 5-µm diameter. Head space between slide and coverslip is controlled with 5.1-µm diameter glass spacer beads mixed into the reaction. The 2-D projections are the confocal z-stacks summed in the z-direction. The 3-D reconstructions are isosurfaces at low density (transparent) and high density (green), thresholds chosen to best convey the shell morphology. (0.80 MB PDF) [file pbio.1000201.s005.pdf]

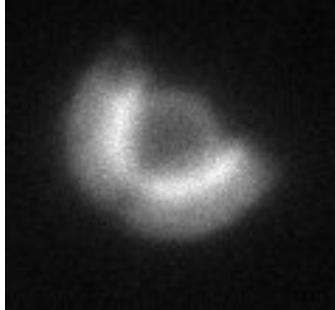

(3D interactive model)

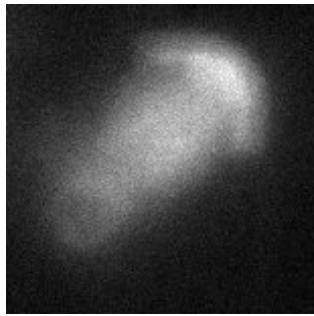

(3D interactive model)

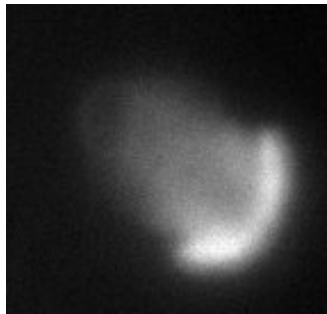

(3D interactive model)

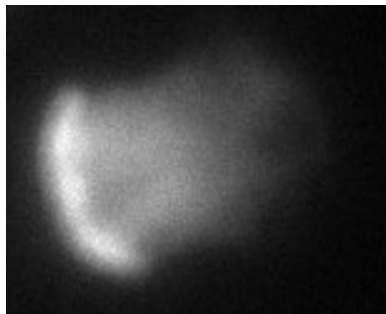

Supplement: Figure S6 — 2-D projections (left) and corresponding interactive 3-D reconstructions (right) of unconstrained beads (15-µm spacers) showing bilobed and trilobed structure. Beads are 5-µm diameter. Head space between slide and coverslip is controlled with 15.5-µm diameter glass spacer beads mixed into the reaction. The 2-D projections are the confocal z-stacks summed in the z-direction. The 3-D reconstructions are isosurfaces at low density (transparent) and high density (green), thresholds chosen to best convey the shell morphology. (0.49 MB PDF) [file pbio.1000201.s006.pdf]

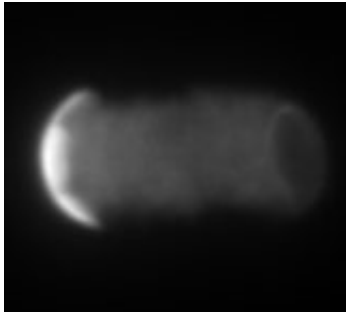

(3D interactive model)

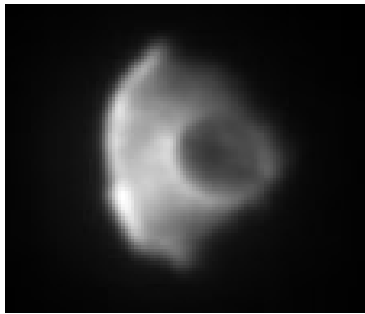

(3D interactive model)

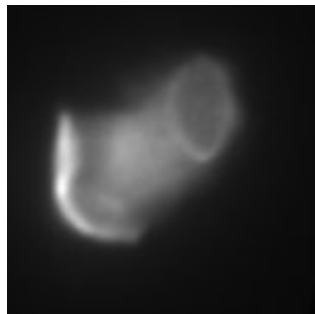

Supplement: Figure S9 — 2-D projections (left) and corresponding interactive 3-D reconstructions (right) of shells and tails from unconstrained ellipsoidal beads showing sideways symmetry breaking and motility. The 2-D projections are the confocal z-stacks summed in the z-direction. The 3-D reconstructions are isosurfaces at low density (transparent) and high density (green), thresholds chosen to best convey the shell morphology and void space to show bead orientation. (0.33 MB PDF) [file pbio.1000201.s009.pdf]

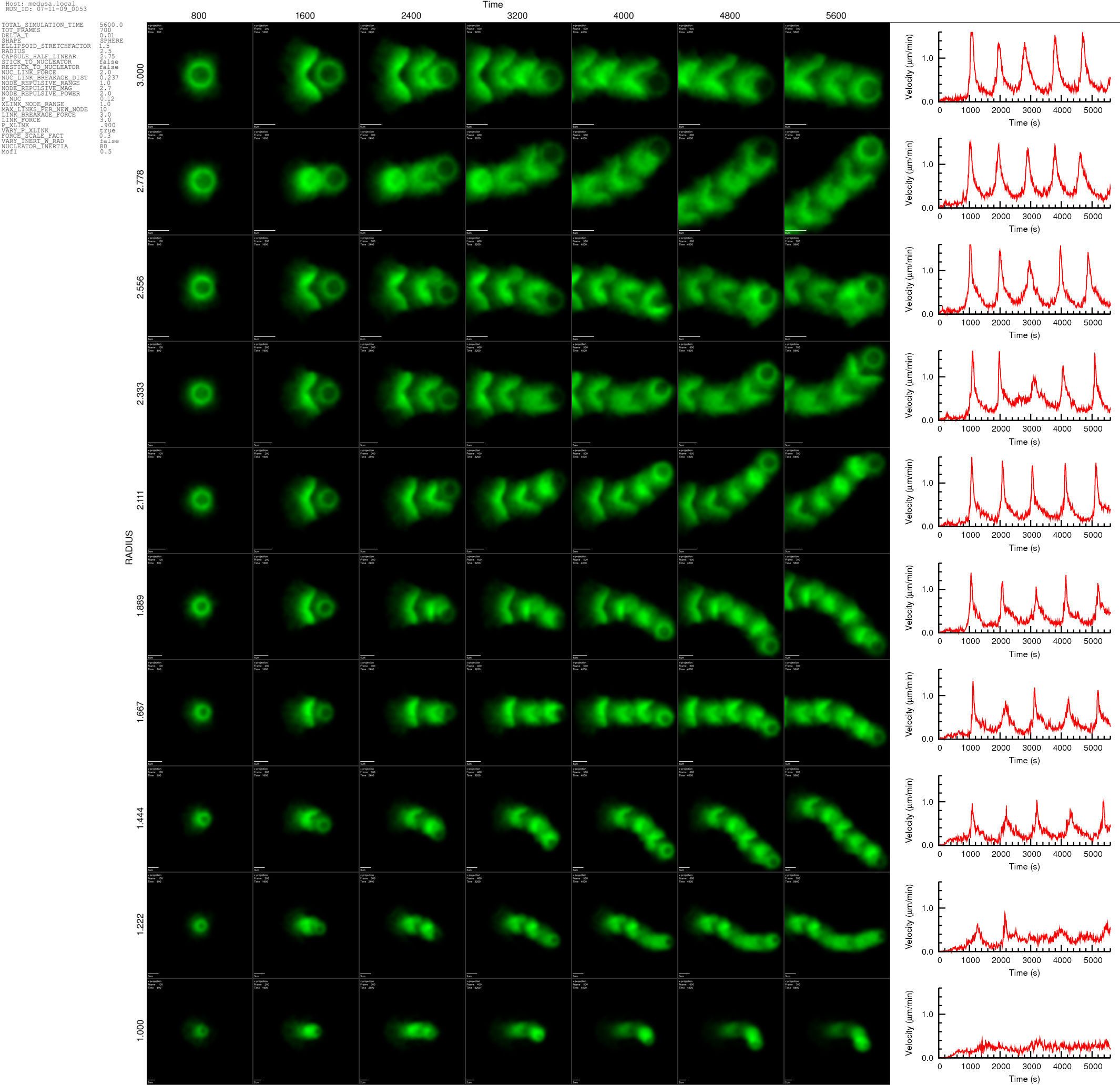

Supplement: Figure S10 — Effect of varying RADIUS. Matrix plot showing 2-D projection of simulation at time points indicated for a range of RADIUS parameter values. Corresponding bead velocity profiles are plotted on the right. The basis parameters are shown in the top left (zoom to view). (0.46 MB JPG) [file pbio.1000201.s010.jpg]

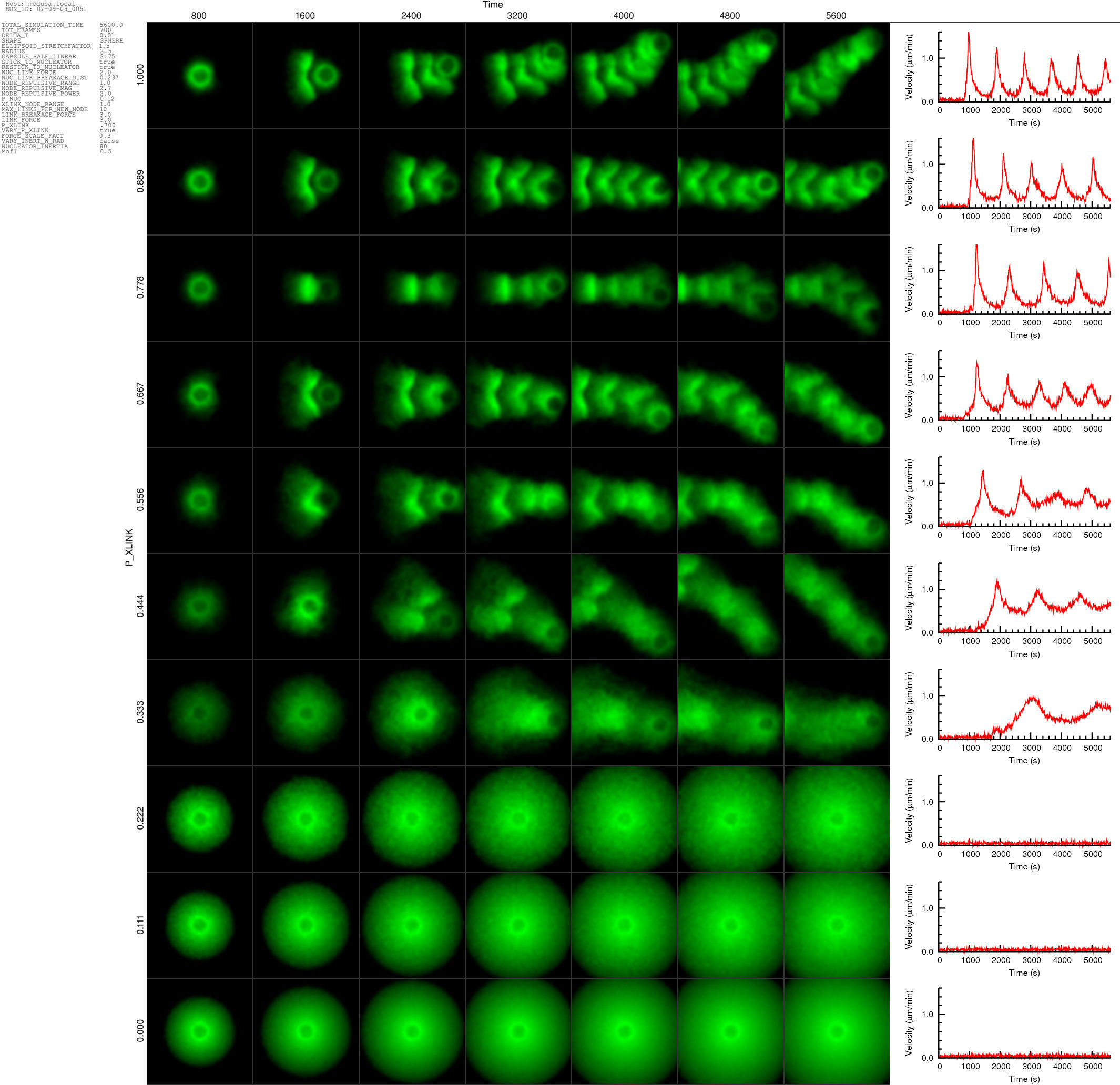

Supplement: Figure S11 — Effect of varying P_XLINK. Matrix plot showing 2-D projection of simulation at time points indicated for a range of P_XLINK parameter values. Corresponding bead velocity profiles are plotted on the right. The basis parameters are shown in the top left (zoom to view). (0.47 MB JPG) [file pbio.1000201.s011.jpg]

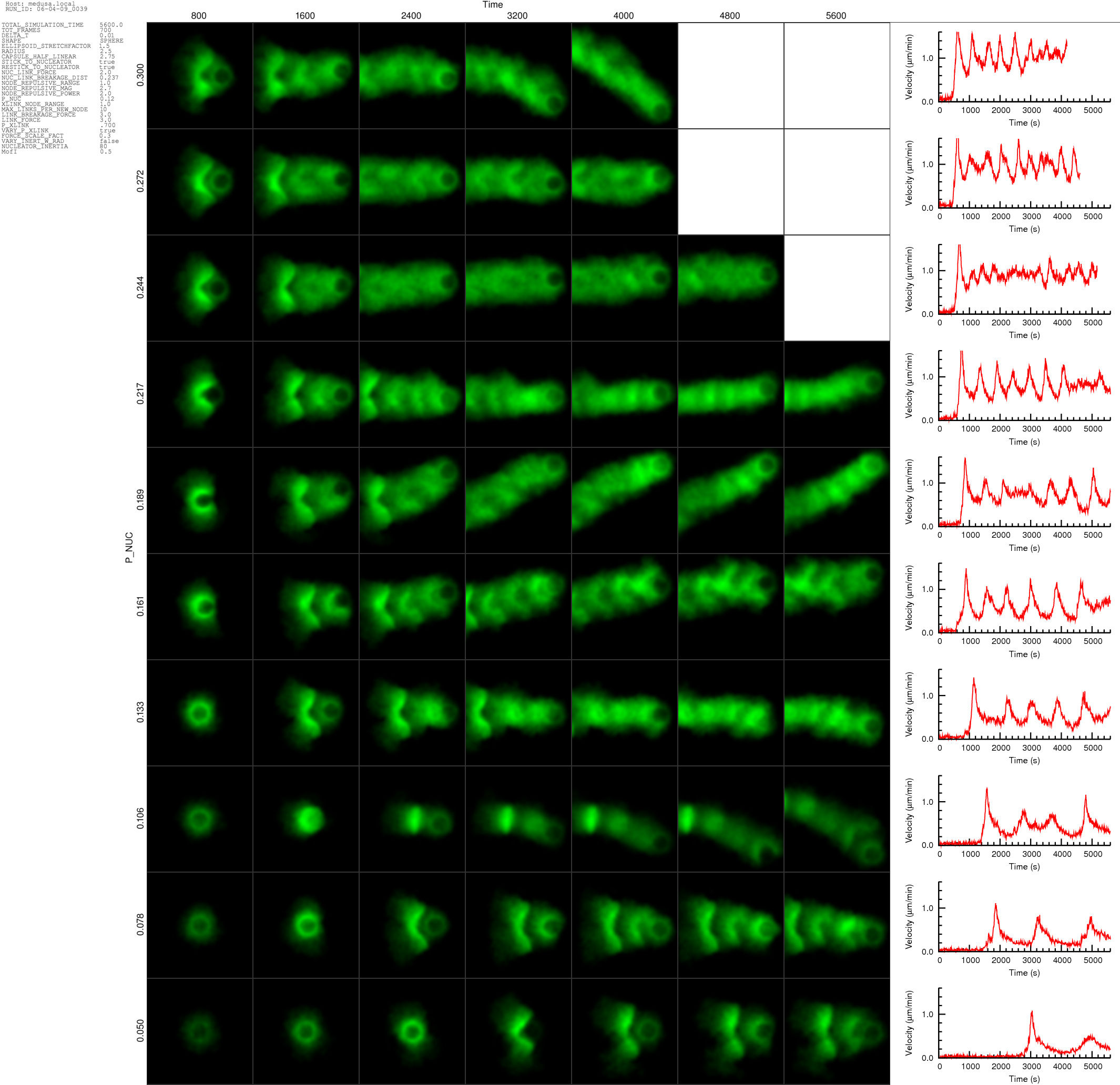

Supplement: Figure S12 — Effect of varying P_NUC. Matrix plot showing 2-D projection of simulation at time points indicated for a range of P_NUC parameter values. Corresponding bead velocity profiles are plotted on the right. The basis parameters are shown in the top left (zoom to view). (0.44 MB JPG) [file pbio.1000201.s012.jpg]

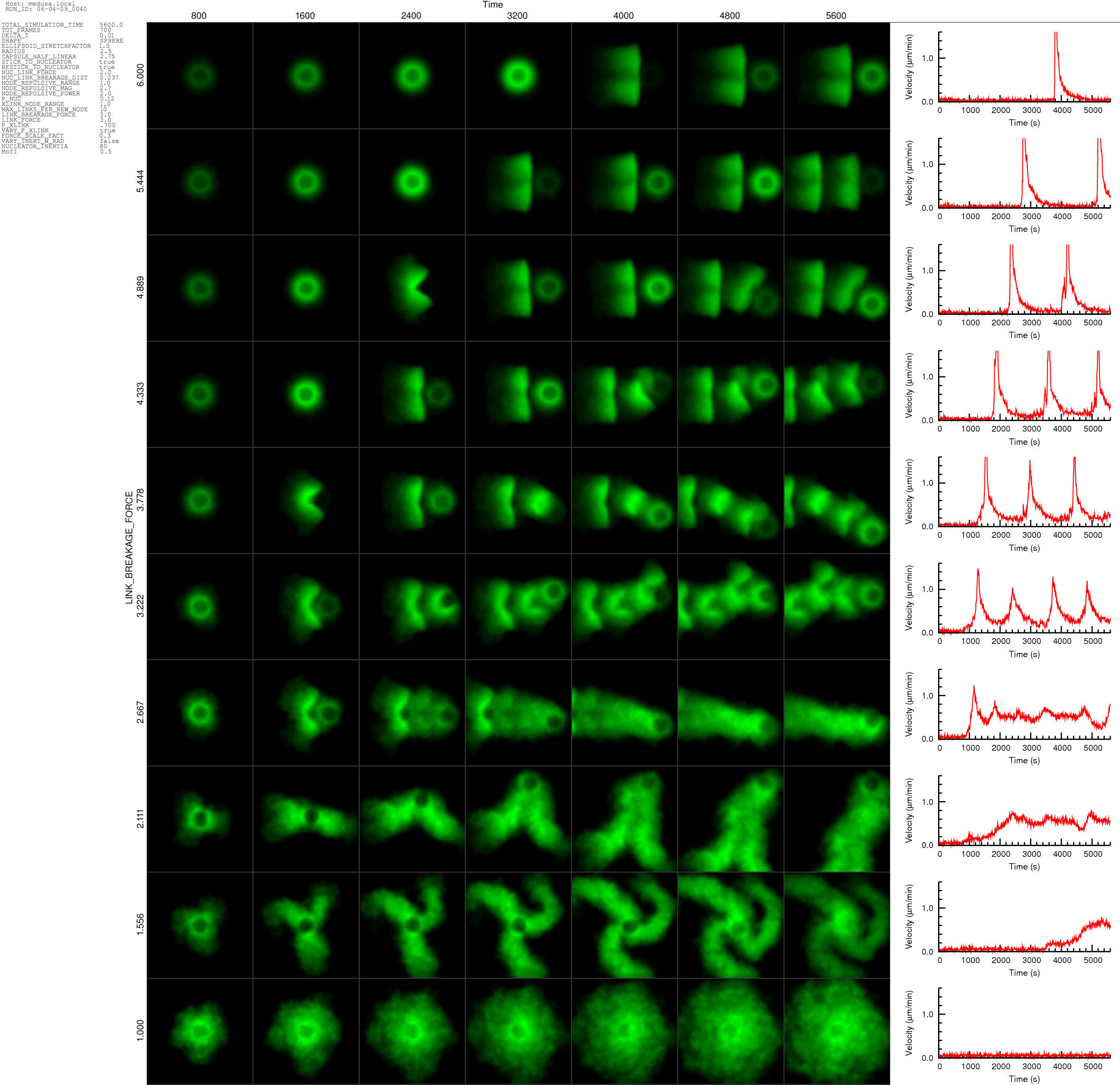

Supplement: Figure S13 — Effect of varying LINK_BREAKAGE_FORCE. Matrix plot showing 2-D projection of simulation at time points indicated for a range of LINK_BREAKAGE_FORCE parameter values. Corresponding bead velocity profiles are plotted on the right. The basis parameters are shown in the top left (zoom to view). (0.44 MB JPG) [file pbio.1000201.s013.jpg]

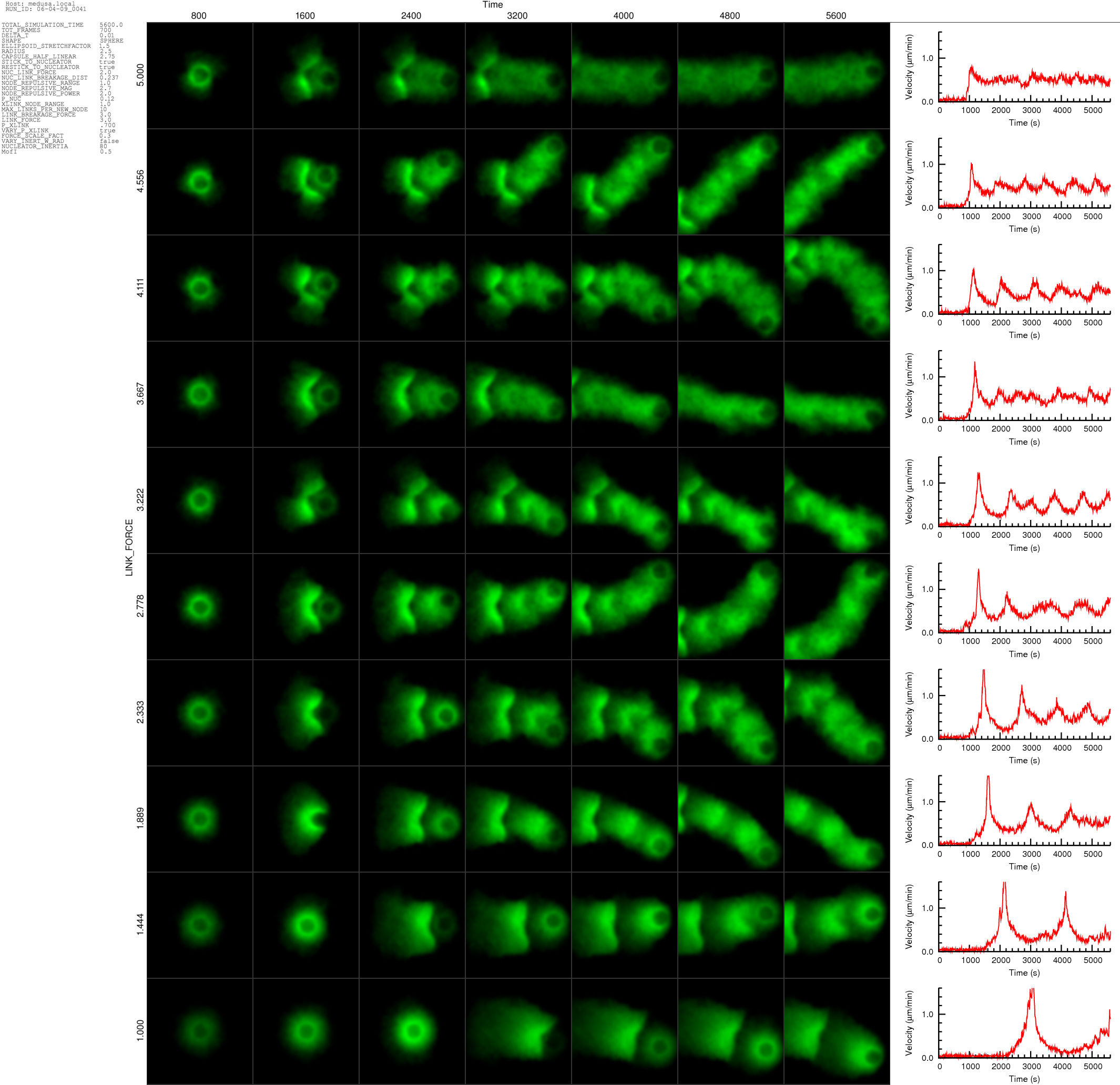

Supplement: Figure S14 — Effect of varying LINK_FORCE. Matrix plot showing 2-D projection of simulation at time points indicated for a range of LINK_FORCE parameter values. Corresponding bead velocity profiles are plotted on the right. The basis parameters are shown in the top left (zoom to view). (0.44 MB JPG) [file pbio.1000201.s014.jpg]

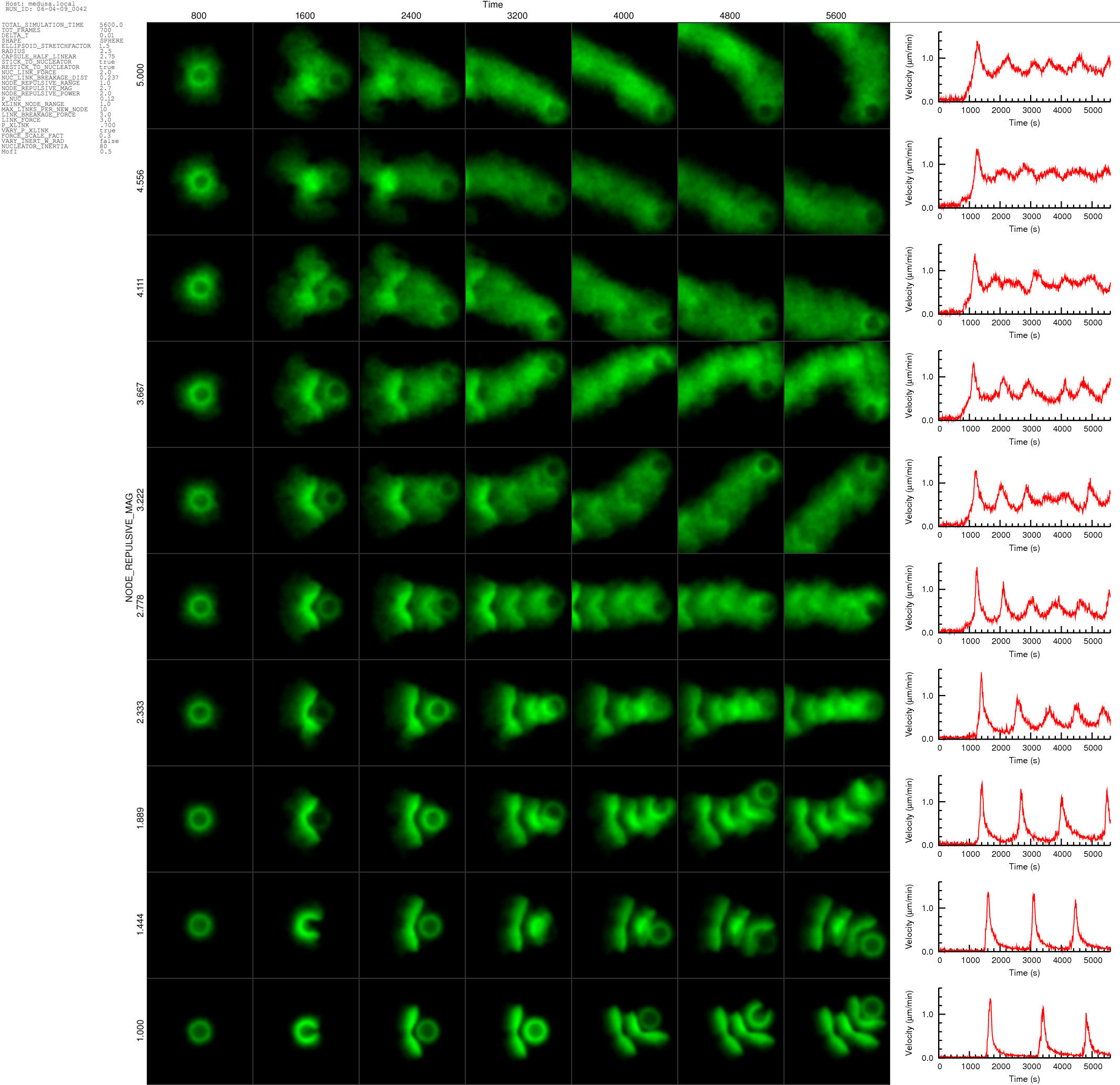

Supplement: Figure S15 — Effect of varying NODE_REPULSIVE_MAG. Matrix plot showing 2-D projection of simulation at time points indicated for a range of NODE_REPULSIVE_MAG parameter values. Corresponding bead velocity profiles are plotted on the right. The basis parameters are shown in the top left (zoom to view). (0.45 MB JPG) [file pbio.1000201.s015.jpg]

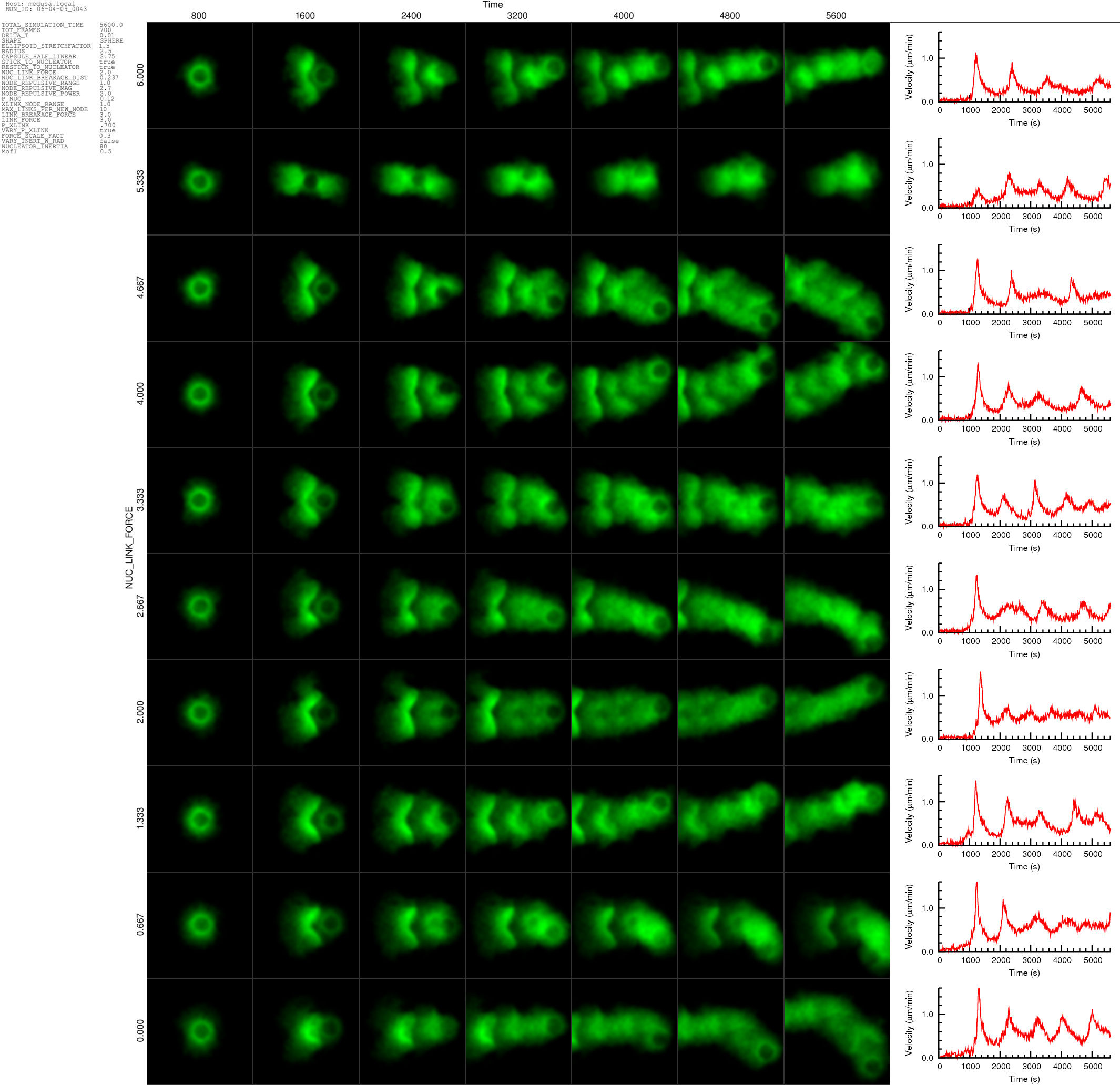

Supplement: Figure S16 — Effect of varying NUC_LINK_FORCE. Matrix plot showing 2-D projection of simulation at time points indicated for a range of NUC_LINK_FORCE parameter values. Corresponding bead velocity profiles are plotted on the right. The basis parameters are shown in the top left (zoom to view). (0.44 MB JPG) [file pbio.1000201.s016.jpg]

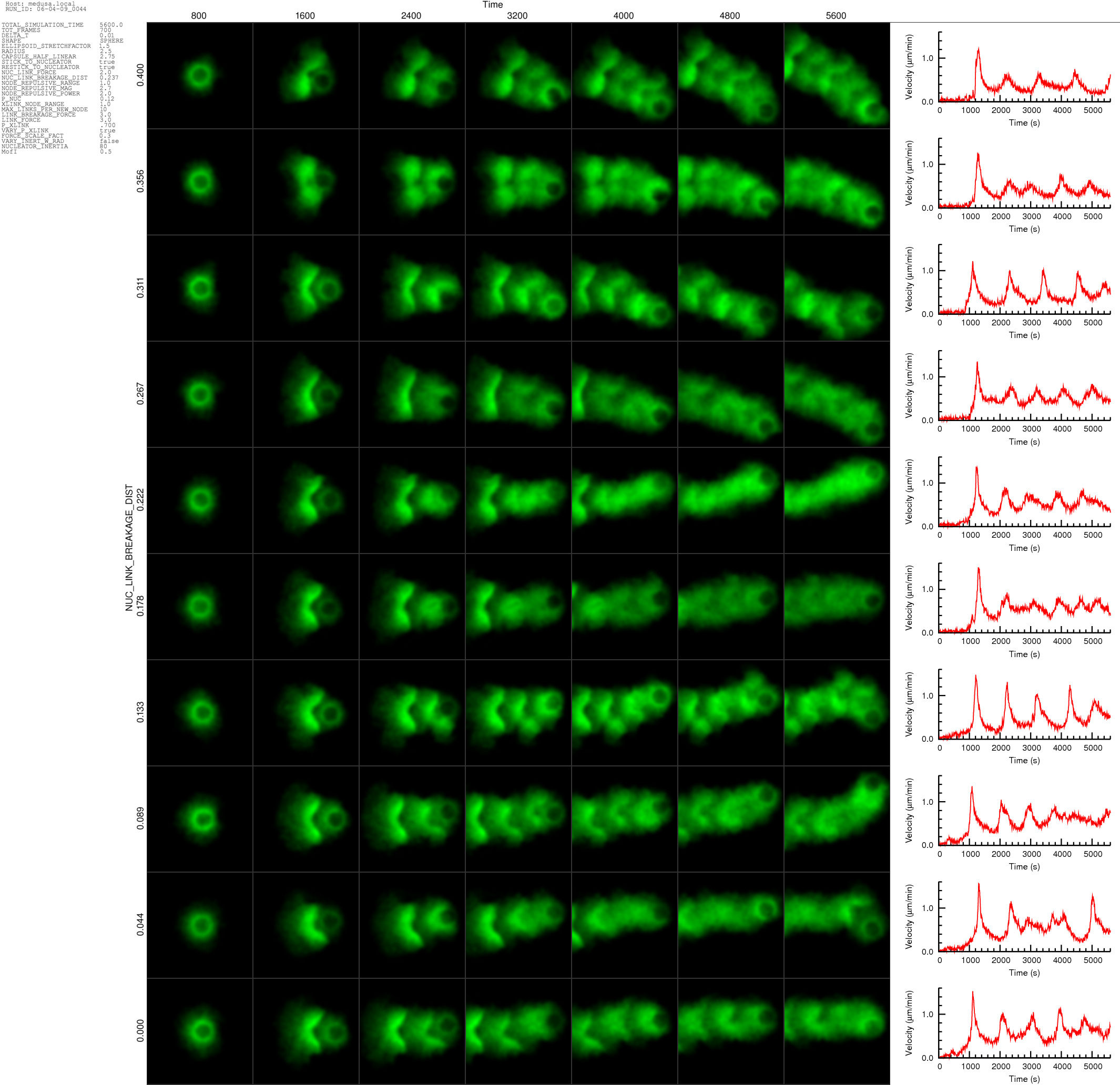

Supplement: Figure S17 — Effect of varying NUC_LINK_BREAKAGE_DIST. Matrix plot showing 2-D projection of simulation at time points indicated for a range of NUC_LINK_BREAKAGE_DIST parameter values. Corresponding bead velocity profiles are plotted on the right. The basis parameters are shown in the top left (zoom to view). (0.46 MB JPG) [file pbio.1000201.s017.jpg]

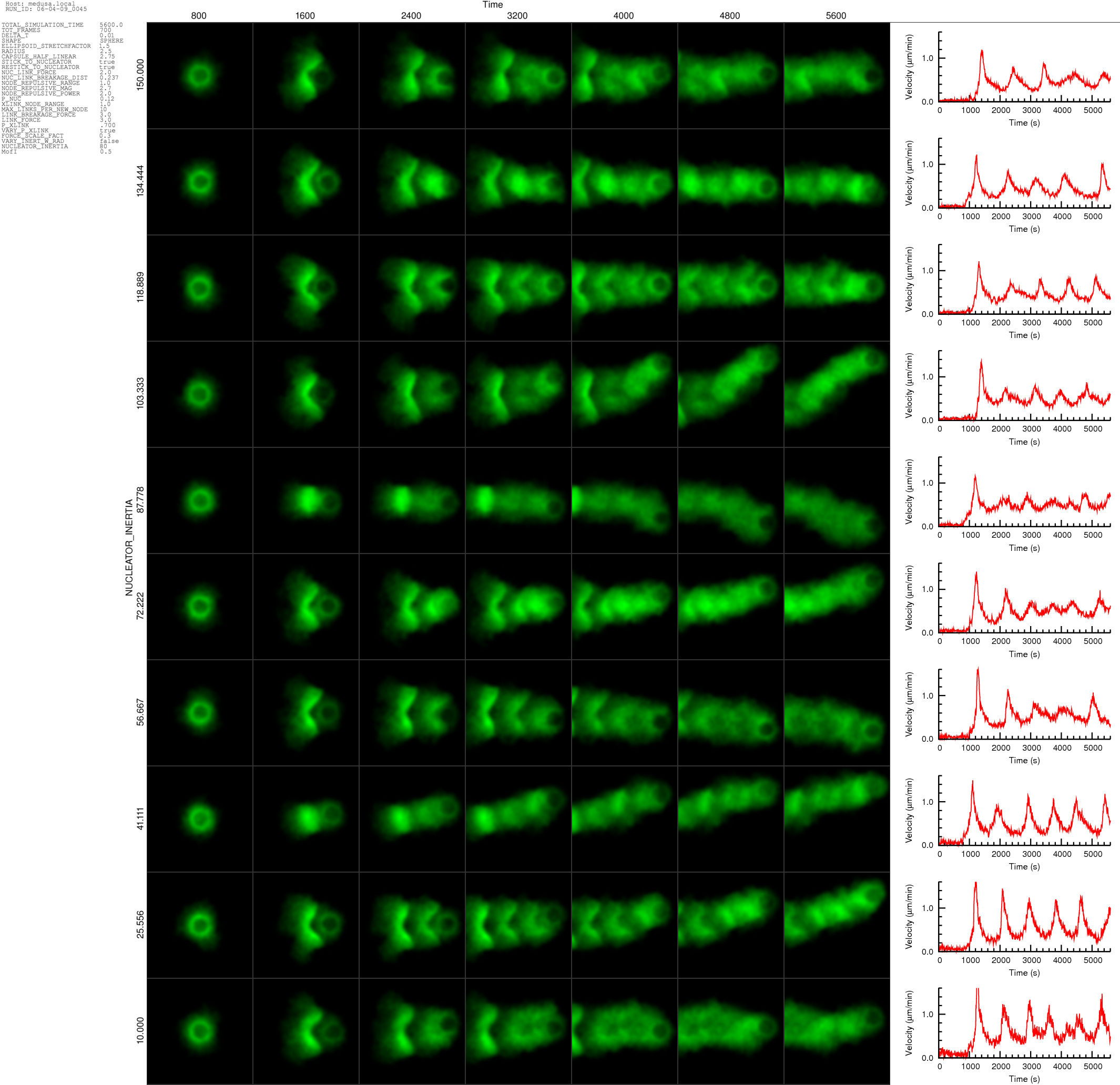

Supplement: Figure S18 — Effect of varying NUCLEATOR_INERTIA. Matrix plot showing 2-D projection of simulation at time points indicated for a range of NUCLEATOR_INERTIA parameter values. Corresponding bead velocity profiles are plotted on the right. The basis parameters are shown in the top left (zoom to view). (0.45 MB JPG) [file pbio.1000201.s018.jpg]

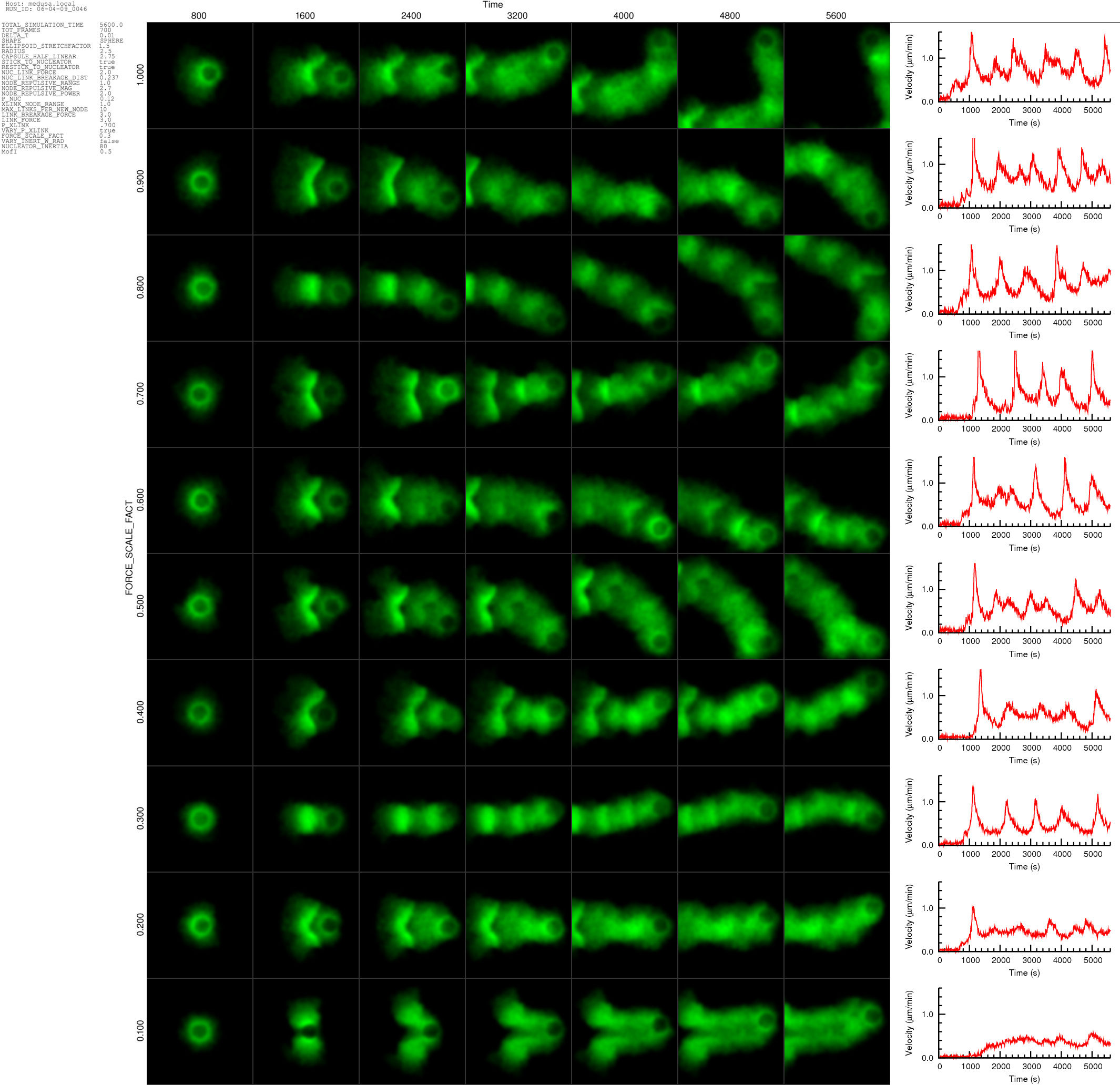

Supplement: Figure S19 — Effect of varying FORCE_SCALE_FACT. Matrix plot showing 2-D projection of simulation at time points indicated for a range of FORCE_SCALE_FACT parameter values. Corresponding bead velocity profiles are plotted on the right. The basis parameters are shown in the top left (zoom to view). (0.46 MB JPG) [file pbio.1000201.s019.jpg]

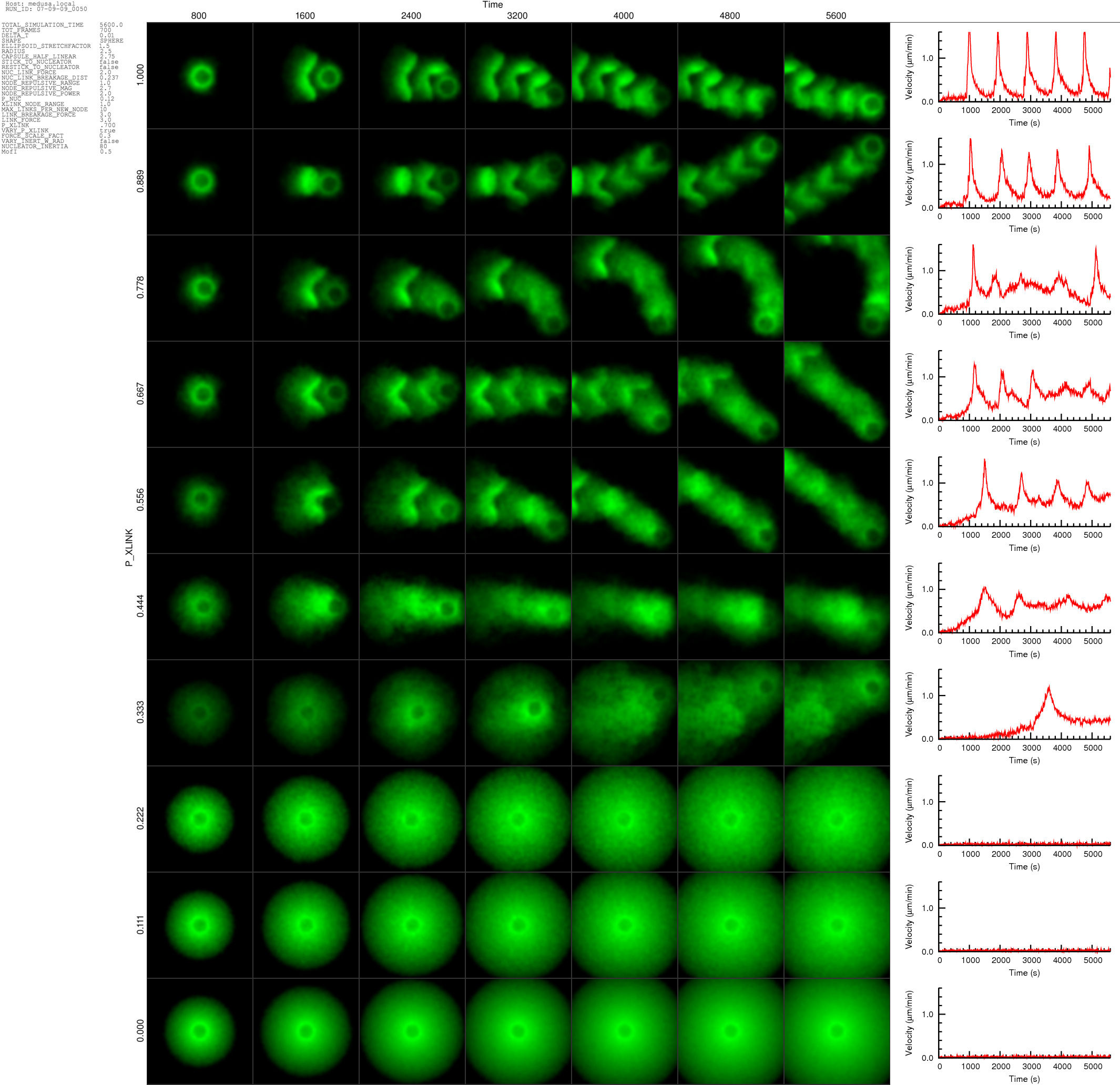

Supplement: Figure S20 — Effect of varying P_XLINK with no bead-network friction. Matrix plot showing 2-D projection of simulation at time points indicated for a range of P_XLINK parameter values. Corresponding bead velocity profiles are plotted on the right. The basis parameters are shown in the top left (zoom to view). (0.47 MB JPG) [file pbio.1000201.s020.jpg]

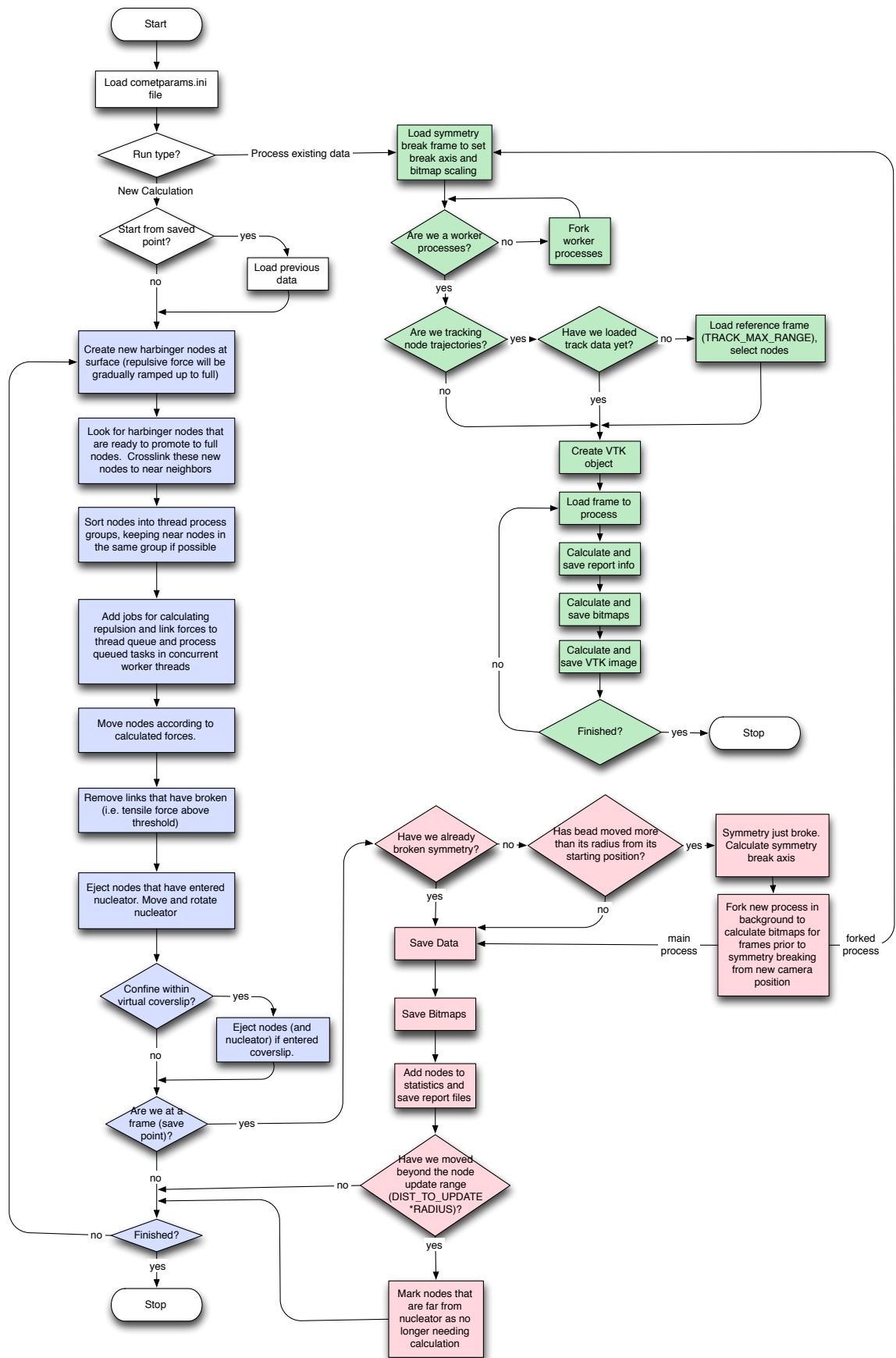

Supplement: Figure S21 — Detailed program flow. (0.07 MB PDF) [file pbio.1000201.s021.pdf]

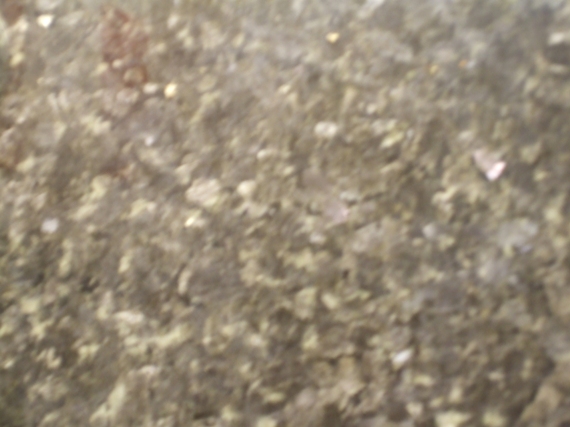

Supplement: Protocol S2 — Source code and parameter control file (under GPL open source license). (comet_src_v0.2.zip) (0.34 MB ZIP) [file pbio.1000201.s023.zip › comet/nuctex.jpg]
