# Supplementary material for: In Silico Reconstitution of Actin-Based Symmetry Breaking and Motility
Source: PLoS Biol. 2009 Sep 22;7(9):e1000201. doi: 10.1371/journal.pbio.1000201 (PMC2738636; doi:10.1371/journal.pbio.1000201)
Supplement: Protocol S1 — Supporting text. (SupportingText.pdf) (7.62 MB PDF) [file pbio.1000201.s022.pdf]

Protocol 1:

Supporting Text for *In Silico* Reconstitution of Actin-Based Symmetry Breaking and Motility

Mark J Dayel[✉,†], Orkun Akin[§], Mark Landeryou[||],
Viviana I Risca[¶], Alex Mogilner[‡], R. Dyche Mullins[§]

✉Correspondence: markdayel@gmail.com

†Miller Institute for Basic Research in Science, University of California Berkeley
§Department of Cellular and Molecular Pharmacology, University of California San Francisco
||Department of Mechanical Engineering, University College London
¶Biophysics Graduate Group, University of California, Berkeley
‡Departments of NPB and Mathematics, University of California Davis

- Figures in this PDF contain 3D models that require Adobe Acrobat to view (click on the interactive figures to rotate, zoom etc.).
- See http://www.dayel.com/comet for more information about the model and to download the current version of the code.

Contents

S1 Outline of the Model **4**
S1.1 Overview . . . 4
S1.2 Model Assumptions . . . 5
S1.3 Relation of the model assumptions to theories of and data on actin dynamics . . . 5
S1.4 Overview of Model implementation . . . 6
S1.5 Model parameter names . . . 7

S2 3D figures **8**

S3 Model Robustness **13**
S3.1 Increasing Radius produces pulsatile motion . . . 13
S3.2 Shell thickness . . . 13
S3.3 Shell Flatness . . . 13
S3.4 Multiple tails . . . 13
S3.5 Pulsatile motion with no bead-network friction . . . 14

S4 Simulation Settings: Model Parameters **26**
S4.1 Run Time . . . 26
S4.2 Nucleator Geometry . . . 26
S4.3 Nucleator Attachments . . . 26
S4.4 Node-node repulsion . . . 26
S4.5 Node links . . . 27
S4.6 Drag . . . 27

S5 Simulation Settings: Display **28**
S5.1 Producing Bitmaps . . . 28
S5.2 Producing VTK output . . . 28
S5.3 Bitmap display settings . . . 28
S5.4 VTK settings (3D) . . . 29
S5.5 Misc . . . 30
S5.6 Settings that apply to both bitmaps and VTK . . . 30

S6 Installing the program **31**
S6.1 Precomiled binary for OS X . . . 31
S6.2 Compiling from source . . . 31
S6.3 Dependencies . . . 31
S6.4 Platform Specific Information . . . 31

S7 Running the program **32**
S7.1 Runtime prerequisites . . . 32
S7.2 Command line syntax . . . 32
S7.3 Running the program on a cluster . . . 32

S8 Model Implementation **33**
S8.1 Program Flow . . . 33
S8.2 Implementation in C++ . . . 33

References **36**

List of Tables

S1 Model assumptions based directly on experimental data 5
S2 Model assumptions inferred from experimental data or physical assumptions 5
S3 Corresponding simulation parameter names in the main text and in the code 7

List of Figures

S1 Diagram of network and forces acting on nodes 4
S2 Cross-section of network showing links around bead 4
S3 Basic form of the main program loop 5
S4 Interactive 3D reconstructions of *in silico* shells from unconstrained and constrained beads 8
S5 2D projections and corresponding interactive 3D reconstructions of constrained beads 9
S6 2D projections and corresponding interactive 3D reconstructions of unconstrained beads 10
S7 Interactive 3D view of *in silico* network trajectory relative to bead during smooth motion. 11
S8 Interactive 3D view of *in silico* network trajectory relative to half-coated capsule during smooth motion 11
S9 2D projections and corresponding interactive 3D reconstructions of shells and tails from unconstrained ellipsoidal beads 12
S10 Effect of varying `RADIUS` 15
S11 Effect of varying `P_XLINK` 16
S12 Effect of varying `P_NUC` 17
S13 Effect of varying `LINK_BREAKAGE_FORCE` 18
S14 Effect of varying `LINK_FORCE` 19
S15 Effect of varying `NODE_REPULSIVE_MAG` 20
S16 Effect of varying `NUC_LINK_FORCE` 21
S17 Effect of varying `NUC_LINK_BREAKAGE_DIST` 22
S18 Effect of varying `NUCLEATOR_INERTIA` 23
S19 Effect of varying `FORCE_SCALE_FACT` 24
S20 Effect of varying `P_XLINK` with no bead-network friction. 25
S21 Detailed program flow 35

(Supplemental Videos S1–S22 are available as separate files)

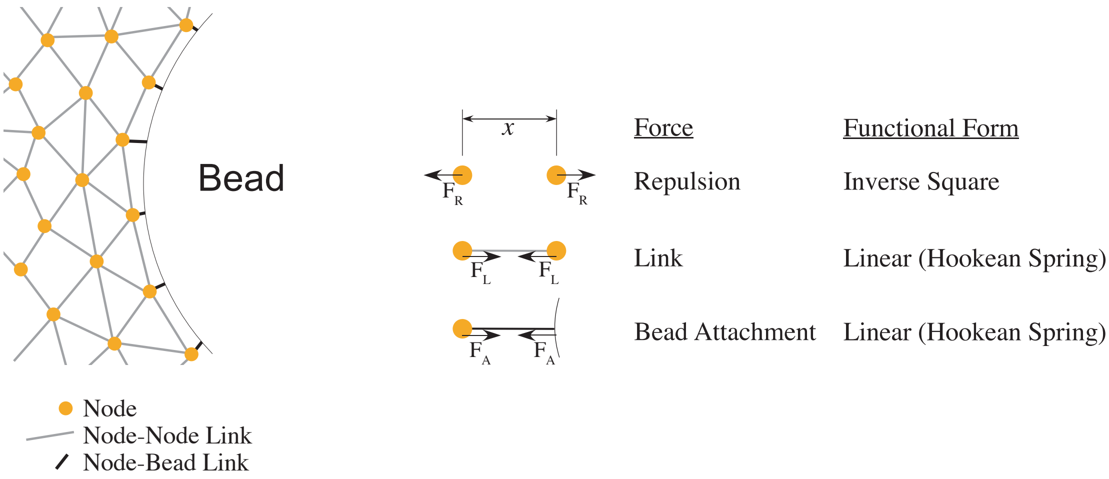

Figure S1: Diagram of network and forces acting on nodes

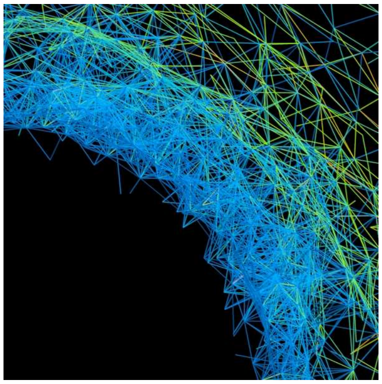

Figure S2: Cross-section of network showing links around bead (the bead would be in the lower left—not plotted so as not to obscure the links)

S1 Outline of the Model

S1.1 Overview

The `comet` program is a Monte-Carlo/Lagrangian model that calculates the 3 dimensional positions of a large number of 'nodes' representing material in an actin network (diagrammed in figure S1 and an example shown in figure S2). For each timestep `DELTA_T`, nodes move a displacement proportional to the force acting upon them. There is no inertia, since this is a low Reynolds number regime. The forces acting on each node are as follows:

- Repulsive forces between nodes
- Link forces between nodes
- Link forces between node and nucleator

The core of the program is essentially the iteration loop shown in figure S3. Nodes are added, forces calculated, and node positions updated as shown. The model parameters are described in more detail in section S4.

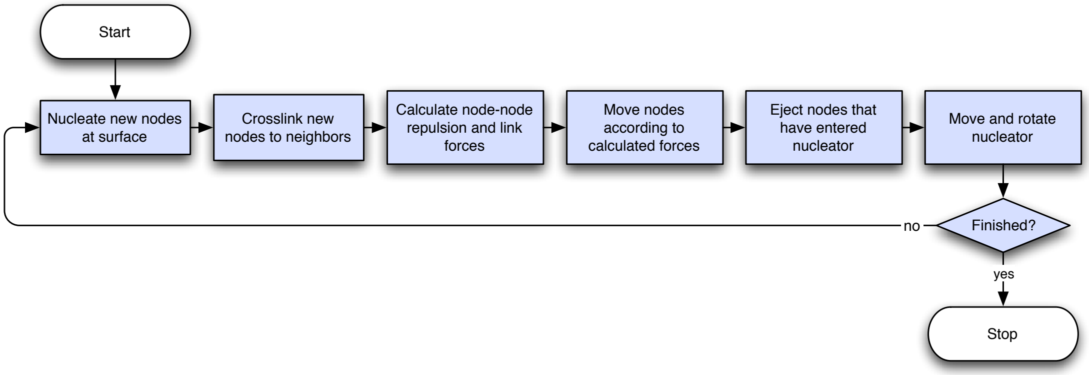

Figure S3: Basic form of the main program loop

S1.2 Model Assumptions

Our objective has been to model a viscoelastic actin network as simply as possible. Where possible, we have based the model assumptions on experimental data (Table S1); where no data is available, we have chosen simple assumptions about the behavior (Table S2). The basis for some of these assumptions is discussed more fully in Section S1.3.

Model Behavior	Experimental Basis
Nodes deposited only at bead surface	Polymerization localized to bead surface *in vitro*(Paluch et al., 2006; Akin and Mullins, 2008; Delatour et al., 2008)
Constant rate of node deposition	Constant rate of actin deposition *in vitro*(Akin and Mullins, 2008)
Node-bead attachments	Bead is attached to tail, likely via Arp2/3 ActA attachments (Marcy et al., 2004)

Table S1: Model assumptions based directly on experimental data

Model Behavior	Basis
Nodes crosslinked only at bead surface	Polymerization localized to bead surface *in vitro* therefore entanglement can only occur at surface
Uniform nucleation across surface	Uniform ActA coating on bead
Links as Hookean springs	Simplest form; Actin as entropic spring
Links break above certain tension	Simplest form; network must have limited strength, yielding by disentanglement, Arp2/3-filament release or filament breakage
Inverse square repulsion force	Simplest form
Velocity proportional to force	Low Reynold's number regime
Limit on Links per Node	Physical limit on how entangled network can be

Table S2: Model assumptions inferred from experimental data or physical assumptions

S1.3 Relation of the model assumptions to theories of and data on actin dynamics

Our model is mesoscopic and does not consider the detailed microscopic mechanisms of force generation by actin filaments growing against a curved surface. We simply use the theories (reviewed in (Mogilner, 2006) supported by

the data (Kovar and Pollard, 2004; Footer et al., 2007) suggesting that individual filaments can grow against pN-range forces. Despite the fact that we do not consider respective pushing forces at the surface explicitly, their existence is crucial, because they maintain the active outward pushing stress at the inner boundary of the shell generating the passive viscoelastic radial and transverse stresses within the shell. The justification for not considering the pushing forces explicitly is as follows.

Three regimes of actin filament growth at the bead or Listeria surface are possible: diffusion limited (Plastino et al., 2004), stress limited (van der Gucht et al., 2005), and polymerization limited. In the first case, the dense actin gel hinders diffusion of the G-actin to the surface where the polymerization takes place, and the filament growth slows down. In the second case, the radial compression of the expanding actin shell stalls the filament growth. The diffusion-limited regime, however, is only the case when the mesh size of the actin network is small enough (of the order of 30 nm or less (Mogilner and Edelstein-Keshet, 2002). In our case, estimates of the data (Akin and Mullins, 2008) suggest that the actin gel mesh size is greater, $\zeta \sim 0.1$ µm. In this case, and when the radius of the actin shell is of the order of the bead's radius, the radial stress at the beads surface $\sigma \sim Y$, where Y is the Young's modulus of the actin gel (Sekimoto et al., 2004). The Young's modulus can be estimated roughly as $Y = \frac{k_B T l_p}{\zeta^4}$ (MacKintosh et al., 1995), where $K_B T \sim 0.004 pN \times$µm is the thermal energy, and $l_p \sim 10$µm is the actin filament's persistence length. For $\zeta \sim 0.1$ µm, $\sigma \sim Y \sim 400 pN/\mu m^2$, and the force per filament is of the order of $\sigma \times \zeta^2 \sim 4 pN$, well below the estimated stall force (reviewed in (Mogilner, 2006)).

These estimates suggest that we can assume simply that the actin growth at the surface is equal to a constant polymerization rate. The growing filaments, of course, also produce force, which is not constant: this force balances the growing radial shell compression, but it does not slow down the growth significantly. Mathematically, this assumption translates into the constant rate with which the nascent network nodes are deposited at the random locations at the surface. Following the observations, we assume that the polymerization takes place only at the surface, and that there is no appreciable depolymerization of actin.

The assumption that the nascent nodes are attached to the surface by elastic springs and that these springs break at characteristic yield strain is equivalent, when averaged, to an effective viscous drag (Tawada and Sekimoto, 1991). The fact that the transient attachments of the actin filaments do produce such resistance to propulsion is established (Bernheim-Groswasser et al., 2002; Trichet et al., 2007).

Modeling of the actin gels with nodes connected by elastic springs is well established (Bottino and Fauci, 1998; Shafrir and Forgacs, 2002). In our model, many elastic links between the neighboring nodes oriented in random directions correspond to the isotropic elasticity of the actin gel. This is the simplest case; there are no indications of mechanical anisotropy of the Arp2/3-mediated actin gels. At small deformations, the *in silico* gel exhibits linear elasticity; existing estimates (Boal, 2001) demonstrate that the mechanical properties of such gel are robust with respect to the exact orientation, number and lengths of the spring-like connections between the nodes. The dimensional magnitude of the Young modulus of our *in silico* gel, which in principle is the parameter sensitive to the springs' lengths, is not important for the model behavior, because we assume that the filaments' growth is force-independent, and that the gel breaking is strain-limited, rather than stress-limited.

In our model, the elastic behavior arises from small deformations of the elastic springs, while the viscous behavior ensues when a characteristic yield strain is exceeded, the springs snap, and the respective nodes start flowing relative to each other. This behavior corresponds indirectly to Kelvin model of viscoelastic materials (Bird et al., 1977). The yield-strain-limiting behavior of the actin gel was detected many times, recently in (Gardel et al., 2006). It corresponds most likely not to breaking of individual filaments (Tsuda et al., 1996) or proteins connecting the filaments (Fujiwara et al., 2002), which would be stress-limiting and occur at greater forces, but to disentanglement of stretching filament arrays, which is a geometric phenomenon and therefore is strain-limiting.

S1.4 Overview of Model implementation

The nucleator object is treated as incompressible i.e. if during an iteration a node enters the nucleator, then in the next iteration it is simply moved out of the nucleator along a normal to the nucleator surface.

Nodes are nucleated at a constant rate, proportional to `P_NUC`, at the nucleator surface. To allow it to find an equilibrium position before being crosslinked into the network, a new node has its `harbinger` flag set when created, it experiences only repulsive forces for `CROSSLINKDELAY` iterations. Crosslinks are then formed as follows: All nodes with within `XLINK_NODE_RANGE` are counted, and links are either formed in random order until the number of crosslinks reaches `MAX_LINKS_PER_NODE`. Once a link is formed, its original distance is stored and used to calculate link forces. If the link is stretched or compressed away from its original length it behaves as a Hooke's

Law spring and exerts a force proportional to, and opposing, the displacement. The scale factor for this force is `LINK_FORCE`. This is to simulate an actin filament acting as an entropic spring by flexing motions. If the link force exceeds `LINK_BREAKAGE_FORCE` then the link breaks.

The nucleator is allowed to move and rotate, subject to displacement and torque vectors from the summed node repulsion from the nucleator, and the nucleator-node link forces. A full treatment of nucleator inertia is beyond the scope of the current model, and drag is simply scaled by a supplied parameter `NUCLEATOR_INERTIA` multiplied by the node inertia, and similarly the nucleator moment of inertia is scaled by the supplied parameter `MofI`. As a first approximation of how this should change with nucleator size, we scale the inertia and moment of inertia by the radius (or radius and length for long axes of the ellipsoids and capsules) if the `VARY_INERT_W_RAD` parameter is set. Given that this is not drag through a Newtonian fluid, but largely a product of complex fluid and network drag forces, this may not be very accurate. On the other hand Figure S18 shows that the behavior is not very sensitive to the `NUCLEATOR_INERTIA` parameter anyway.

Output files are saved as jpgs for the x,y and z projections (convolved with a Gaussian to make it look like a microscope image). Post processing routines can produce 3D rendering jpgs, or interactive 3D renderings on-screen. Also, post-processing 3D rendering of a single image will trigger the program to also write a vrml file to allow the 3D view to be imported into other software (e.g. Acrobat 3D etc.). Note: the program calls the Imagemagick `convert` program to add text to the images and save as jpgs and calls `bzip2` to compress the data files.

S1.5 Model parameter names

For aesthetic reasons, we refer to several model parameters in the text with subscripted letters. These correspond the simulation model parameters listed in Table S3. See Section S4 for more information.

Parameter in text	Parameter in Model	Description
P_{XL}	`P_XLINK`	Probability of forming crosslink
F_L	`LINK_FORCE`	Spring constant for node-node links
F_{BL}	`LINK_BREAKAGE_FORCE`	Force threshold above which node-node links break
M_R	`NODE_REPULSIVE_MAG`	Magnitiude of node repulsive force

Table S3: Corresponding simulation parameter names in the main text and in the code

S2 3D figures

(Acrobat required for 3D)

(Acrobat required for 3D)

Figure S4: Interactive 3D reconstructions of *in silico* shells from unconstrained (top) and constrained (bottom) beads showing linear crack or bi-lobed structure. Beads are 5-μm diameter. For the constrained condition head-space between slide and coverslip is controlled with 5.1-μm diameter glass spacer beads mixed into the reaction. For the unconstrained, 15.5-μm diameter glass spacer beads were used.

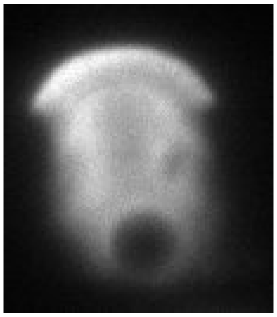

(Acrobat required for 3D)

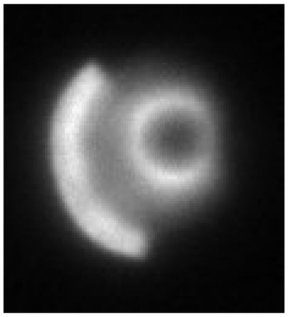

(Acrobat required for 3D)

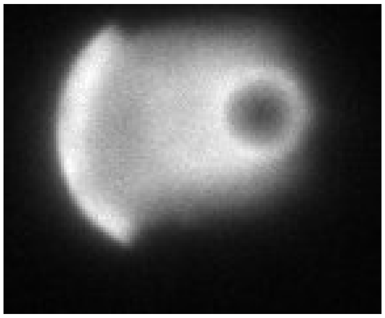

(Acrobat required for 3D)

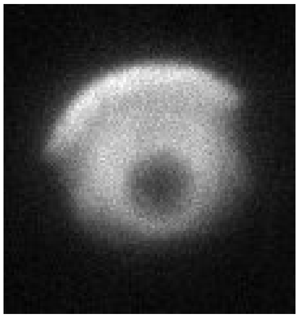

(Acrobat required for 3D)

Figure S5: 2D projections (left) and corresponding interactive 3D reconstructions (right) of constrained beads (5-µm spacers) showing smooth opening of shell without bi-lobed structure. Beads are 5-µm diameter. Head-space between slide and coverslip is controlled with 5.1-µm diameter glass spacer beads mixed into the reaction. 2D projections are the confocal z-stacks summed in the z-direction. 3D reconstructions are isosurfaces at low (transparent) and high (green) density, thresholds chosen to best convey the shell morphology.

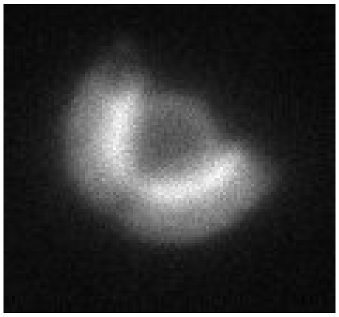

(Acrobat required for 3D)

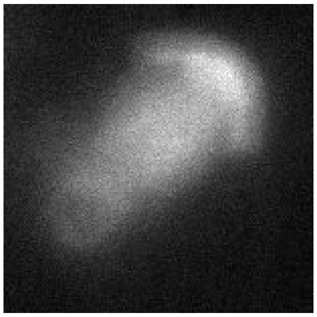

(Acrobat required for 3D)

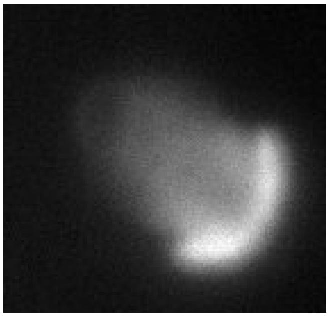

(Acrobat required for 3D)

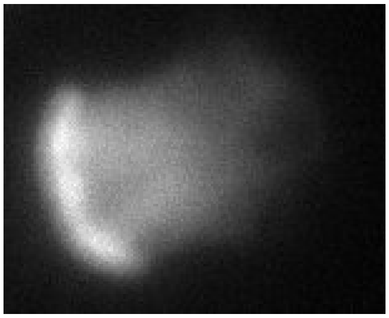

(Acrobat required for 3D)

Figure S6: 2D projections (left) and corresponding interactive 3D reconstructions (right) of unconstrained beads (15.5-μm spacers) showing bi- and tri-lobed structure. Beads are 5-μm diameter. Head-space between slide and coverslip is controlled with 15.5-μm diameter glass spacer beads mixed into the reaction. 2D projections are the confocal z-stacks summed in the z-direction. 3D reconstructions are isosurfaces at low (transparent) and high (green) density, thresholds chosen to best convey the shell morphology.

(Acrobat required for 3D)

Figure S7: Interactive 3D view of *in silico* network trajectory relative to bead during smooth motion. Network trajectory lines represent motion of an evenly distributed subset of nodes relative to the bead.

(Acrobat required for 3D)

Figure S8: Interactive 3D view of *in silico* network trajectory relative to half-coated capsule during smooth motion

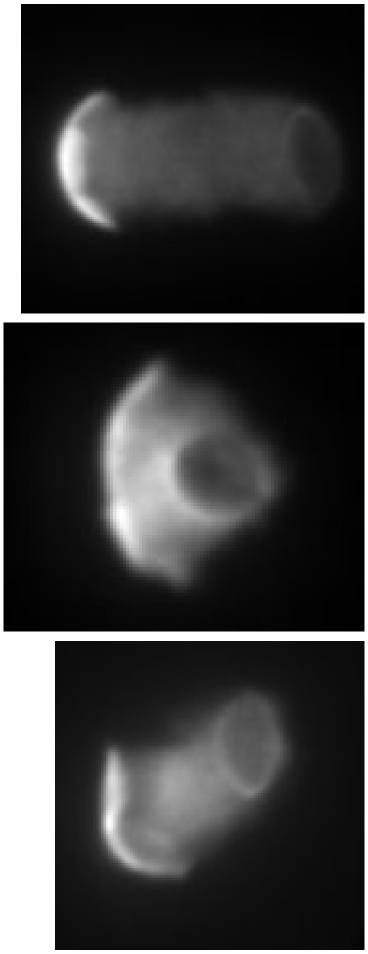

(Acrobat required for 3D)

(Acrobat required for 3D)

(Acrobat required for 3D)

Figure S9: 2D projections (left) and corresponding interactive 3D reconstructions (right) of shells and tails from unconstrained ellipsoidal beads showing sideways symmetry breaking and motility

S3 Model Robustness

To determine how the model behaviors depend on the parameters, we took our default parameter set (Section S4) and varied each parameter one by one. Figures S10 to S19 show the effect of varying the parameters, with images on the left showing timepoints during the run, and the corresponding bead velocities on the right (exact parameters are included in tiny writing in the top left of the figures). Some of the runs are cut short for some values of particular parameters (e.g. Figure S13 when high `LINK_BREAKAGE_FORCE`) because the run essentially stalls, and occasionally the algorithm that decides the camera position gets the axis wrong and the view is parallel to, rather than orthogonal to, the symmetry breaking crack axis.

Overall motility and pulsatile motion are extremely robust, but the smoothness of motion is fragile—changing many of the parameters will cause a transition to pulsatile motion.

Some particularly interesting points to note:

S3.1 Increasing Radius produces pulsatile motion

Figure S10 shows that increasing the bead radius causes a transition from smooth to pulsatile motion, mimicking that seen in experiments (Bernheim-Groswasser et al., 2002). This run was performed with bead-tail attachments turned off and not varying nucleator inertia as a function of radius to demonstrate that this transition is due to an effect of the radius of the bead on the network. At smaller bead radii, two things operate: First there the curvature is higher, so the network expansion is effectively faster (Bernheim-Groswasser et al., 2002), and secondly the ratio bead size to the network mesh size is smaller. This means that it is harder for tension to build up around the bead (through the effective mesh size of the network) because the bead can effectively go through the mesh. Another way to look at this is as analogous to reducing the probability of crosslinking `P_XLINK`. This produces smooth motion by increasing the effective mesh size when there are fewer links (few links, loose connections, larger effective mesh size) and the bead can move smoothly through the mesh. Reducing the radius does the same thing—the mesh size is the same, but now the smaller bead can move through it. Effective meshwork size is hard to control here, so it is difficult to tease out the relative contributions of these two factors on the transition to smooth motion for smaller beads. Nonetheless, the current model does have very simplified treatment of how drag on the bead changes with bead size. Since changing the nucleator inertia explicitly (Figure S18) also affects the smoothness of motion, we are cautious in interpreting the effect of changing the radius with the current model.

S3.2 Shell thickness

The shell is relatively constant thickness for all parameters except for `LINK_FORCE`, i.e. the spring constant of the network (figure S14). When the spring constant is low, the network stretches a lot before offering a significant restoring force. Since this (circumferential) stretching is the cause of the symmetry break, decreasing the spring constant increases the thickness of the shell.

S3.3 Shell Flatness

Varying `LINK_BREAKAGE_FORCE` changes the force required to break links of the network. Figure S13 shows that for very low `LINK_BREAKAGE_FORCE` the network is incoherent, similar to the network with very few links (c.f. low values of `P_XLINK`). Unlike varying `P_XLINK`, high values of `LINK_BREAKAGE_FORCE` produce a very flat shell after symmetry breaking (looking at these results in 3D show the shell indeed to be planar). This supports the model of symmetry breaking: when the `LINK_BREAKAGE_FORCE` is very high, no outer shell links break except when the shell rips right through in the catastrophic symmetry break rip. When the shell relaxes, since no links broke in the outer shell, the equilibrium area of this outer shell is still exactly the same as the inner shell, so the shell relaxes to a flat plane.

S3.4 Multiple tails

Just as high values of `LINK_BREAKAGE_FORCE` produce strong shells that become planar when they open, low values of `LINK_BREAKAGE_FORCE` produce shells that fragment, resulting in multiple tails (Figure S13).

S3.5 Pulsatile motion with no bead-network friction

Figure S20 demonstrates that increasing `P_XLINK` in the absence of any bead-network attachments still induces a transition from smooth to pulsatile motion, showing that this pulsatile motion does not require friction.

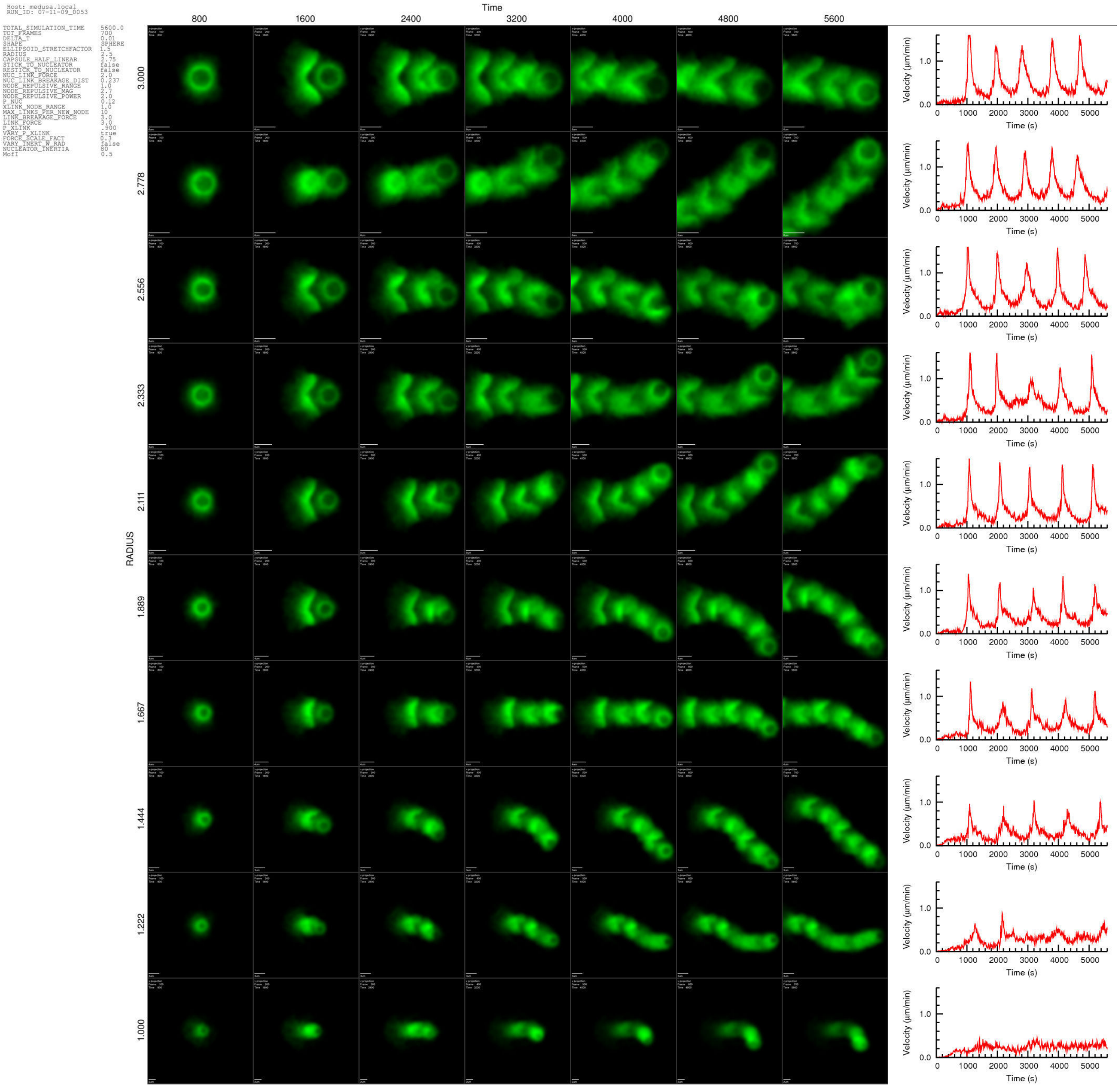

Figure S10: Effect of varying `RADIUS`. Matrix plot showing 2D projection of simulation at time points indicated for a range of parameter values. Corresponding bead velocity profiles are plotted on the right. The basis parameters are shown in the top left (zoom to view).

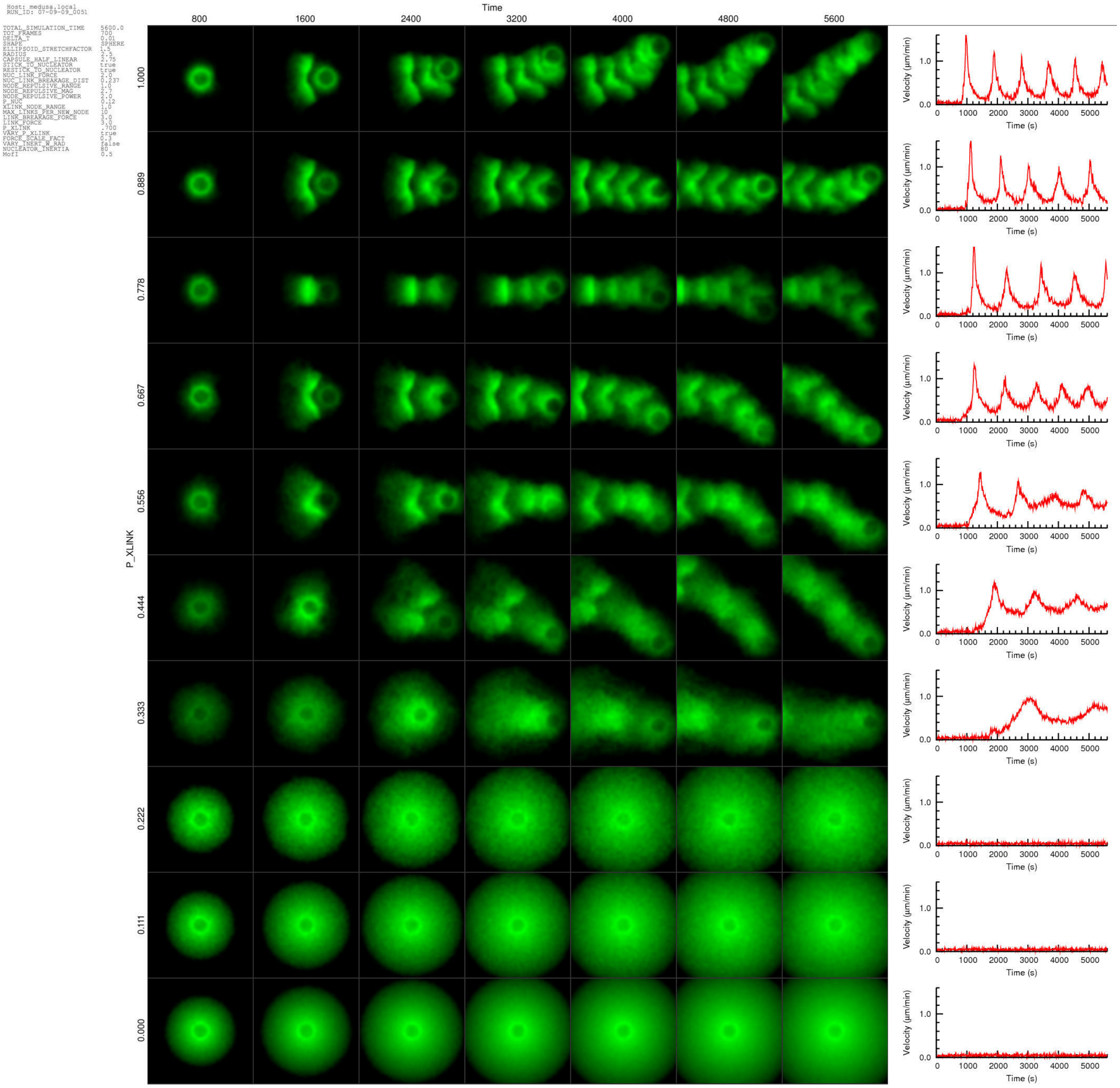

Figure S11: Effect of varying P_XLINK. Matrix plot showing 2D projection of simulation at time points indicated for a range of parameter values. Corresponding bead velocity profiles are plotted on the right. The basis parameters are shown in the top left (zoom to view).

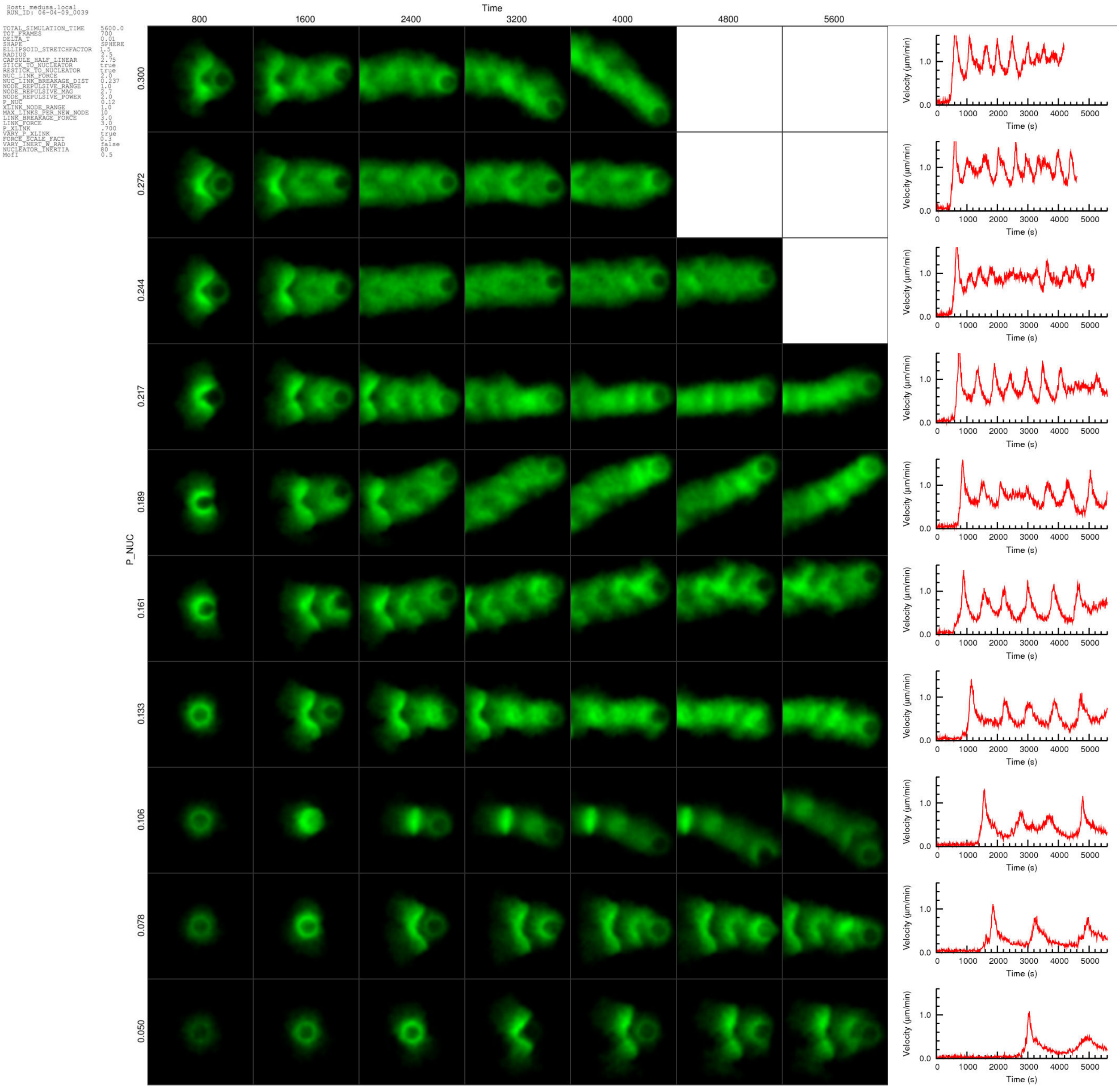

Figure S12: Effect of varying `P_NUC`. Matrix plot showing 2D projection of simulation at time points indicated for a range of parameter values. Corresponding bead velocity profiles are plotted on the right. The basis parameters are shown in the top left (zoom to view).

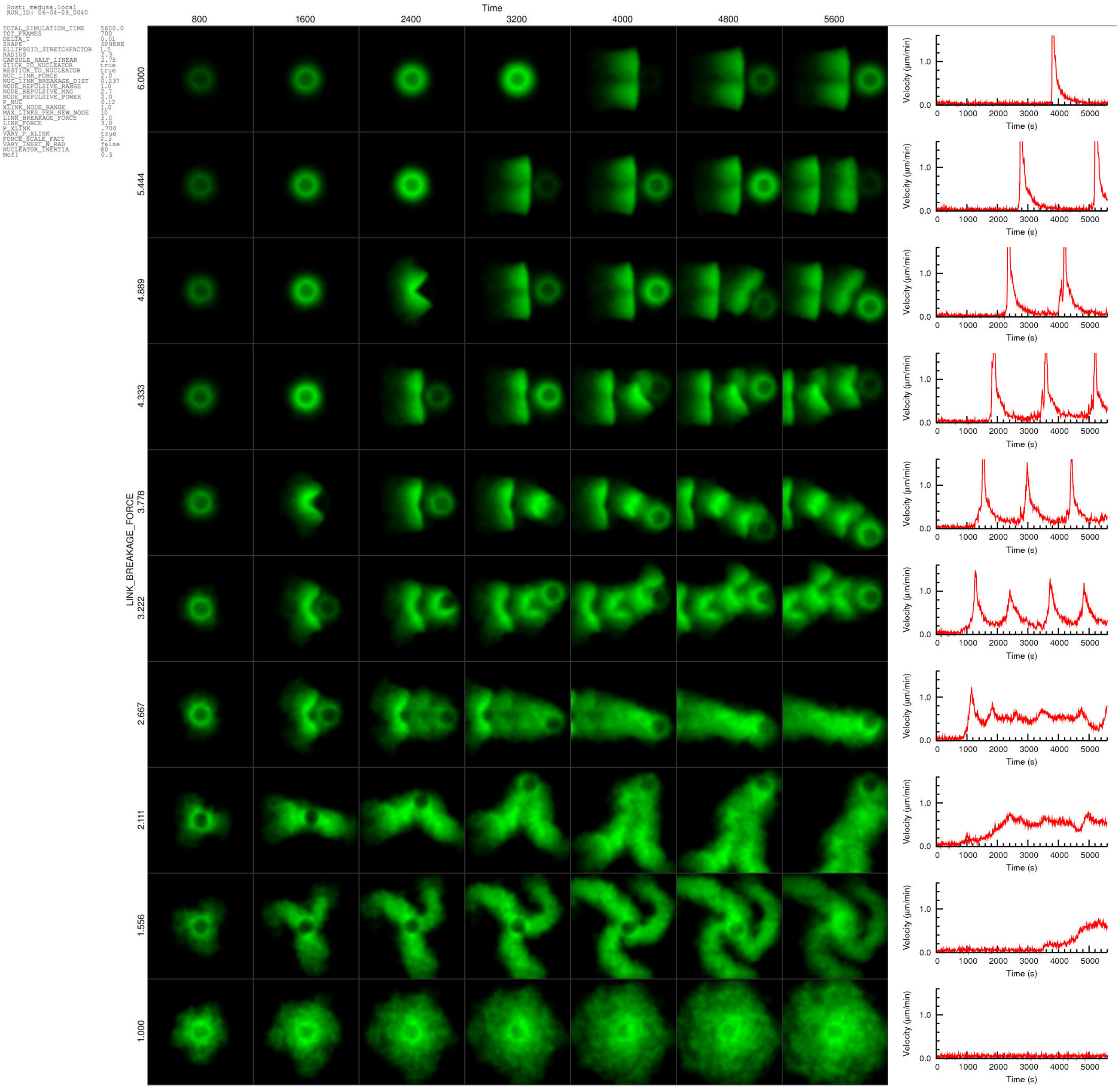

Figure S13: Effect of varying LINK_BREAKAGE_FORCE. Matrix plot showing 2D projection of simulation at time points indicated for a range of parameter values. Corresponding bead velocity profiles are plotted on the right. The basis parameters are shown in the top left (zoom to view).

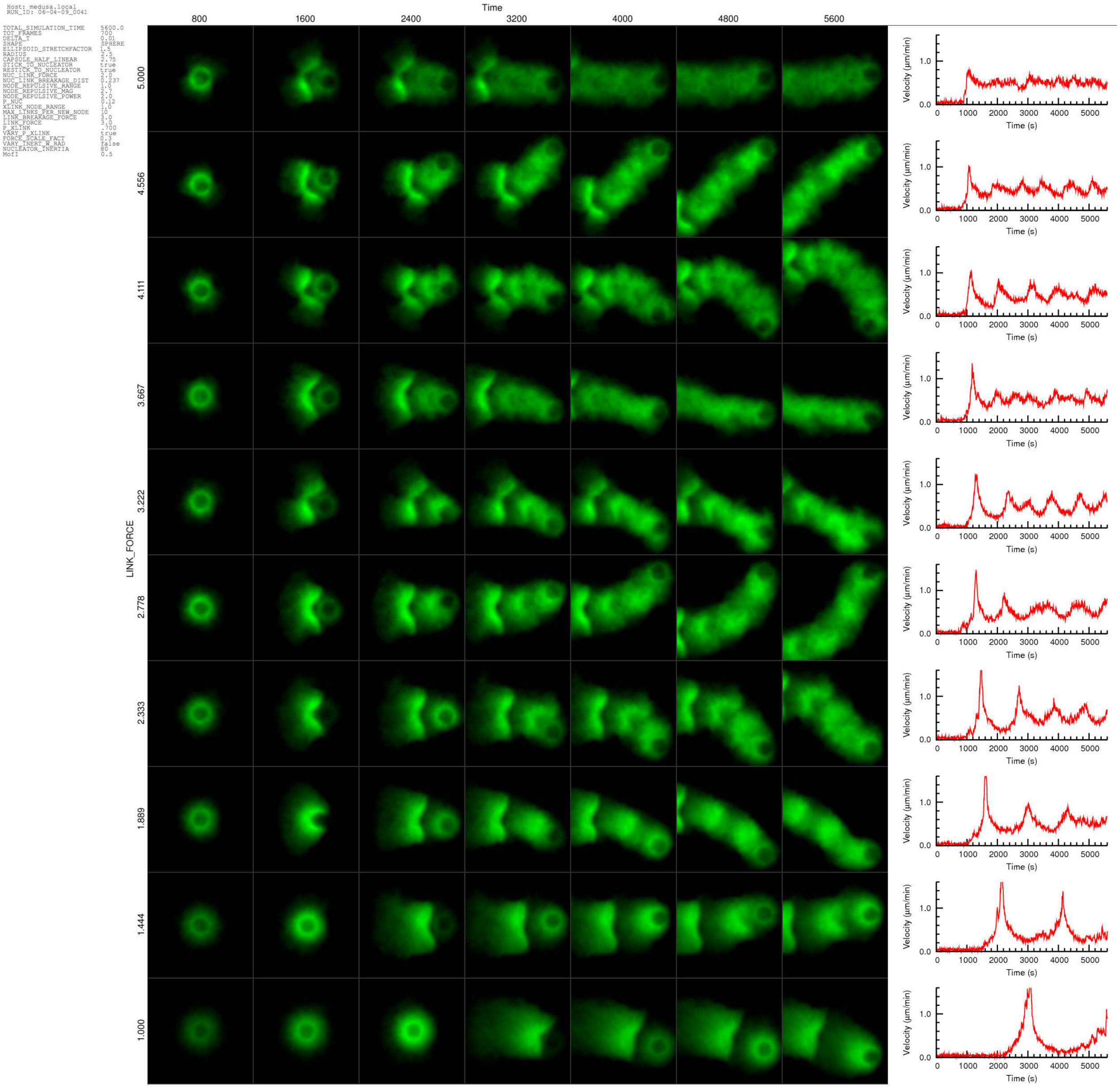

Figure S14: Effect of varying LINK_FORCE. Matrix plot showing 2D projection of simulation at time points indicated for a range of parameter values. Corresponding bead velocity profiles are plotted on the right. The basis parameters are shown in the top left (zoom to view).

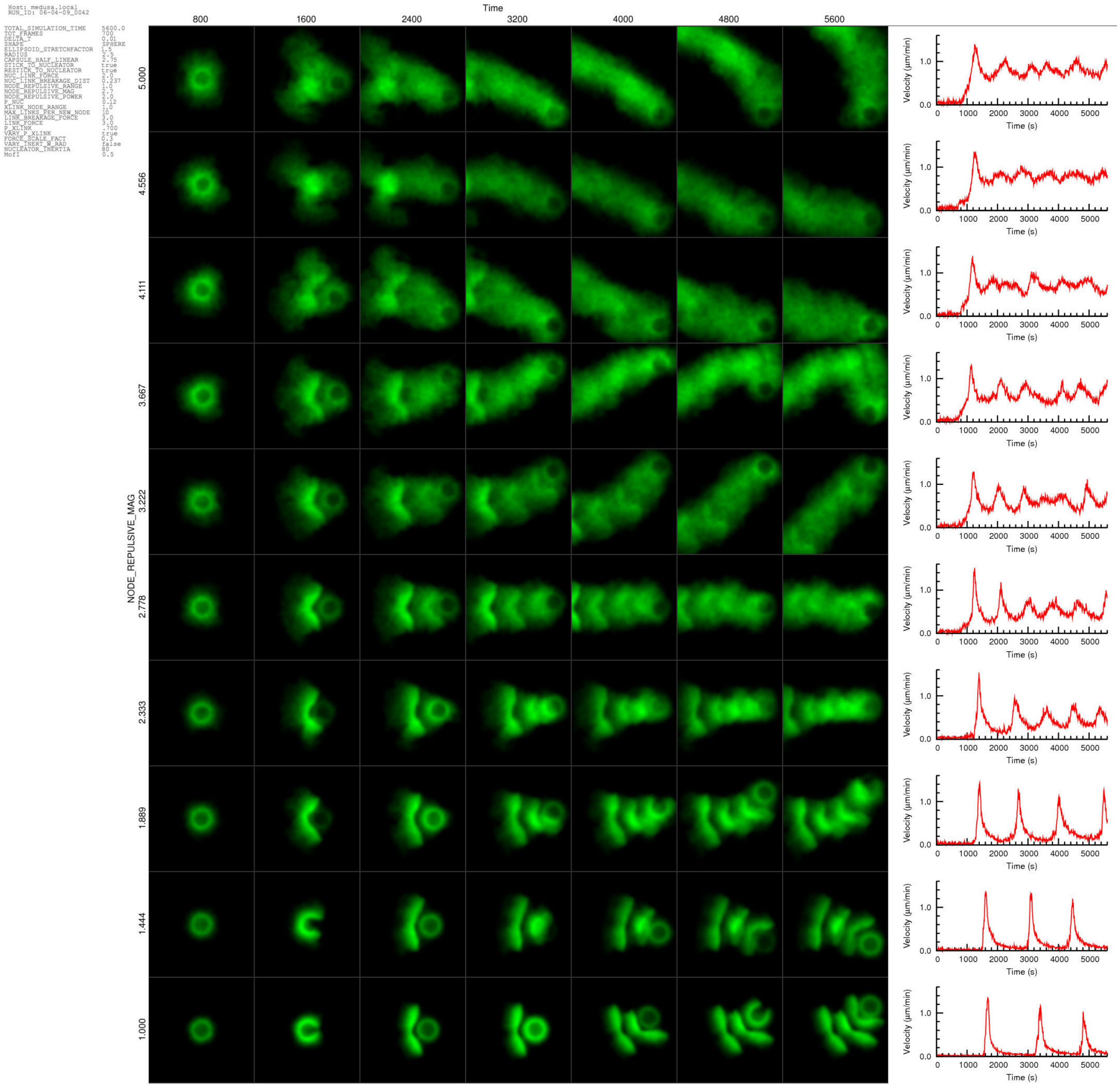

Figure S15: Effect of varying NODE_REPULSIVE_MAG. Matrix plot showing 2D projection of simulation at time points indicated for a range of parameter values. Corresponding bead velocity profiles are plotted on the right. The basis parameters are shown in the top left (zoom to view).

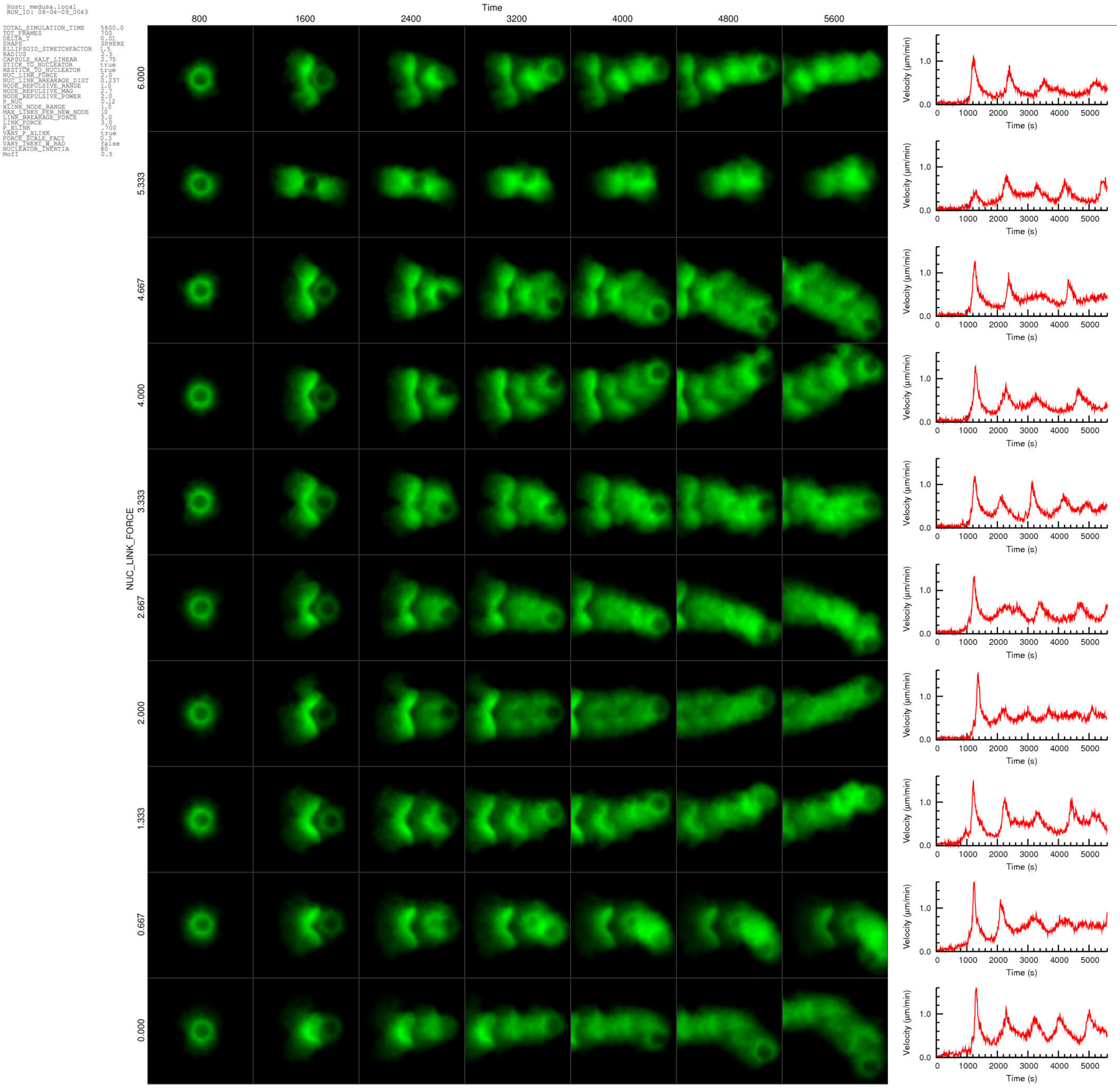

Figure S16: Effect of varying NUC_LINK_FORCE. Matrix plot showing 2D projection of simulation at time points indicated for a range of parameter values. Corresponding bead velocity profiles are plotted on the right. The basis parameters are shown in the top left (zoom to view).

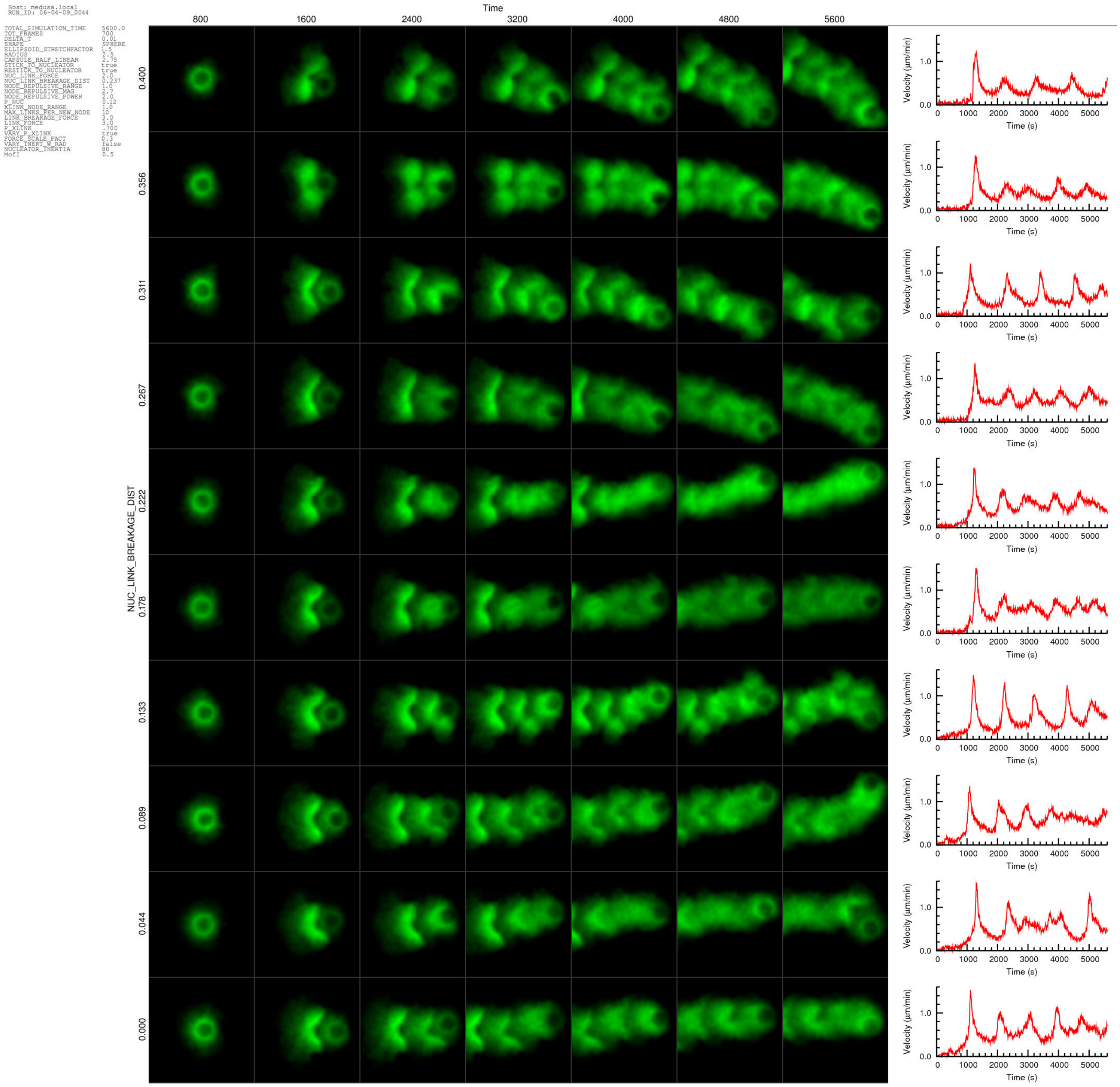

Figure S17: Effect of varying `NUC_LINK_BREAKAGE_DIST`. Matrix plot showing 2D projection of simulation at time points indicated for a range of parameter values. Corresponding bead velocity profiles are plotted on the right. The basis parameters are shown in the top left (zoom to view).

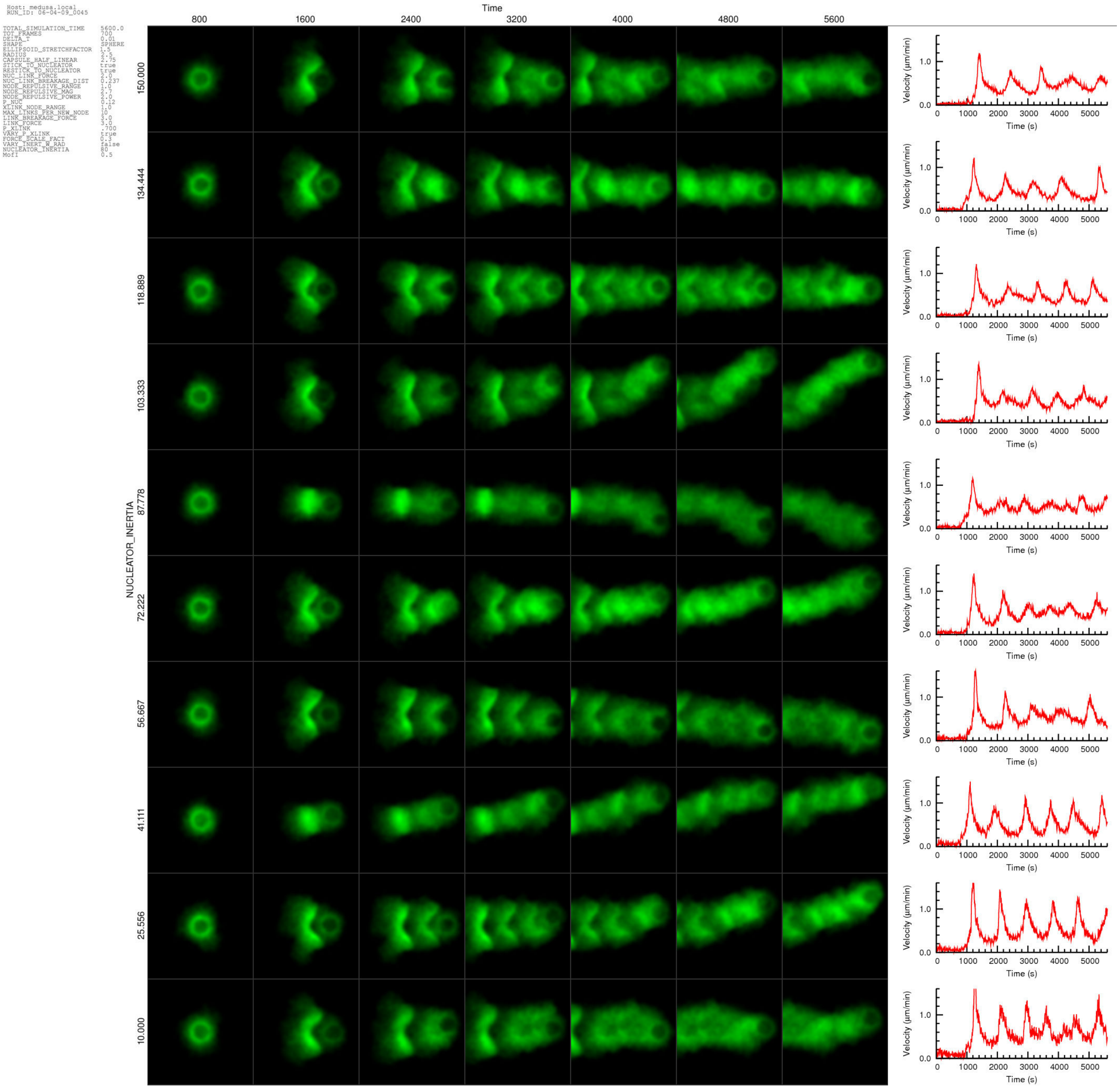

Figure S18: Effect of varying NUCLEATOR_INERTIA. Matrix plot showing 2D projection of simulation at time points indicated for a range of parameter values. Corresponding bead velocity profiles are plotted on the right. The basis parameters are shown in the top left (zoom to view).

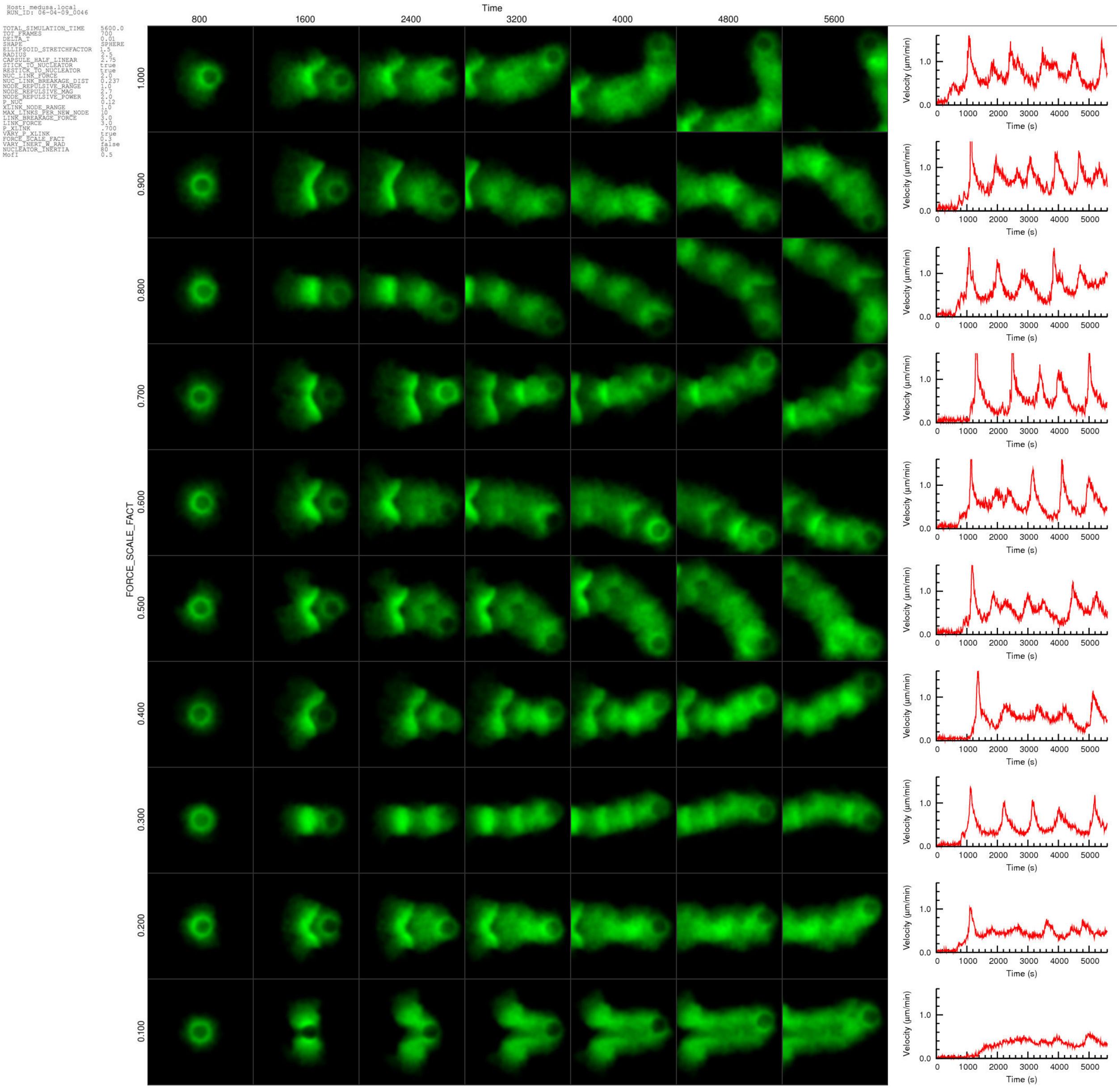

Figure S19: Effect of varying `FORCE_SCALE_FACT`. Matrix plot showing 2D projection of simulation at time points indicated for a range of parameter values. Corresponding bead velocity profiles are plotted on the right. The basis parameters are shown in the top left (zoom to view).

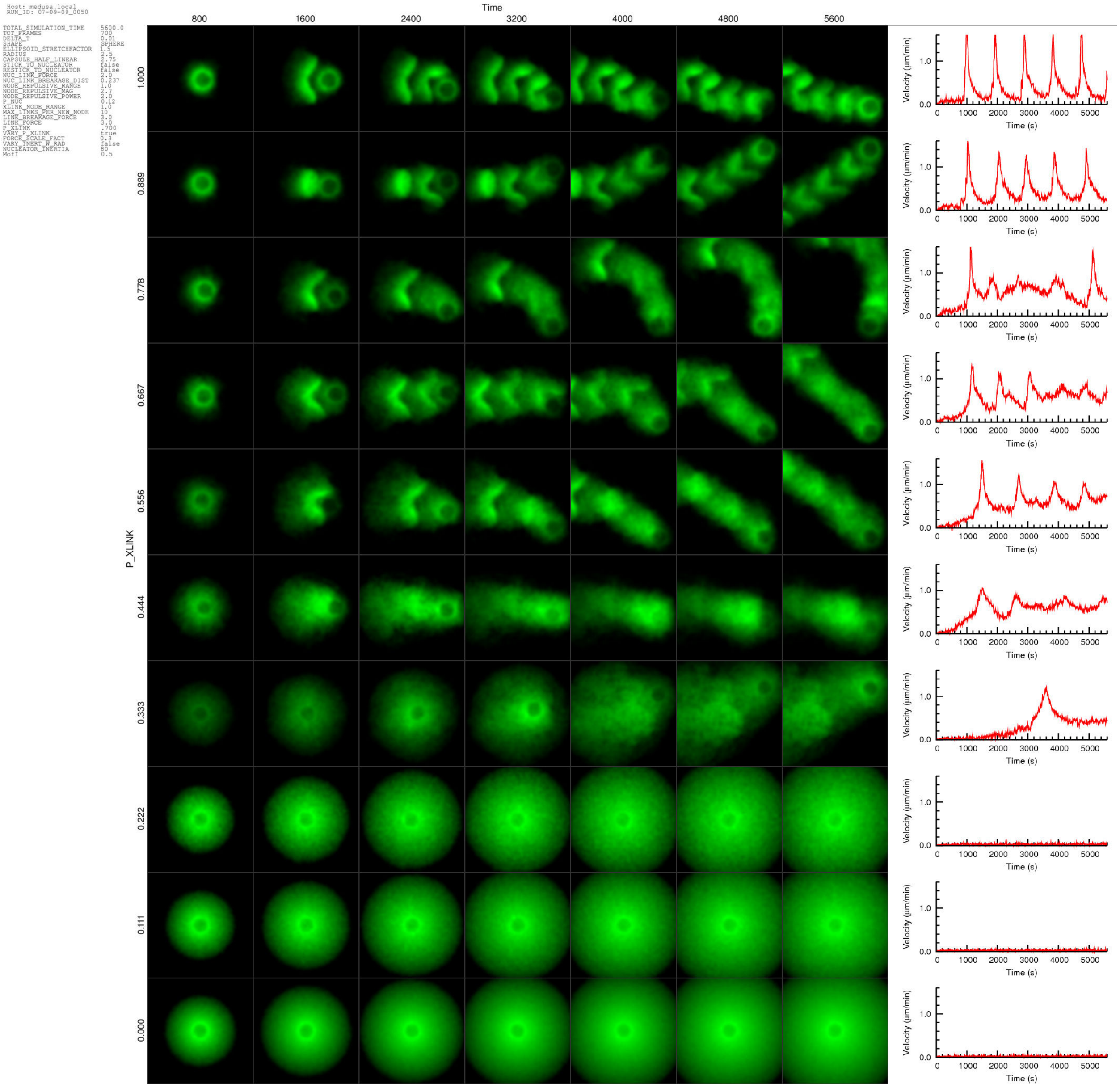

Figure S20: Effect of varying `P_XLINK` with no bead-network friction. Matrix plot showing 2D projection of simulation at time points indicated for a range of parameter values. Corresponding bead velocity profiles are plotted on the right. The basis parameters are shown in the top left (zoom to view).

S4 Simulation Settings: Model Parameters

These are the core model settings in the cometparams.ini file. The model is currently uncalibrated: units are specified in nominal microns, seconds, piconewtons etc., for purposes of understanding the parameters, but don't directly correspond to the physical properties of the actin gel.

S4.1 Run Time

```
TOTAL_SIMULATION_TIME 5600.0  # (s) how long  to run the simulation for (in simulation-time seconds)
TOT_FRAMES             700    # (frames) how many frames in this time
DELTA_T                  0.01 # (s) iteration timestep
```

TOTAL_SIMULATION_TIME defines the run length in simulation time (uncalibrated, nominally seconds). TOT_FRAMES defines the number of frames to be taken during the run, i.e. 700 frames would mean one frame every 800 iterations. Frames are not time units, but simply represent equal-spaced points in time when data is saved, bitmaps calculated, etc. DELTA_T defines the time step between iterations, i.e. for the given TOTAL_SIMULATION_TIME of 5600 and DELTA_T of 0.01, there will be a total of 560000 iterations. Increasing DELTA_T makes the run faster, but risks errors. Generally, a too-large DELTA_T will result in warnings that nodes are entering significant distances into the nucleator before being ejected, but also check that the network behavior is not being affected by artifacts of too large a DELTA_T by reducing DELTA_T by a factor of 2 or more to see if you get the same result)

S4.2 Nucleator Geometry

```
SHAPE                    SPHERE  # (SPHERE, ELLIPSOID or CAPSULE) Nuleator shape
ELLIPSOID_STRETCHFACTOR    1.5   # (unitless) Ratio of major to minor ellipse axes
RADIUS                     2.5   # (um) Radius of sphere, minor axis of ellipse, radius of
                                 #      capsule
CAPSULE_HALF_LINEAR        2.75  # (um) Half the length of the linear section for capsule
```

SHAPE can be SPHERE, CAPSULE or ELLIPSOID. For SPHERE, only the RADIUS matters. For CAPSULE, RADIUS and CAPSULE_HALF_LINEAR are used, and for ELLIPSOID, RADIUS and ELLIPSOID_STRETCHFACTOR define the shape. (Arbitrary shapes can be defined in the code, given a function that for a supplied point, returns a vector normal to the nearest point on the surface to the given point.)

S4.3 Nucleator Attachments

```
STICK_TO_NUCLEATOR      true # (boolean) whether nodes stick to nucleator upon creation
RESTICK_TO_NUCLEATOR    true # (boolean) whether nodes stick to nucleator upon contact
NUC_LINK_FORCE          2.0  # (pN/um) nucleator-node link force scaling factor
NUC_LINK_BREAKAGE_DIST 0.237 # (um) maximum nucleator-node link length before it breaks
```

When nodes are created, STICK_TO_NUCLEATOR defines whether they stick to their point of creation on the nucleator surface. Stuck nodes exert a force proportional to NUC_LINK_FORCE multiplied by the distance from the surface stuck point until they are extended beyond NUC_LINK_BREAKAGE_DIST when the link breaks. If RESTICK_TO_NUCLEATOR is true, *unstuck* nodes will re-stick if they come into contact with the surface again.

S4.4 Node-node repulsion

```
NODE_REPULSIVE_RANGE  1.0  # (um) how far to calculate the node-node repulsion function
NODE_REPULSIVE_MAG    2.7  # (pN)magnitude scale factor for repulsive force
NODE_REPULSIVE_POWER  2.0  # (unitless) power of repulsion function (see equation) (always set
                           #    to 2 for the moment)
```

The repulsion force between nodes is of the form:

$$F_R = M_R\left(\left(\frac{d_R}{d}\right)^{P_R} - 1\right), \quad 0 < d < d_R$$

where d is the distance between nodes, M_R (NODE_REPULSIVE_MAG) is a magnitude scale factor, and d_R (NODE_REPULSIVE_RAN is maximum range of the repulsive force. The power factor P_R (NODE_REPULSIVE_POWER) is 2, so this is a simple inverse square repulsive force.

S4.5 Node links

```
P_NUC                   0.12 # (nodes.um^2.s^-1) Nucleation rate (probability of forming nodes)
XLINK_NODE_RANGE        1.0  # (um) maximum distance to link two nodes when they form
MAX_LINKS_PER_NEW_NODE 10    # (links) cap the max number of links for a new node
LINK_BREAKAGE_FORCE     3.0  # (pN) maximum node-node link force before it breaks
LINK_FORCE              3.0  # (pN) magnitude scale factor for link force
P_XLINK                .700  # (unitless) Max probability (at d=0) of forming a crosslink
                             #    to a neighboring node
VARY_P_XLINK           true  # (boolean) whether to reduce probability of crosslinking
                             #    linearly with distance
```

P_NUC defines the rate of nucleation of new nodes per unit area per unit time. i.e. for one iteration, the number of new nodes added over the whole of the nucleator surface is P_NUC $*$ DELTA_T $*$ surf_area, where surf_area is in μm ^{2} . The nodes are added at random positions on the surface, with an even distribution unless the ASYMMETRIC_NUCLEATION variable is set.

New nodes are crosslinked to nearby nodes within XLINK_NODE_RANGE. The links then behave as Hookean springs, exerting a restoring force

$$F_L = -M_L\left(\frac{d-d_L}{d_L}\right)$$

where d is the distance between nodes, M_L is a magnitude scale factor, and d_L is the original length of the link when it was formed (extbackslashfref{fig:simulationdetails}). If the link is extended so that its force goes beyond a certain limit, the link breaks. (optionally this can be strain rather than stress, i.e. a break occurs when $\frac{d}{d_L}$ exceeds a certain limit rather than when $\frac{d-d_L}{d_L}$ does)

Nodes are added to the surface and fixed there while their repulsive forces are ramped up linearly from 0 to full. This allows time for nodes already at the surface move and make room for the new node before it is crosslinked. The ramp-up occurs over CROSSLINKDELAY iterations. MAX_LINKS_PER_NEW_NODE limits the maximum number of crosslinks for each new node. LINK_FORCE is the spring constant, and when the extension forces reaches LINK_BREAKAGE_FORCE, the link breaks. P_XLINK is the probability of forming a crosslink to a node within range (still restricted by the MAX_LINKS_PER_NEW_NODE limit). The VARY_P_XLINK flag (normally on) also imposes a linear tail-off of this probability with distance.

S4.6 Drag

```
FORCE_SCALE_FACT      0.3 # (um.pN^-1.s^-1) how fast nodes move for a given force
VARY_INERT_W_RAD    false # (boolean) whether to vary nucleator inertia with radius
NUCLEATOR_INERTIA      80 # (unitless, or um^-1)  Scale factor for how much harder
                          #     it is to move nucleator than nodes
MofI                  0.5 # (rad.um^1.^pn) How hard it is to rotate the nucleator
```

This section relates the forces to the actual movement of the nodes and nucleator. FORCE_SCALE_FACT scales the movement of nodes (i.e. effectively inverse of node drag). If you reduce this, you probably need to reduce DELTA_T as well. NUCLEATOR_INERTIA determines how hard it is to *displace* the nucleator and MofI determines how hard it is to rotate it. If VARY_INERT_W_RAD is set, inertia will be scaled by the size of the nucleator.

S5 Simulation Settings: Display

These settings in the `cometparams.ini` affect the way data is displayed. There are two display outputs, bitmaps (2D) and VTK (3D). Bitmaps are automatically created during the initial calculation run, but usually start only after the symmetry breaking direction is determined (since this determines the observer position). Bitmaps can also be produced after the initial calculation run is complete with `comet post`, and VTK output is produced only after the initial calculation run with `comet view` or `comet vtk`:

S5.1 Producing Bitmaps

You can call the bitmap processing with

```
comet post 0:0
```

where `0:0` processes all frames, or a range of frames if specified.

S5.2 Producing VTK output

You can call the VTK processing either interactively with

```
comet view 300:300
```

Note only a single frame can be specified for the interactive view.
or in batch mode with

```
comet vtk 0:0
```

VTK can also produce vrml files to import the 3D models into other software (e.g. Acrobat 3D) by calling with one frame only

```
comet vtk 300:300
```

S5.3 Bitmap display settings

S5.3.1 Basic settings

```
X_BMP                   true       # whether to write a bitmap for x axis (this is the
                                   #    default for the symmetry breaking plane)
Y_BMP                   true       # whether to write a bitmap for y axis
Z_BMP                   true       # whether to write a bitmap for z axis
WRITE_BMPS_PRE_SYMBREAK false      # whether to write images before symmetry breaks.
                                   #      (mainly useful to see what is going on for
                                   #       conditions when symmetry doesn't break)
BMP_WIDTH               800        # width of bitmap in pixels
BMP_HEIGHT              800        # height of bitmap in pixels
VIEW_HEIGHT             30         # bitmap scale (height of image in um)
BMP_OUTPUT_FILETYPE     jpeg       # graphic type for bitmap save (must be recognized
                                   #      ImageMagick type)
BMP_COMPRESSION         100        # bitmap quality setting (ImageMagick)
DRAW_CAGE               false      # whether to draw the nucleator on the bitmaps
                                   #      (as a 2D projection of cage of points)
CAGE_ON_SIDE            false      # whether to draw the cage on the side of the image
GAUSSFWHM               0.70       # width of the gaussian used to blur the node
                                   #      points to make the pseudo microscope image
INIT_R_GAIN             80         # initial gain for red bitmap channel
                                   #       (rescaled at symmetry breaking)
INIT_G_GAIN             30         # initial gain for green bitmap channel
INIT_B_GAIN             200        # initial gain for blue bitmap channel
BMP_AA_FACTOR           1          # antialiasing factor (produces bigger image and resizes)
```

S5.3.2 Plotting forces on bead

```
SEGMENT_BINS              false       # whether to plot radial segments
RADIAL_SEGMENTS           12          # number of radial segments
PLOTFORCES                false       # whether to plot forces
PLOTFORCES_INCLUDEIMPACTS true        # whether to include surface impacts in
                                      #     force display vectors
PLOTFORCES_INCLUDELINKFORCES true     # whether to include link tension in force
                                      #     display vectors
FORCE_BAR_SCALE           10          # scale factor for force plotting
```

S5.3.3 Plotting speckle in shell and tail

```
SPECKLE                   true        # whether to color actin with speckles
SPECKLEGRID               true        # specles as grid?
SPECKLEGRIDPERIOD         1000        # grid period (time)
SPECKLEGRIDTIMEWIDTH      0           # grid stripe pulse width (time)
SPECKLEGRIDSTRIPEWIDTH    0.3         # grid bar width (distance)
SPECKLE_FACTOR            0.3         # density of speckles if no grid
```

S5.4 VTK settings (3D)

S5.4.1 Basic Settings

```
VTK_WIDTH                 800         # VTK image width
VTK_HEIGHT                800         # VTK image height
VIS_PROJECTION            z           # position of camera: x,y,z or rip
                                      #      x,y,z correspond to the bitmap images,
                                      #      rip puts the camera ahead of and slightly
                                      #      above bead to view the rip
VTK_AA_FACTOR             2           # antialias factor
COLOUR_GAMMA              1.6         # color scale gamma
```

S5.4.2 What to display

```
VIS_NUCLEATOR             true        # whether to display nucleator
VTK_NUC_WIREFRAME         true        # whether to display wireframe nucleator in
                                      #     addition (helps show rotation)
VIS_NODES                 false       # whether to display individual nodes as balls
VIS_LINKS                 false       # whether to display links as lines
VIS_SHADELINKS            true        # whether to color links by strain
VIS_ISONODES              false       # whether to display isosurfaces of node density
VIS_NUCOPACITY            1.0         # opacity of nucleator
VIS_TRACKS                true        # whether to display node tracks
VIS_USENUCTEXMAP          false       # whether to put texture on nucleator
```

S5.4.3 3D view settings

```
VTK_MOVE_WITH_BEAD        false       # whether to keep bead in center of screen
VIS_LINETHICKNESS         1.2         # line thickness for links
VIS_PSCALE                55          # scaling factor
VTK_VIEWANGLE             50          # camera zoom
VIS_PARALLELPROJECTION    true        # turns off perspective
VIS_CAMERADISTMULT        5           # how far to put camera (multiple of radius)
VIS_NORMALISEFRAMES       false       # whether to normalize intensity of node
                                      #     density isosurface
```

S5.5 Misc

```
VIS_VTK_HIGHQUAL        false       # VTK antialiasing
VIS_FILEPREFIX          vtk         # output file prefix
```

S5.6 Settings that apply to both bitmaps and VTK

```
SYM_BREAK_TO_RIGHT true             # rotate camera to orient symmetry break direction
                                    #       to the right (else just rotate to be
                                    #       in the y-z plane)
FOCALDEPTH 2.5                      # restrict plotting of nodes etc. to slab twice
                                    #       this distance thick centered on bead
BMP_FIX_BEAD_MOVEMENT false         # move camera with bead so bead stays in center of screen
BMP_FIX_BEAD_ROTATION false         # rotate the camera with the bead
```

S6 Installing the program

Note: Please also check online at http://www.dayel.com/comet for up-to-date instructions.

S6.1 Precomiled binary for OS X

We provide a precompiled binary for Mac OS X, and instructions for compiling from source for OS X, Linux and Windows. The precompiled OS X binary includes the VTK 3D visualization and GSL random number generator, but still requires ImageMagick for the bitmap conversion.

S6.2 Compiling from source

S6.2.1 Downloading the source

The source is freely available via http://www.dayel.com/comet under the open source GNU General Public License. Makefiles are included for building with GCC on linux, OS X, windows, and DEC Alpha, and an Xcode project file is also included for OS X. (Note: the default configuration is dependent on VTK and GSL libraries (see below).)

S6.3 Dependencies

The code has two optional dependencies, the Gnu Scientific Library (GSL) which provides the Mersenne Twister random number generator (more statistically valid than the standard rand() function), and The Visualization Toolkit (VTK) which provides the 3D visualization routines. If these libraries are not available, you can compile without them by changing the #define's USE_GSL_RANDOM and LINK_VTK in the file stdafx.h from 1 to 0 respectively, and removing their mention from the makefiles.

S6.4 Platform Specific Information

The code has been developed on OS X 10.5 and Linux, but should run fine in windows under cygwin.

S6.4.1 OS X

First install the Apple Developer Tools, then open the Xcode project file included in the source. Include the GSL and VTK libraries in the search path, or disable before compiling (see above).

S6.4.2 Linux

A makefile is included for compilation with GNU Make. This should be edited to point to the GSL and VTK libraries, or disable them before compiling (see above).

S6.4.3 Windows

First install cygwin, then use cygwin to install ImageMagick, bzip2 and gcc, then compile as for Linux. If compiling with Visual Studio instead of gcc, you will also need a pthreads library.

S7 Running the program

S7.1 Runtime prerequisites

The program requires ImageMagick for writing images and bzip2 for compressing data files. We recommenced using macports to install ImageMagick on OS X, and cygwin to install ImageMagick and bzip2 on windows (see 'Platform Specific Information' below).

S7.2 Command line syntax

The program expects to be run from a new directory containing a copy of the control file `cometparams.ini`, an example of which is included in the source code and explained in detail below. Typing '`comet`' without any parameters returns the command line syntax:

For a new simulation setup the parameter file 'cometparams.ini' in current directory and type: `comet <numThreads>` where `<numThreads>` is the number of CPUs to use e.g. typing '`comet 4`' will start a new run using 4 simultaneous threads and parameters read from the `cometparams.ini` control file.

To process an existing dataset type: `comet <command> <frame range>` where `<command>` is 'post' to write bitmap images, 'vtk' to write 3D images or 'view' to enter 3D interactive mode. e.g `comet post 1:300` writes bitmaps for frame 1–300, `comet view 300:300` enters 3D interactive mode for frame 300 (the range '0:0' can be used to process all frames).

S7.3 Running the program on a cluster

We highly recommend running 'comet' on a cluster of machines rather than just one. The program takes some time to run (an hour or two for early events like symmetry breaking, or overnight if you want to look at the later motility, pulsatile motion etc.) Running the program concurrently on at least 5 machines lets you efficiently test the effect of changing one parameter through a range of values. You can set it going then come back the following day and have the whole thing laid out for you. We have found this very useful, and have included a set of scripts to automate the process, including automatically generating the montage of images seen in the robustness section so you can quickly scan the effect of varying the parameter.

We recommend putting a default **cometparams.ini** into a main directory for the data e.g. **/runs**. In that directory run the **varyset** script (included with the source):

```
varyset <parameter> <startval> <endval> <number of steps>
```

This will create a subdirectory within **runs** that contains subdirectories numbered 1,2,3,etc. each containing a version of the **cometparams.ini** file with the <parameter> value varying in linear steps between <startval> and <endval>. It will also add information to run the individual comet jobs into `~/joblist`.

If you have access to a cluster with a working job control system, you might want to use that. We had trouble with the job control system on the cluster we were using, and ended up writing our own:

On the head node, we have the

```
startnewjobs
```

script running as a cron job. This checks to see if the worker nodes are idle (5 min load average below a certain threshold) and starts the next job if they are.

If you don't have access to a cluster, there is a single computer version of this

```
startjobsloop
```

which will check /joblist for new jobs and run them sequentially.

The script

```
makematrix
```

pulls together an image matrix (as seen in the robustness section) to summarize the effect of varying the parameter. The directory name, time, computer and main section of the competparams.ini file are converted into an image and included on the left hand side of the summary, to keep track of the details of the run.

S8 Model Implementation

S8.1 Program Flow

Figure S21 shows the detailed program flow. The main iteration loop is in blue, the post-processing is in green, and the frame processing is in pink.

S8.2 Implementation in C++

The code is written in C++ for speed. We attempt to use an somewhat object-based approach, but a good many of the member variables are declared as static global to allow their access across threads.

Here is a breakdown of the main classes and functions in the program. There are numerous other functions but this is the core of the progra (also see Figure S21). This is intended as an overview for programmers before working on the code itself.

- Main()
  - Spawns threads: `collisiondetectionthread` , `linkforcesthread` and `applyforcesthread` depending on the `USETHREAD_COLLISION` , `USETHREAD_LINKFORCES` and `USETHREAD_APPLYFORCES` parameters.
  - Parses the `comet_params.ini` file to read parameters. All of the parameters are implemented as globals (should fix at some point)
  - Creates the main `theactin` and `nuc_object` objects.
  - Runs through the main iteration loop, calling `theactin.iterate()` and saving snapshots every so often.
- Actin class
  - There is only one actin object, `theactin` , which constitutes the network, i.e. contains the nodes and the functions that deal with them.
  - The `iterate()` function does one iteration pass, calling:
    * `nucleator_node_interactions()` displaces any nodes out of the nucleator object along a normal to the nucleator surface
    * `nucleate()` adds new harbinger nodes to the surface of the nucleator
    * `crosslinknewnodes()` crosslinks harbingers once they are ready
    * `sortnodesbygridpoint()` orders nodes by gridpoint. The { extbackslashit only} reason for this is for the division of labor when using threads: We do repulsion by gridpoint to save re-calculating nearby nodes if there are multiple nodes on one gridpoint, and we do not want to divide nodes on one gridpoint across multiple threads.
    * `collisiondetection()` detects whether nodes are within `NODE_REPULSIVE_RANGE` of one another and adds the repulsive force to `rep_force_vec[]` .
    * `linkforces()` Calculates the forces between nodes due to links and puts into `link_force_vec[]` . If a link goes above a certain threshold force, marks it as broken and removes next time (again to prevent thread problems—since a link is removed both ways and we can't guarantee that both nodes are being processed by same thread)
    * `applyforces()` updates the positions of all the nodes. Sums over the threads for `rep_force_vec[]` , `link_force_vec[]` and `repulsion_displacement_vec[]` .
    * Numerous other functions for things like saving bmps, vrml etc.
  - Nucleator class
    * There is only one nucleator object at the moment, `nuc_object` , which is closely linked to the actin object
    * The nucleator is either a sphere, a capsule (i.e. a sphere with a cylindrical segment stuck in the middle) or ellipsoid

    * `addnodes()` adds harbingers to the surface of the nucleator. The probablility of addition of nodes is normalized by surface area and is symmetric if `ASYMMETRIC_NUCLEATION` is zero, or asymmetric if 1 or 2 (stepped or linear bias)
    * `definenucleatorgrid()` sets a list of gridpoints to check in case of nodes entering the nucleator. Called once at the beginning.
    * `iswithinnucleator()` returns true if the node is within the nucleator
    * `collision()` moves a node out of the nucleator along a normal vector

- Nodes class
    * Nodes exist only as members of the actin object
    * `nodegrid` is a 3 dimensional C++ vector of node pointers. Each nodegrid entry starts a circularly linked list of nodes representing the nodes within that gridpoint voxel.
    * The actin class contains a vector of nodes. Each node has an associated `nodenum` , `x` `y` and `z` position, `nextnode` and `prevnode` node pointers for the nodegrid linked list, `rep_force_vec[]` , `link_force_vec[]` and `repulsion_displacement_vec[]` as described above, the grid position of the node, `harbinger` and `polymer` flags and a `listoflinks` i.e. a vector of link object which attach this node to other nodes.
    * `polymerize()` Creates a node as a harbinger. Adds its pointer to the gridpoint linked list.
    * `depolymerize()` Removes a node, deletes all links and removes from grid.
    * `setgridcoords()` Calculates new grid co-ordinates based on x,y,z position
    * `addtogrid()` adds the node to the current gridpoint
    * `removefromgrid()` removes node from the grid
    * `updategrid()` checks to see if node has moved gridpoints, and updates grid is needs to
    * `removelink()` removes the specified node from the list of links

- Links class
    * Links exist only as members of the node objects
    * Each link has an associated `linkednodeptr` which points to the target node that the link is to and a `broken` flag which is read by `actin::linkforces()` and tells it to delete the link if it broke.
    * `orig_dist` and `orig_distsqr` store the original distance of the link
    * `breakcount` stores the number of consecutive iterations the link force has been above `LINK_BREAKAGE_FORCE` and is used to increase the probability of breakage
    * `getlinkforces()` returns the force acting on the link. Also sets the `broken` flag and increments `breakcount` if appropriate

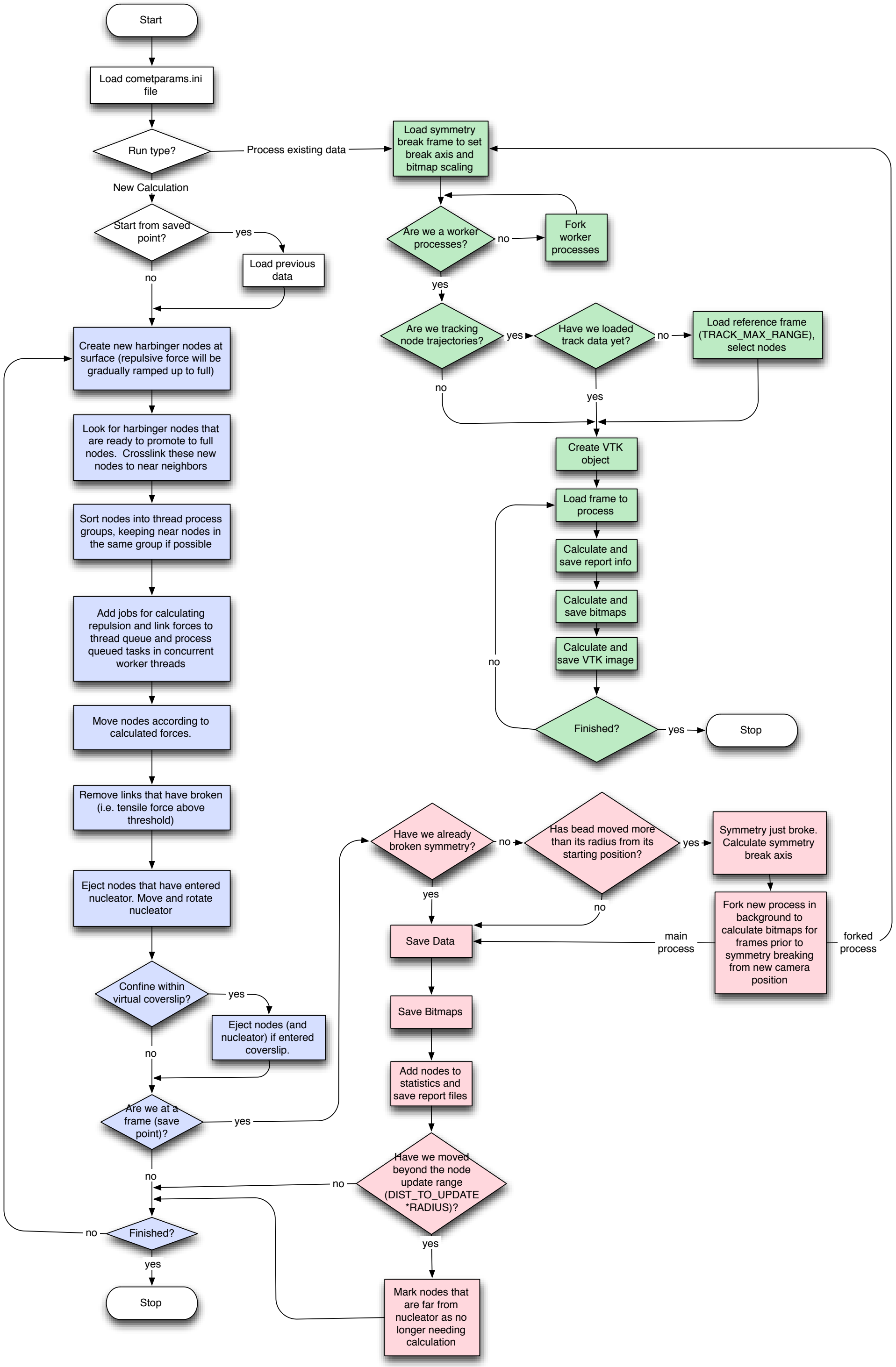

Figure S21: Detailed program flow

References

Akin, O. and Mullins, R. D. (2008). Capping protein increases the rate of actin-based motility by promoting filament nucleation by the arp2/3 complex. *Cell*, 133(5):841–51. Akin, Orkun Mullins, R Dyche PN2 EY016546-04/EY/United States NEI R01 GM061010-09/GM/United States NIGMS R01GM61010/GM/United States NIGMS United States Howard Hughes Medical Institute Research Support, N.I.H., Extramural Research Support, Non-U.S. Gov't United States Cell Cell. 2008 May 30;133(5):841-51.

Bernheim-Groswasser, A., Wiesner, S., Golsteyn, R. M., Carlier, M. F., and Sykes, C. (2002). The dynamics of actin-based motility depend on surface parameters. *Nature*, 417(6886):308–11. Bernheim-Groswasser, Anne Wiesner, Sebastian Golsteyn, Roy M Carlier, Marie-France Sykes, Cecile England Nature Nature. 2002 May 16;417(6886):308-11.

Bird, R. B., Armstrong, R. C., and Hassager, O. (1977). *Dynamics of polymeric liquids*. Wiley, New York ; London. R. Byron Bird, Robert C. Armstrong, Ole Hassager. 26cm. Vol.2 by R. Byron Bird and others.

Boal, D. H. (2001). *Mechanics of the cell*. Cambridge University Press, Cambridge. GBA202813 bnb 2673 David H. Boal. ill. ; 26 cm. Includes bibliographical references and index.

Bottino, D. C. and Fauci, L. J. (1998). A computational model of ameboid deformation and locomotion. *Eur Biophys J*, 27(5):532–9. Bottino, D C Fauci, L J Comparative Study Research Support, U.S. Gov't, Non-P.H.S. Germany European biophysics journal : EBJ Eur Biophys J. 1998;27(5):532-9.

Delatour, V., Shekhar, S., Reymann, A.-C., Didry, D., Le, K. H. D., Romet-Lemonne, G., Helfer, E., and Carlier, M.-F. (2008). Actin-based propulsion of functionalized hard versus fluid spherical objects. *New Journal of Physics*, 10(2):025001.

Footer, M. J., Kerssemakers, J. W., Theriot, J. A., and Dogterom, M. (2007). Direct measurement of force generation by actin filament polymerization using an optical trap. *Proc Natl Acad Sci U S A*, 104(7):2181–6. Footer, Matthew J Kerssemakers, Jacob W J Theriot, Julie A Dogterom, Marileen Research Support, Non-U.S. Gov't United States Proceedings of the National Academy of Sciences of the United States of America Proc Natl Acad Sci U S A. 2007 Feb 13;104(7):2181-6. Epub 2007 Feb 2.

Fujiwara, I., Suetsugu, S., Uemura, S., Takenawa, T., and Ishiwata, S. (2002). Visualization and force measurement of branching by arp2/3 complex and n-wasp in actin filament. *Biochem Biophys Res Commun*, 293(5):1550–5. Fujiwara, Ikuko Suetsugu, Shiro Uemura, Sotaro Takenawa, Tadaomi Ishiwata, Shin'ichi Research Support, Non-U.S. Gov't United States Biochemical and biophysical research communications Biochem Biophys Res Commun. 2002 May 24;293(5):1550-5.

Gardel, M. L., Nakamura, F., Hartwig, J., Crocker, J. C., Stossel, T. P., and Weitz, D. A. (2006). Stress-dependent elasticity of composite actin networks as a model for cell behavior. *Phys Rev Lett*, 96(8):088102. Gardel, M L Nakamura, F Hartwig, J Crocker, J C Stossel, T P Weitz, D A HL19429/HL/United States NHLBI Research Support, N.I.H., Extramural Research Support, Non-U.S. Gov't Research Support, U.S. Gov't, Non-P.H.S. United States Physical review letters Phys Rev Lett. 2006 Mar 3;96(8):088102. Epub 2006 Mar 3.

Kovar, D. R. and Pollard, T. D. (2004). Insertional assembly of actin filament barbed ends in association with formins produces piconewton forces. *Proc Natl Acad Sci U S A*, 101(41):14725–30. Kovar, David R Pollard, Thomas D GM-26132/GM/United States NIGMS GM-26338/GM/United States NIGMS Research Support, U.S. Gov't, P.H.S. United States Proceedings of the National Academy of Sciences of the United States of America Proc Natl Acad Sci U S A. 2004 Oct 12;101(41):14725-30. Epub 2004 Sep 17.

MacKintosh, F. C., Kas, J., and Janmey, P. A. (1995). Elasticity of semiflexible biopolymer networks. *Phys Rev Lett*, 75(24):4425–4428. Journal article Physical review letters Phys Rev Lett. 1995 Dec 11;75(24):4425-4428.

Marcy, Y., Prost, J., Carlier, M.-F., and Sykes, C. (2004). Forces generated during actin-based propulsion: a direct measurement by micromanipulation. *Proc Natl Acad Sci U S A*, 101(16):5992–5997.

Mogilner, A. (2006). On the edge: modeling protrusion. *Curr Opin Cell Biol*, 18(1):32–9. Mogilner, Alex U54 GM64346/GM/United States NIGMS Research Support, N.I.H., Extramural Research Support, U.S. Gov't, Non-P.H.S. Review United States Current opinion in cell biology Curr Opin Cell Biol. 2006 Feb;18(1):32-9. Epub 2005 Nov 28.

Mogilner, A. and Edelstein-Keshet, L. (2002). Regulation of actin dynamics in rapidly moving cells: a quantitative analysis. *Biophys J*, 83(3):1237–58. Mogilner, Alex Edelstein-Keshet, Leah United States Biophysical journal Biophys J. 2002 Sep;83(3):1237-58.

Paluch, E., van der Gucht, J., Joanny, J. F., and Sykes, C. (2006). Deformations in actin comets from rocketing beads. *Biophys J*, 91(8):3113–22. Paluch, Ewa van der Gucht, Jasper Joanny, Jean-Francois Sykes, Cecile Research Support, Non-U.S. Gov't United States Biophysical journal Biophys J. 2006 Oct 15;91(8):3113-22. Epub 2006 Jul 28.

Plastino, J., Lelidis, I., Prost, J., and Sykes, C. (2004). The effect of diffusion, depolymerization and nucleation promoting factors on actin gel growth. *Eur Biophys J*, 33(4):310–20. Plastino, Julie Lelidis, Ioannis Prost, Jacques Sykes, Cecile Comparative Study Evaluation Studies Research Support, Non-U.S. Gov't Germany European biophysics journal : EBJ Eur Biophys J. 2004 Jul;33(4):310-20. Epub 2003 Dec 9.

Sekimoto, K., Prost, J., Julicher, F., Boukellal, H., and Bernheim-Grosswasser, A. (2004). Role of tensile stress in actin gels and a symmetry-breaking instability. *Eur Phys J E Soft Matter*, 13(3):247–59. Sekimoto, K Prost, J Julicher, F Boukellal, H Bernheim-Grosswasser, A Comparative Study Evaluation Studies Validation Studies France The European physical journal. E, Soft matter Eur Phys J E Soft Matter. 2004 Mar;13(3):247-59.

Shafrir, Y. and Forgacs, G. (2002). Mechanotransduction through the cytoskeleton. *Am J Physiol Cell Physiol*, 282(3):C479–86. Shafrir, Yinon Forgacs, Gabor Research Support, U.S. Gov't, Non-P.H.S. United States American journal of physiology. Cell physiology Am J Physiol Cell Physiol. 2002 Mar;282(3):C479-86.

Tawada, K. and Sekimoto, K. (1991). Protein friction exerted by motor enzymes through a weak-binding interaction. *J Theor Biol*, 150(2):193–200.

Trichet, L., Campas, O., Sykes, C., and Plastino, J. (2007). Vasp governs actin dynamics by modulating filament anchoring. *Biophys J*, 92(3):1081–9. Trichet, Lea Campas, Otger Sykes, Cecile Plastino, Julie Research Support, Non-U.S. Gov't United States Biophysical journal Biophys J. 2007 Feb 1;92(3):1081-9. Epub 2006 Nov 10.

Tsuda, Y., Yasutake, H., Ishijima, A., and Yanagida, T. (1996). Torsional rigidity of single actin filaments and actin-actin bond breaking force under torsion measured directly by in vitro micromanipulation. *Proc Natl Acad Sci U S A*, 93(23):12937–42. Tsuda, Y Yasutake, H Ishijima, A Yanagida, T United states Proceedings of the National Academy of Sciences of the United States of America Proc Natl Acad Sci U S A. 1996 Nov 12;93(23):12937-42.

van der Gucht, J., Paluch, E., Plastino, J., and Sykes, C. (2005). Stress release drives symmetry breaking for actin-based movement. *Proc Natl Acad Sci U S A*, 102(22):7847–52. van der Gucht, Jasper Paluch, Ewa Plastino, Julie Sykes, Cecile Comparative Study Research Support, Non-U.S. Gov't United States Proceedings of the National Academy of Sciences of the United States of America Proc Natl Acad Sci U S A. 2005 May 31;102(22):7847-52. Epub 2005 May 23.
