# Supplementary material for: In Silico Reconstitution of Actin-Based Symmetry Breaking and Motility
Source: PLoS Biol. 2009 Sep 22;7(9):e1000201. doi: 10.1371/journal.pbio.1000201 (PMC2738636; doi:10.1371/journal.pbio.1000201)
Supplement: Table S3 — Corresponding simulation parameter names in the main text and in the code. (0.04 MB DOC) [file pbio.1000201.s027.doc]

| Parameter in text | Parameter in Model | Description |
| --- | --- | --- |
|  |  |  |
| P_XL_ | P_XLINK | Probability of forming crosslink |
| F_L_ | LINK_FORCE | Spring constant for node-node links |
| F_BL_ | LINK_BREAKAGE_FORCE | Force threshold above which node-node links break |
| M_R_ | NODE_REPULSIVE_MAG | Magnitiude of node repulsive force |
